# The *Gonium pectorale* genome demonstrates co-option of cell cycle regulation during the evolution of multicellularity

**DOI:** 10.1038/ncomms11370

**Published:** 2016-04-22

**Authors:** Erik R. Hanschen, Tara N. Marriage, Patrick J. Ferris, Takashi Hamaji, Atsushi Toyoda, Asao Fujiyama, Rafik Neme, Hideki Noguchi, Yohei Minakuchi, Masahiro Suzuki, Hiroko Kawai-Toyooka, David R. Smith, Halle Sparks, Jaden Anderson, Robert Bakarić, Victor Luria, Amir Karger, Marc W. Kirschner, Pierre M. Durand, Richard E. Michod, Hisayoshi Nozaki, Bradley J. S. C. Olson

**Affiliations:** 1 Department of Ecology and Evolutionary Biology, University of Arizona, Tucson, Arizona 85712, USA; 2 Division of Biology, Kansas State University, Manhattan, Kansas 66506, USA; 3 Donald Danforth Plant Science Center, St Louis, Missouri 63132, USA; 4 Center for Advanced Genomics, National Institute of Genetics, Mishima, Shizuoka 411-8540, Japan; 5 Center for Information Biology, National Institute of Genetics, Mishima, Shizuoka 411-8540, Japan; 6 Department of Evolutionary Genetics, Max Planck Institute for Evolutionary Biology, 24306 Plön, Germany; 7 Department of Biological Sciences, Graduate School of Science, University of Tokyo, Bunkyo-ku, Tokyo 113-003, Japan; 8 Department of Biology, Western University, London, Ontario, Canada N6A 5B7; 9 Division of Molecular Biology, Ruđer Bošković Institute, Zagreb 10000, Croatia; 10 Department of Systems Biology, Harvard Medical School, Boston, Massachusetts 02115, USA; 11 Kavli Institute for Theoretical Physics, University of California Santa Barbara, Santa Barbara, California 93106, USA; 12 Research Computing Division, Harvard Medical School, Boston, Massachusetts 02115, USA; 13 Department of Molecular Medicine and Haematology, Faculty of Health Sciences and Evolutionary Studies Institute, University of the Witwatersrand, Johannesburg 2000, South Africa; 14 Department of Biodiversity and Conservation Biology, University of the Western Cape, Cape Town 7535, South Africa

## Abstract

The transition to multicellularity has occurred numerous times in all domains of life, yet its initial steps are poorly understood. The volvocine green algae are a tractable system for understanding the genetic basis of multicellularity including the initial formation of cooperative cell groups. Here we report the genome sequence of the undifferentiated colonial alga, *Gonium pectorale,* where group formation evolved by co-option of the retinoblastoma cell cycle regulatory pathway. Significantly, expression of the *Gonium* retinoblastoma cell cycle regulator in unicellular *Chlamydomonas* causes it to become colonial. The presence of these changes in undifferentiated *Gonium* indicates extensive group-level adaptation during the initial step in the evolution of multicellularity. These results emphasize an early and formative step in the evolution of multicellularity, the evolution of cell cycle regulation, one that may shed light on the evolutionary history of other multicellular innovations and evolutionary transitions.

## Abstract (web summary)

The undifferentiated *Gonium pectorale* represents the initial transition to multicellularity. Here, Bradley Olson, Erik Hanschen and colleagues describe the genome of *Gonium pectorale*, demonstrating that co-option of the retinoblastoma cell cycle regulatory pathway was a key genetic change in the evolution of multicellularity.

## Graphical abstract

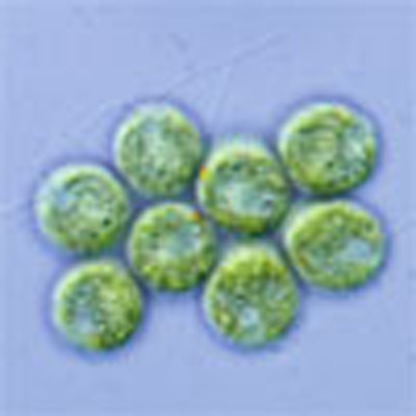

Multicellular organisms have independently evolved numerous times throughout the tree of life including plants, animals, fungi, cyanobacteria, amoeba, brown algae, red algae and green algae^1^,^2^. In animals, multicellularity emerged 600–950 million years ago (Myr ago) correlating with a large expansion of genes encoding transcription factors, signalling pathways, and cell adhesion genes that were co-opted from their unicellular ancestors^3^,^4^ Similarly, multicellular, terrestrial plants emerged ∼750 Myr ago correlating with an expansion of many signalling pathways present in their unicellular relatives^5^,^6^. However, because most multicellular lineages have long diverged from their unicellular relatives, the genomic signature of the transition to multicellularity has been obscured, and consequently this evolutionary process remains enigmatic.

The volvocine green algae are a unique model system for the evolution of multicellularity because the unicellular ancestry is clear, the emergence of multicellularity occurred ∼230 Myr ago, and species exhibit a stepwise increase in morphological complexity ranging from undifferentiated colonies to differentiated multicellular species^7^,^8^ (Fig. 1, Supplementary Figs 1,2). Unicellular *Chlamydomonas reinhardtii* is thought to resemble the unicellular ancestor of multicellular volvocines, including undifferentiated *Gonium pectorale* and differentiated *Volvox carteri* (Fig. 1).

*Chlamydomonas* undergoes a variant cell cycle (Supplementary Fig. 2), regulated by homologues of the retinoblastoma cell cycle pathway, termed multiple-fission where it divides by a series of rapid cell divisions producing individual daughter cells^9^,^10^,^11^. *Gonium* typically forms 8- or 16-celled undifferentiated colonies, where each constituent cell resembles a *Chlamydomonas* cell (Fig. 1). *Gonium* also undergoes multiple fission forming daughter colonies by keeping cells attached after multiple-fission, suggesting either cell cycle regulation^12^, or cell–cell adhesion has been modified to promote multicellularity. In *Gonium*, like *Chlamydomonas*, growth and cell division are uncoupled^13^,^14^. Asexual juvenile *Gonium* colonies grow (without cell division) into adults. After cell division through multiple-fission, juvenile colonies hatch forming 8 or 16 daughter colonies of 8 or 16 cells (Supplementary Fig. 2). *Volvox* contains approximately 2,000 small, terminally differentiated, somatic cells on the surface of the spheroid and approximately 16 large reproductive cells embedded in extracellular matrix (ECM) inside the spheroid (Fig. 1). *Volvox* also has modified multiple fission where germ–soma separation is established after an asymmetric cell division^8^,^14^.

The transition to multicellularity in the Volvocales was thought to involve at least 12 steps (Supplementary Fig. 1)^15^,^16^ though the genetic basis of these steps remains enigmatic. Genomic comparison of the extremes of morphological complexity, *Chlamydomonas* and *Volvox*, suggests few genetic changes are required^17^, but it is unclear how and when the genes important for multicellularity evolved during these 12 steps^14^.

By sequencing the genome of the undifferentiated, chlorophycean *Gonium pectorale*, a species without a differentiated ancestor^16^, we find co-option of cell cycle regulation, which occurred during the initial transition to cell groups, as the genetic basis for the evolution of multicellularity. The cell cycle regulation found in undifferentiated *Gonium*, co-opted in a multicellular context and shared with germ–soma-differentiated *Volvox*, indicates group-level adaptations in undifferentiated colonies. The early co-option of cell cycle regulation for group-level life cycle and reproduction is a critical and formative step in the evolution of multicellularity.

## Results

### Genomic comparisons of volvocine algae

At the genomic level, the genomes of *Chlamydomonas*, *Gonium* and *Volvox* are similar, though various measures of genome compactness correlate with cell number, consistent with a long-term increase in organismal size^18^,^19^. *Chlamydomonas* and *Gonium* have similar GC content near 64%, while *Volvox* has 56% GC (Table 1). Otherwise, *Chlamydomonas*, *Gonium* and *Volvox* have decreasing gene densities of 159.6, 120.9 and 113.7 genes per megabase, with an increasing average intron length of 279, 349 and 500 base pairs, respectively. Although intron length increases with organismal complexity, the number of introns per gene (*Chlamydomonas,* 7.46; *Gonium*, 6.5; *Volvox* 6.8, Table 1) does not. GC content, intron length and gene density correlate with morphological complexity.

We next examined genome-wide evolution in all three species to better understand the genetic basis for the evolution of multicellularity. A prediction of lineage-specific genes^20^ shows few genes correlate with the evolution of multicellularity in the Volvocales (phylostratum 7; PS7) with a maximum of 180–357 genes (Fig. 2a). This suggests that the evolution of multicellularity does not rely upon the evolution of *de novo* genes. Though gene regulation may be important during multicellular innovation, the diversity and abundance of transcription factors is similar in *Chlamydomonas*, *Gonium* and *Volvox* (Fig. 2b, Supplementary Table 1, Supplementary Fig. 3). Although enrichment of transcription factors can correlate with the evolution of multicellularity^3^, this is not always the case^21^; we found that *Gonium* and *Volvox* have fewer transcription factors than in *Chlamydomonas* (Fig. 2b). These include PHD domains (transcription and chromatin binding), DNA binding transcription factors and histones (Fig. 2b, Supplementary Tables 2 and 3, Supplementary Fig. 3).

Using all published chlorophyte green algae genomes, we constructed Markov-based gene families (*Chlamydomonas*, 73%; *Gonium*, 73%; *Volvox*, 70% of genes in gene families of size greater than one). Compared with 2,844 net gene families gained, which correlate with the origin of the Chlorophyceae, a phylogenetic analysis of these gene families suggests little protein innovation (110 net gene families) during the evolution of multicellularity (Fig. 2c). These same green algae genomes allowed analysis into Pfam domain innovation, which may correlate with the evolution of multicellularity. We found innovation of only nine Pfam domains correlating with the evolution of multicellularity (Fig. 2d, Supplementary Table 4). Moreover, multicellular algae (*Gonium* and *Volvox*) have reduced Pfam domain diversity and abundance (compared with nine unicellular green algae, Supplementary Data 1); 394 Pfam A domains are significantly under-represented versus 129 over-represented Pfam domains (Supplementary Fig. 4). Interestingly, there is an excess of species-specific genes (Fig. 2a,c) and Pfam domains (Fig. 2d) compared with multicellularity-correlated genes and Pfam domains, suggesting that species-specific adaptations are more numerous than changes correlating with the evolution of multicellularity. We observe more evidence of species-specific, rather than multicellular-specific, protein innovations, suggesting species-specific adaptation (Fig. 2a,c,d, Supplementary Tables 5–8) rather than genome-wide differences correlating with the evolution of multicellularity. The evolution of multicellularity in the volvocine algae does not require large-scale genomic innovation.

### Co-option of cell cycle regulation for multicellularity

Notably, we observe that the genetic innovation correlating with multicellularity, shared between *Gonium* and *Volvox*, evolved through co-option of existing developmental programs of cell cycle control. Volvocine algae have a common multiple-fission life cycle, with variation in timing and number of divisions (Supplementary Fig. 1)^14^. Like most eukaryotes, including plants and animals, their cell cycles are regulated by homologues of the retinoblastoma cell cycle regulatory pathway (Fig. 3, Supplementary Figs 5–8, Supplementary Table 9)^9^,^10^, in which cyclin-dependent kinases (CDKs) bind cyclin proteins to phosphorylate and regulate retinoblastoma (*RB* or *MAT3* in the Volvocales), which in turn de-represses the cell cycle (Fig. 3a). Although most of these regulators are nearly identical in *Chlamydomonas*, *Gonium* and *Volvox* (Fig. 3b,e), there are two notable differences. First, *Volvox* has a four gene expansion of cyclin D1 genes (Fig. 3c)^17^. As *Volvox* has tissue differentiation, these cyclin D1 genes may have been important for tissue development as is the case in metazoans and land plants^22^,^23^, supported by the fact that *RB* has moved into the mating locus of *Gonium* and *Volvox* and is differentially expressed between mating loci (Fig. 3d)^24^,^25^. However, the tandem array expansion of the cyclin D1 genes is also found in *Gonium* (Fig. 3c), where cyclin D genes display elevated dN/dS ratios compared with other cell cycle regulators (Supplementary Fig. 8), suggesting the function of these cyclin Ds may be important for the transition to undifferentiated colonies, rather than tissue differentiation.

Second, there is modification of the *RB* gene in *Gonium* and *Volvox* (Fig. 3d, Supplementary Figs 5–7). Protein dimers of cyclins and CDKs primarily regulate *RB* by phosphorylating serine or threonine residues, which is thought to regulate *RB* binding of chromatin via E2F/DP transcription factors^11^,^26^,^27^. Recently it has been shown in human cells that cyclin D and CDK4/6 regulate monophosphorylation of *RB* proteins for G_1_ phase-specific *RB* functions^27^. If similar in *Gonium*, this would suggest a role of the expanded cyclin D1 genes for regulating *RB* to express multicellularity-related genes during G_1_ phase. If the expanded cyclin D1 proteins found in *Gonium* regulate multicellular cell cycle changes, modification of cyclin D-CDK phosphorylation sites in *RB* is predicted. Indeed, the linker of the E2F/DP binding pocket of *RB* is shorter in *Gonium* and *Volvox* compared with *Chlamydomonas* (Fig. 3d, Supplementary Figs 5 and 6), potentially altering how *RB* binds to chromatin via E2F/DP. In addition, phosphorylation sites between the E2F/DP pocket region (RB-A and RB-B domains) and the conserved carboxy (C)-terminal domain are absent in *Gonium* and *Volvox RB* proteins (Fig. 3d). Interestingly, in animals the C terminus of *RB* is intertwined with E2F/DP and changes in the phosphorylation by cyclin–CDK complexes could also alter E2F/DP binding^28^. As these phosphorylation sites are absent in *Gonium* and *Volvox RB* proteins (Fig. 3d), this suggests that *RB* co-option for multicellularity may result in differences in locus-specific temporal expression of genes important for multicellularity during G_1_, such as cell–cell adhesion genes. Given the role the *RB* pathway plays in regulating the cell cycle in *Chlamydomonas*, its early modification found in *Gonium*, and its co-option for complex morphology in *Volvox*, *RB* pathway regulation might be a key step towards multicellularity in the volvocine algae.

To test whether *RB* modifications present in *Gonium* and *Volvox* (compared with *Chlamydomonas*) are unrelated to, cause or are a consequence of multicellularity, we expressed the *Chlamydomonas*^11^ and *Gonium RB* genes in a *Chlamydomonas* strain lacking its *RB* gene (*rb*, *mat3–4* strain, Fig. 4a)^9^,^11^ using the promoter and terminator from the *Chlamydomonas RB* gene to ensure expression near wild-type levels (Fig. 4b,c)^11^. The *Chlamydomonas RB* gene rescues the small cell size defect in the *rb* mutant (*HA-CrRB::rb*, Fig. 4a), while the *Gonium RB* gene rescues the cell size defect and causes the *Chlamydomonas rb* mutant to become non-palmelloid colonial, ranging from 2 to 16 normal-sized cells (*HA-GpRB::rb*, Fig. 4a). Crossing *RB* gain-of-function transformed *Chlamydomonas* strain to a *Chlamydomonas* strain lacking *DP1*, a gene that dimerizes with *E2F* to anchor *RB* to chromatin^11^, results in suppression of the colonial phenotype and large-sized cells (*HA-GpRB::rb::dp1*, Fig. 4a) consistent with the phenotype of the *Chlamydomonas dp1* mutant itself^10^. This demonstrates that the *Gonium RB* gene causes colonial multicellularity (Fig. 4a) through the *RB* pathway (Fig. 3a) and suggests differences in how *RB* binds chromatin and regulates the expression of cell cycle related genes in *Gonium* and *Volvox* are important for co-option of these *RB* targeted genes for multicellularity (Figs 3 and 4). This gain-of-function demonstrates a causal link between cell cycle regulation and the group level during the evolution of multicellularity, emphasizing that multicellularity can evolve by co-option and modification of regulatory genes rather than extensive genomic differences or innovation.

### *Volvox* innovations for morphological complexity

**Figure 1:**
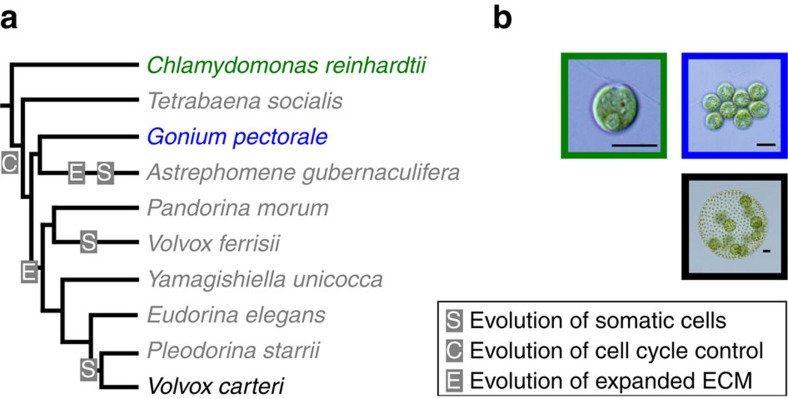
Volvocine phylogenetic tree. (**a**) Evolution of cell cycle control (C), expanded ECM (E) and somatic cells (S) are denoted. (**b**) Micrographs of *Chlamydomonas* (green; scale bar, 10 μm), *Gonium* (blue; scale bar, 10 μm) and *Volvox* (black; scale bar, 25 μm) show morphological differences.

**Figure 2:**
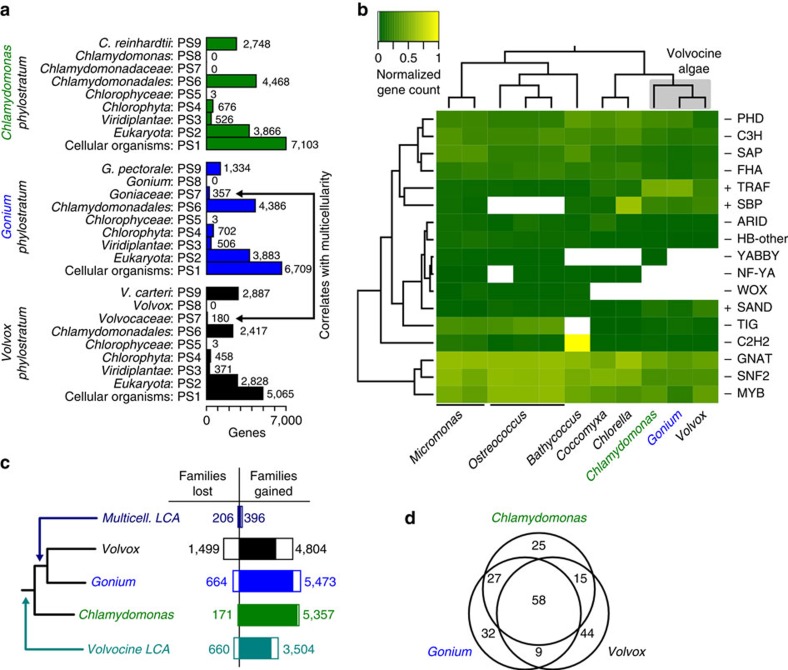
Genome similarity in the Volvocales. (**a**) Predicted number of genes in each phylostratum (PS1–PS9) for *Chlamydomonas*, *Gonium* and *Volvox*. (**b**) Heatmap of transcription factor abundance for all green algae. Significant over- (+) and under-representation (−) in colonial/multicellular lineages (*Gonium* and *Volvox*) is denoted (G test of independence, *α*=0.05). Rows are clustered (left), an accepted phylogeny is depicted (top). (**c**) Phylogenetic analysis of gene family evolution. Bars to the left and right of the vertical axis denote the lost and gained gene families respectively, relative to its parental node. (**d**) Venn diagram of the species distribution of Pfam A domains unique to the volvocine algae.

In *Volvox*, somatic differentiation is causally regulated by the *regA* gene cluster, a set of putative DNA-binding transcription factors thought to regulate chloroplast biogenesis^29^,^30^,^31^. The *regA* gene cluster is absent in *Chlamydomonas* and *Gonium* (Fig. 5a,b), but is present in diverse *Volvox ferrisii* and *Volvox gigas*^32^, suggesting early evolution and co-option of this cluster shortly after the split of *Gonium* and *Volvox* lineages (Fig. 1)^32^. Interestingly, if the absence of *regA* in *Gonium* is indicative of the absence of *regA* in *Astrephomene*, with an independent evolution of somatic cells (Fig. 1)^16^, *Astrephomene* may determine somatic cell fate through a different pathway than *Volvox* suggesting multiple evolutionary pathways and subsequent evolutionary consequences during the evolution of multicellularity. Indeed, undifferentiated multicellularity evolved once in the Volvocales^16^, while additional morphological complexity (for example, cellular differentiation and large *Volvox* body size) has repeatedly evolved, suggesting a relative ease to gain and lose additional complexity.

We investigated proteins related to morphological complexity, pherophorins and matrix metalloprotease (MMP) proteins, in the volvocine algae^17^. These proteins are hypothesized to produce ECM and break up cell wall components during reproduction in *Gonium* and other Volvocales^15^,^33^,^34^. While *Chlamydomonas* contains no ECM and *Gonium* contains little ECM, a *Volvox* spheroid is largely composed of ECM (Fig. 1). Pherophorin and MMP gene families are expanded in *Volvox* relative to *Chlamydomonas* (Fig. 5c)^17^. We found the expansion of pherophorins and MMP genes in *Volvox* (Fig. 5c, Supplementary Data 2 and 3) is not present in *Gonium*, though some species-specific expansion of MMP genes has occurred (Supplementary Data 2 and 3). While some expansion of ECM gene families in *Gonium* was expected^14^ to direct the cell wall layer synthesis of a *Gonium* colony, this layer may instead be directed through differential gene expression. Pherophorin, MMP expansion and cellular differentiation correlate with expanded organismal size rather than the origin of multicellularity, suggesting a subsequent step in the evolution of multicellularity.

## Discussion

We have investigated the evolution of multicellularity in the volvocine algae by sequencing the genome of the undifferentiated *Gonium.* Despite morphological differences, it was known that the *Chlamydomonas* and *Volvox* genomes are strikingly similar, suggesting that multicellularity required few genetic innovations^17^,^35^. However, these two genomes, positioned at the extremes of volvocine morphology, were unable to resolve the tempo and mode^36^ of the evolutionary transition to multicellularity.

**Figure 3:**
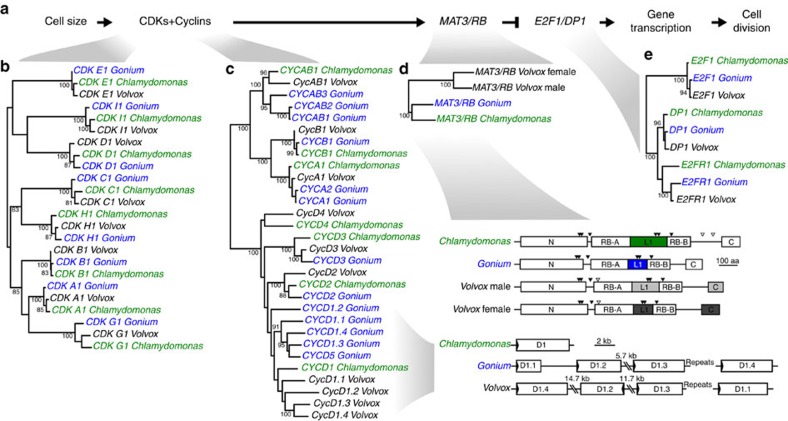
Cell cycle pathway evolution. (**a**) The retinoblastoma cell cycle regulatory pathway. (**b**) Phylogeny of CDK genes. (**c**) Phylogeny of cyclin genes and syntenic relationships of cyclin D1 genes. (**d**) Phylogeny of *MAT3/RB* genes and comparison of *MAT3/RB* proteins. Domains RB-A and RB-B, the linker region in the binding pocket (L1), N-terminal conservation (N) and C-terminal conservation (C) are shown. Difference in shade of grey for *Volvox* male and *Volvox* female indicates sex-specific divergence^24^. Conserved putative CDK phosphorylation sites are indicated with solid arrows, species-specific sites are indicated with open arrows. (**e**) E2F/DP1 genes. All trees have a midpoint root and bootstrap values above 80% are indicated.

**Figure 4:**
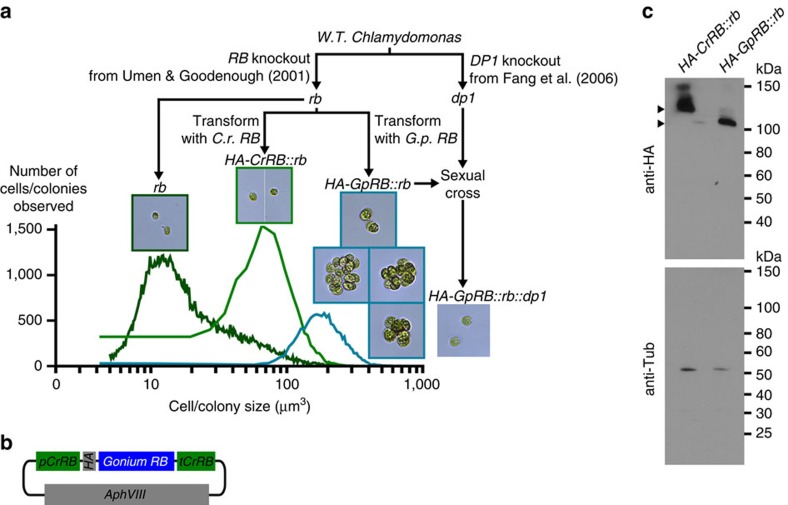
*Gonium RB* causes a colonial phenotype when expressed in *Chlamydomonas*. (**a**) Transformation schematic showing resulting morphology overlaid onto cell and colony size measurements (logarithmic scale) of control *Chlamydomonas RB* mutant (*rb* or *mat3*–*4*, transformed with empty vector), complementing *HA-CrRB::rb* (two of five independent transformations are shown) and colonial *HA-GpRB::rb* (four independent transformations are shown). Crossing colonial *HA-GpRB::rb* to a *Chlamydomonas DP1* mutant (*dp1*) restores unicellularity in *Chlamydomonas* (one of two independent matings are shown). (**b**) Schematic *Gonium RB* tagged with 3XHA with its expression driven by the *Chlamydomonas RB* promoter and terminator^11^. (**c**) Anti-HA immunoblotting of *HA-CrRB::rb* and *HA-GpRB::rb* with anti-tubulin loading controls. Arrows indicate proteins at their expected molecular mass.

**Figure 5:**
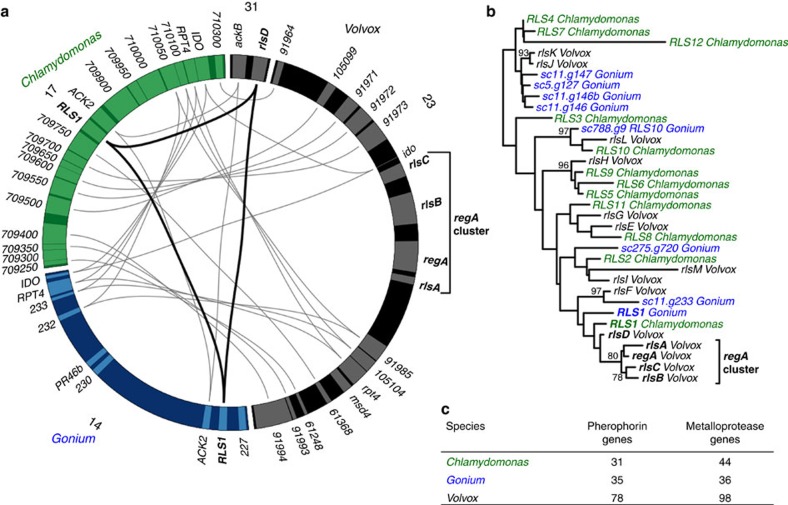
Genetic changes present in the *Volvox* genome. (**a**) Gene synteny near the *regA* gene cluster and closely related *regA*-like genes (bold). Chromosome or scaffold number is indicated. Conserved genes are linked by line segments for *regA*-like (thick, black) and neighbouring genes (thin, grey). (**b**) Phylogenetic relationships of *regA*-like genes. The tree is a midpoint root and bootstrap values above 70% are indicated. (**c**) Comparison of number of pherophorin and metalloprotease genes in *Chlamydomonas*, *Gonium* and *Volvox*.

**Figure 6:**
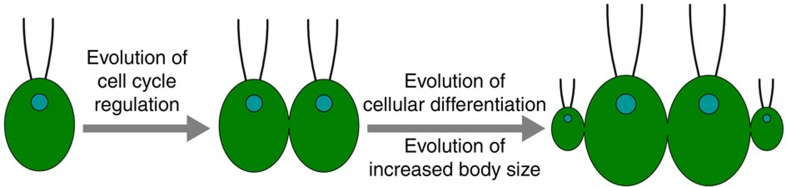
Conceptual model for the evolution of multicellularity. Multicellularity hinges on the evolution of cell cycle regulation in a multicellular context with subsequent evolution of cellular differentiation (here, cell size-based) and increased body size.

The evolution of multicellularity in the volvocine algae is thought to involve 12 morphological innovations (Supplementary Fig. 1)^15^. Five of these steps correlate with the evolution of cell groups^7^, a period of rapid evolutionary change (tempo). This view emphasizes the importance, and subsequent modification, of innovations correlating with undifferentiated colonies (mode). Finding support for this view, we have generalized these 12 steps into three major phases (Fig. 6): the evolution of cell cycle regulation to form cooperative groups via cell–cell adhesion, the evolution of increased organismal size, and the evolution of differentiated germ and soma cells. Having sequenced the genome of *Gonium*, along with the published genomes of *Chlamydomonas* and *Volvox*, we can now identify the genetic pathways associated with each of these steps. The evolution of undifferentiated colonies correlates with *RB* cell cycle regulatory pathway evolution (Figs 3 and 4), which is further modified as complexity increases in the Volvocales^24^. Increased organismal size toward *Volvox* correlates with an expansion of pherophorins and MMPs (Fig. 5c). Finally the evolution of the *regA* gene cluster underlies somatic differentiation (Fig. 5a,b). Future sequencing of additional Volvocales genomes should clarify the evolutionary steps required for the evolution of germ and soma. Our three-phase model for the emergence of multicellularity, supported by the genetic pathways important for their evolution, changes our understanding of the tempo and mode of multicellular evolution previously obscured in other taxa such as plants, fungi and animals due to genomic divergence (Fig. 6).

Interestingly, an emerging theme throughout the evolution of multicellularity is that the genetic basis for the evolutionary transition emerges much earlier than anticipated^3^,^6^,^32^. In plants and animals, *RB* proteins are important for regulating both cell proliferation and differentiation by highly complex locus interactions with chromatin and chromatin remodelling factors^37^,^38^. Our finding that the *RB* pathway was co-opted early for multicellularity in undifferentiated colonies suggests that the template for subsequent evolutionary innovations in developmental programs was laid out during the transition to undifferentiated multicellularity via *RB* and cell cycle modifications, rather than with emergence of germ and somatic cellular differentiation. Interestingly, *RB* has been further co-opted for a role in sexual differentiation in *Volvox*, where there are male- and female-specific isoforms of *RB*^24^. This suggests that the evolution of multicellular cell cycle regulation was a critical step for the evolution of multicellularity. By comparing the genomes of these three volvocine green algae, we have determined that the mechanism of multicellular evolution is primarily co-option and regulatory modification of existing genetic pathways^39^. Gene duplication forms the basis of subsequent multicellular innovations.

The genomic age is illuminating the genetic pathways that are important for the evolution of multicellularity in other organisms where genes such as cadherins and integrins in animals^3^,^4^ and cell wall biogenesis genes in plants^6^. These are roughly analogous to metalloproteases and pherophorins in the volvocine algae highlighting convergences on similar genetic innovations for multicellularity. The substantial innovation and expansion of transcription factors and signalling networks found in animals and plants^3^,^6^ is not present in the volvocine algae. However, the volvocine algae demonstrate the critical role of transcriptional regulation of the cell cycle by *RB* for the formation of undifferentiated colonies. *RB* proteins regulate the cell cycle of most eukaryotes^11^,^26^, and are tumour suppressors in humans^26^, suggesting a broader role for *RB* and cell cycle regulation during the evolution of multicellularity.

The implications of these findings are greater than simply identifying when genes evolved during the evolution of multicellularity. Theoretical work has emphasized the need for greater understanding of the origin of an integrated group life cycle during the evolution of multicellularity^12^,^40^,^41^,^42^. The field has been concerned with the evolution of germ–soma division of labour as the defining step in the evolution of multicellularity^40^,^43^,^44^,^45^; indeed, a recent review of animal multicellularity^45^ does not mention the importance of cell cycle regulation and group formation. The *Gonium* genome reflects the early evolution of cell cycle regulation (Figs 3 and 4) in undifferentiated groups, conserved and modified in differentiated *Volvox*, that is indicative of the emergence of colony level adaptations. We highlight an early and formative step, the co-option and expansion of cell cycle regulation, as important for the evolution of cooperative groups and impacting the evolution of more complicated body plans; one that may shed light on the evolutionary history of other multicellular innovations and evolutionary transitions.

## Methods

### Strain and genome sequencing

The *Gonium pectorale* strain K3-F3-4 (mating type minus, NIES-2863 from the Microbial Culture Collection at National Institute for Environmental Studies, Tsukuba, Japan, http://mcc.nies.go.jp/) was used for genome sequencing. *Gonium* was grown in 200–300 ml VTAC media at 20 °C with a 14:10 h light–dark cycle using cool-white fluorescent lights (165–175 μmol m^−2^ s^−1^).

For next-generation sequencing and construction of a fosmid library, total DNA was extracted. Sequencing libraries were prepared using the GS FLX Titanium Rapid Library Preparation Kit (F. Hoffmann-La Roche, Basel, Switzerland) and the TruSeq DNA Sample Prep Kit (Illumina Inc., San Diego, CA, USA) and were run on both GS FLX (F. Hoffmann-La Roche) and MiSeq (Illumina Inc.) machines. Newbler v2.6 was used to assemble the GS FLX reads. A fosmid library was constructed in-house using vector pKS300. The fosmid library (23,424 clones) and BAC library (18,048 clones, Genome Institute (CUGI), Clemson University, Clemson, SC, USA) were end-sequenced using a BigDye terminator kit v3 (Life Technologies, Carlsbad, CA, USA) analysed on automated ABI3730 capillary sequencers (Life Technologies).

### Evidence-based gene prediction

Introns hint file generation was done through a two-step, iterative mapping approach using Bowtie/Tophat command lines and custom Perl scripts written by Mario Stanke as part of AUGUSTUS^46^, (available at: http://bioinf.uni-greifswald.de/bioinf/wiki/pmwiki.php?n=IncorporatingRNAseq.Tophat). AUGUSTUS version 2.6.1 was selected because its algorithm has been successfully tuned to predict genes in *Chlamydomonas* and *Volvox* genomes, which contain high GC content^46^. Reads were first mapped to the genome assembly with Tophat version 2.0.2 (ref. 47) and the raw alignments were filtered to create an initial (intron) hints file, which was subsequently provided to AUGUSTUS during gene prediction. An exon–exon junction database was generated from the initial AUGUSTUS prediction via a Perl script. The twice-mapped reads (once to the genome and once to the exon–exon sequences) were then merged, filtered and a final intron hints file was created. From this, the final gene prediction with AUGUSTUS was performed.

### Pfam domain analysis

Diversity and abundance of Pfam domains was determined for all published green algae genomes. Chlorophyte genomes including *Bathycoccus prasinos*^48^, *Chlamydomonas reinhardtii*^35^, *Chlorella variabilis*^49^, *Coccomyxa subellipsoidea* C-169 (ref. 50), *Micromonas pusilla* CCMP1545 (ref. 51), *Micromonas pusilla* RCC299 (ref. 51), *Ostreococcus tauri*^52^, *Ostreococcus lucimarinus*^53^, *Ostreococcus* sp. RCC809 (US Department of Energy, Phytozome) and *Volvox carteri* (both versions 1 and 2; ref. 17) were searched using direct submission of Pfam A and Pfam B domains using Bioperl. Subsequent hits were counted and produced a matrix of Pfam domain diversity and abundance across green algae.

### Analysis of transcription-associated proteins

Transcription-associated proteins (TAPs) include transcription factors (enhance or repress transcription) and transcription regulators (proteins which indirectly regulate transcription such as scaffold proteins, histone modification or DNA methylation). We combined three TAP classification rules for plants; PlantTFDB^54^, PlnTFDB^55^ and PlanTAPDB^56^ to make a set of classification rules for 96 TAP families. Conflicts between the three sets of rules were manually resolved using the rule that included more genes as transcription-associated proteins.

Each transcription family includes at least one, up to three, mandatory domains. Families may include up to six forbidden domains (that is, a gene *G* cannot be in family *F* if domain *D* is present); not all families have defined forbidden domains. All mandatory and forbidden domains were represented by a full-length, global, Hidden Markov Model (HMM). Available HMMs were retrieved from Pfam_ls database^57^,^58^. When HMMs were not available from the Pfam_ls database, custom HMMs were made using multiple sequence alignments from PlnTFDB^55^ and the HMM was calculated using HMMER version 3.0 (ref. 59) using ‘hmmbuild' with default parameters and ‘hmmcalibrate—seed 0′.

Gathering cutoff thresholds (GA) for the custom HMMs were set as the lowest score of a true positive hit using a ‘hmmscan' search against several complete Chlorophyte genomes. Chlorophyte genomes including *Bathycoccus prasinos*^48^, *Chlamydomonas reinhardtii*^35^, *Chlorella variabilis*^49^, *Coccomyxa subellipsoidea* C-169 (ref. 50), *Micromonas pusilla* CCMP1545 (ref. 51), *Micromonas pusilla* RCC299 (ref. 51), *Ostreococcus tauri*^52^, *Ostreococcus lucimarinus*^53^, *Ostreococcus* sp. RCC809 (available on the DOE Phytozome website, version 10.1) and *Volvox carteri*^17^ were searched using ‘hmmscan' to search the library of 103 domains against the predicted protein sequences. Analyses were replicated with both *Volvox* version 1 and version 2; however, as results were not qualitatively different, results from version 1 are provided (Supplementary Fig. 3). Subsequent hits were classified into a TAP family. Conflicts between multiple TAP families were resolved by assigning the gene to the TAP family with the highest score (Supplementary Table 1).

### Construction of protein families

Protein families were created using OrthoMCL^60^ with a variety of inflation values ranging from 1.2 to 4.0 in steps of 0.1 (Supplementary Figs 16–17). This analysis was performed using Chlorophyte genomes available on the DOE JGI Phytozome website, version 10.1 including *Bathycoccus prasinos*^48^, *Chlamydomonas reinhardtii*^35^, *Chlorella variabilis*^49^, *Coccomyxa subellipsoidea* C-169 (ref. 50), *Micromonas pusilla* CCMP1545 (ref. 51), *Micromonas pusilla* RCC299 (ref. 51), *Ostreococcus tauri*^52^, *Ostreococcus lucimarinus*^53^, *Ostreococcus* sp. RCC809 (available on the DOE Joint Genome Institute website) and *Volvox carteri*^17^. This analysis was repeated for both *Volvox* version 1 and *Volvox* version 2. The inflation value of 1.9 was used for both analyses for consistency and was chosen to have relatively large, coarser grained clusters that were robust to higher inflation values (Supplementary Figs 16–19). To avoid bias introduced by not including all genes for each species, genes not assigned to a gene family (singletons) were assigned to single gene families and included in all subsequent phylogenetic gene family analyses.

A species tree was calculated by extracting OrthoMCL gene families containing only one copy in each species, for a total of 1,457 genes. The OrthoMCL run with an inflation value of 1.5 was chosen to use larger, coarser grained clusters, thus increasing the likelihood of capturing true 1:1:1 orthologues. This species tree included *Volvox carteri* version 2. These genes were independently aligned using Muscle version 3.8.31 (ref. 61) and concatenated. A phylogenetic tree was produced using RAxML version 8.0.20 (ref. 62) using the Protein Gamma model with automatic model selection on a per gene basis via partitions for each protein. A rapid bootstrapping analysis to search for the best-scoring ML tree was run with 100 bootstraps. The resulting species tree is consistent with previous results^16^,^51^,^63^,^64^,^65^ and had 100 bootstrap support at every node (Supplementary Fig. 20). This result is also consistent with numerous morphological characteristics supporting a closer relationship of *Gonium* and *Volvox*^66^.

Gene family evolution within the volvocine algae was analysed using Count version 10.04 (ref. 67) to perform several parsimony analyses including symmetric Wagner parsimony (each gene family may be gained or expanded multiple times and the gain penalty is equal to the loss penalty) and asymmetric Wagner parsimony (each gene family may be gained or expanded multiple times and the gain penalty is two times higher than the loss penalty). This analysis was repeated for both *Volvox* version 1 and version 2 genomes (Supplementary Tables 5–8).

### dN/dS analysis

During our OrthoMCL construction of protein gene families, we identified 6,154 clusters with exactly one copy in *Chlamydomonas* (version 5.3), *Gonium* and *Volvox* (version 2). The number of genes from other unicellular (non-*Chlamydomonas*) Chlorophyte species was ignored. This criteria is relatively strict as it does not include any genes with a duplicate in any species (copy number greater than one in any species) or any genes which are not essential (no copy present in any species) resulting in 1:1:1 orthologues. Given the relatively high gene duplication rates in volvocine algae (data not shown), these strict criteria support an interpretation of 1:1:1 orthology. Genome-wide pairwise comparisons of dN, dS and dN/dS were calculated (Supplementary Fig. 21; Supplementary Table 11) using PAML and codeml (ML analysis^68^) based on nucleotide translation based alignments (proteins were aligned using MUSCLE^61^).

### Prediction of lineage-specific genes

The phylostratigraphy method^20^ assumes Dollo's parsimony (that is, it is more likely that a gene observed in two distant clades was present in the common ancestor and multiple independent gains are not possible). This provides an entry point for testing evolutionary hypotheses related to the age of genes and to quantify how much gene-level innovation has occurred along each phylogenetic branch. Old genes are classified in low phylostrata (present in distant species, PS1–PS7) and young genes are classified in higher phylostrata (for example, genus- or species-specific genes, PS8–PS9). The resolution of each phylostratum strictly depends on the availability of reliable outgroups (the availability of reliable genomic outgroups is relatively low in Chlorophyte algae). The phylogenetic classes were defined from those in each NCBI Taxonomy entry for *Chlamydomonas*, *Gonium* and *Volvox*, resulting in nine expected phylostrata for each species. All proteins were subjected to a BLASTP search with an *E*-value threshold of 0.001 against the NCBI nr database. Placement in phylostrata was derived from the taxonomic information of these hits for each protein, using the most distant hit, and following Dollo's parsimony.

### Phylogenetic analyses

Unless otherwise stated, all phylogenetic analyses were performed using a custom pipeline of SATe version 2.2.7 (ref. 69) coupled with RAxML version 8 (ref. 62). Full gene protein sequences were passed to SATe using a FASTTREE tree estimation with a RAxML search after tree formation with a maximum limit of 10 iterations and the ‘longest' decomposition strategy. Bootstraps were made on the SATe output alignment and tree using RAxML with automatic model selection, a rapid hill climbing algorithm (−f d) and 100 bootstrap partitions. Bipartition information (−f a) was obtained using the SATe output tree and RAxML bootstraps.

### *Chlamydomonas* strains culture conditions

Wild-type *Chlamydomonas reinhardtii* 6145 and 21gr, and *HA-CrRB* (*HA-MAT3::mat3–4*, here referred to as *HA-CrRB::rb*), *mat3–4* (here referred to as *rb*), and *dp1* have been previously described^9^,^10^,^11^. Briefly, wild-type strains 6145 (MT−) and 21gr (MT+) are mating pairs that have been back crossed to eliminate the *y1* mutation in 6145 (ref. 10). The *RB* knockout strain has been previously characterized as a null allele, and the knockout mutation is the *rb* allele^9^,^11^. The *rb* mutation can be complemented by a amino (N)-terminally tagged version of the gene that behaves identical to wild type. Previously, a knockout mutation in the *Chlamydomonas DP1* gene, *dp1*, was identified and characterized^10^,^11^. All the strains were maintained on TAP plates. For phenotype analysis, the strains were grown in high salt media (HSM) synchronously under 14 h of 150 μE of light, samples were fixed hourly and examined by light microscopy^10^,^11^.

### Cloning of *Gonium pectorale RB* and transformation into *rb*

A 3X haemagluttin (HA) tagged copy of the *Gonium pectorale RB* gene was cloned using InFusion Cloning (Clontech) to be driven by the *Chlamydomonas RB* promoter and terminator that includes a *AphVIII* selectable marker for *Chlamydomonas* transformation (Fig. 4, (ref. 11)). *Gonium pectorale* genomic DNA from K4F3 was used as a template and the genomic region of *RB* was amplified without its ATG start codon using the primers 5′-CAGATTACGCTACTAGATCTGCCGAAGCTGAACGTTTTACTGCG-3′, and 5′-CTCCGGCCGCGGTGCCTAATTTGCGCCGTACCGCCGGA-3′. These primers overlap with the 3X HA tag and 3′ terminator from the previously created HA-CrRB transformation clone that complements the *rb* mutation^11^. The HA-CrRB plasmid was amplified by inverse PCR with 5′-TCTAGTAGCGTAATCTGGAACGTCATATGGATAGG-3′ and 5′-GCACCGCGGCCGGAGGT-3′ primers. PCR products were gel purified with a QiaQuick gel extraction kit (Qiagen). Purified PCR fragments were fused by InFusion (Clontech) cloning based on overlaps in the amplified sequences and transformed into chemically competent DH5-apha cells, after which the clone was confirmed by sequencing.

### Transformation of *Chlamydomonas reinhardtii*

The *rb* strain was transformed with glass beads^11^, with the *HA-GpRB* clone (above) and as a control with *HA-CrRB* and pSI103 (*AphVIII* selectable marker only) and selected on TAP plates supplemented with 20 μg ml^−1^ paromycin^11^. Candidate strains were screened by growth morphology^10^,^11^, and then screened for expression by immunoblotting with an anti-HA antibody (Roche 3F10, high affinity^11^). Four independent strains expressing the *HA-GpRB*, and five independent strains expressing *HA-CrRB* were created. Control complementation of the *rb* mutation with *HA-CrRB* occurred at rates similar to previous results^11^. The presence of the *rb* mutation was confirmed by replica plating on TAP plates supplemented with 10 μg ml^−1^ emetine^9^,^11^.

### Genetic analysis of *HA-GpRB*-expressing strains

Two lines expressing *HA-GpRB* were crossed to a *dp1* null mutation^10^. Because both the *HA-GpRB* and *dp1* mutations are linked to *AphVIII,* single tetrads were dissected. *HA-GpRB* was genotyped with primers in the 3XHA tag 5′-AGTGCTAACAGCATGTCTAGTTAC-3′, and in the 5′ portion of *GpRB* 5′-TGCGAACAACCGCTGCAGACCTTC-3′. The *dp1* mutation was genotyped as previously described^10^.

### Immunoblotting *HA-GpRB* and *HA-CrRB* strains complementing *rb*

Whole-cell lysates from strains were prepared, separated and immunoblotted^11^. Briefly, the anti-HA antibody used for detection of *HA-GpRB* and *HA-CrRB* was an anti-HA high affinity monoclonal antibody (clone 3F10, Roche) and anti-alpha-tubulin monoclonal antibody (Sigma), as previously described^11^. The expression levels of *RB* in *HA-CrRB* strains have been previously shown to be similar to wild-type *Chlamydomonas* expression levels^11^. The expression levels of *RB* in *HA-GpRB* are similar, if not slightly below, the expression levels of *HA-CrRB*, suggesting that overexpression of *RB* is not causing the observed colonial phenotype, but rather modification to the *Gonium RB* gene.

### Measurement of cell or colony size distribution

The size of cells and groups of cells was measured with a Moxi Z automated cell sizer/counter using type ‘S' cassettes (ORFLO Technologies). Sizing is based on the Coulter principle used previously with *Chlamydomonas reinhardtii*^10^,^11^.

## Additional information

**Accession codes:** This Whole Genome Shotgun project has been deposited at DDBJ/ENA/GenBank under the accession LSYV00000000. The version described in this paper is version LSYV01000000. Data are also available via JGI Phytozome.

**How to cite this article:** Hanschen, E. R. *et al.* The *Gonium pectorale* genome demonstrates co-option of cell cycle regulation during the evolution of multicellularity. *Nat. Commun.* 7:11370 doi: 10.1038/ncomms11370 (2016).

## Supplementary Material

Supplementary Information Supplementary Figures 1-29, Supplementary Tables 1-14, Supplementary Methods and Supplementary References

Supplementary Data 1 Significantly over (1) and under (-1) represented Pfam domains in multicellular algae (Gonium and Volvox) compared to unicellular green algae.

Supplementary Data 2 Matrix metalloprotease genes in Chlamydomonas, Gonium and Volvox.

Supplementary Data 3 Pherophorin genes in Chlamydomonas, Gonium and Volvox.

Supplementary Data 4 Proteins involved in processes associated with developmental complexity in Chlamydomonas, Gonium, and Volvox.

## Figures and Tables

**Table 1:** Summary statistics for genome level analyses for *Chlamydomonas*, *Gonium* and *Volvox*.

**Characteristic**	***Chlamydomonas*** **v5.3**	***Gonium***	***Volvox*** **v1**	***Volvox*** **v2**
Genome size (Mb)	111.1	148.8	137.8	131.1
Scaffold N50 (Mb)	7.78	1.27	1.49	2.6
Number of contigs/scaffolds	54	2,373	1,265	434
% G and C	64.1	64.5	56.0	56.1
Protein coding loci	17,737	17,984	15,669	14,971
Gene density (genes/Mb)	159.6	120.9	113.7	114.1
Introns/gene	7.46	6.50	6.78	6.29
Average intron length (bp)	279.17	349.83	496.67	399.50
% genes w/introns	92.4	92.6	82.8	84.0
