# Supplementary material for: The Gonium pectorale genome demonstrates co-option of cell cycle regulation during the evolution of multicellularity
Source: Nat Commun. 2016 Apr 22;7:11370. doi: 10.1038/ncomms11370 (PMC4844696; doi:10.1038/ncomms11370)
Supplement: Supplementary Information — Supplementary Figures 1-29, Supplementary Tables 1-14, Supplementary Methods and Supplementary References [file ncomms11370-s1.pdf]

Supplementary Figures

Supplementary Figure 1. Detailed rooted phylogenetic tree of the volvocine algae. Adapted from previous analyses[1–3]. *Chlamydomonas* is denoted in green, *Gonium* is denoted in blue, and *Volvox* is denoted in black. Other species are in gray. Filled circles represent obligate somatic differentiation, open circles represent facultative somatic differentiation, and dots represent the absence of somatic differentiation. Numbers represent the maximum cell number[2,4].

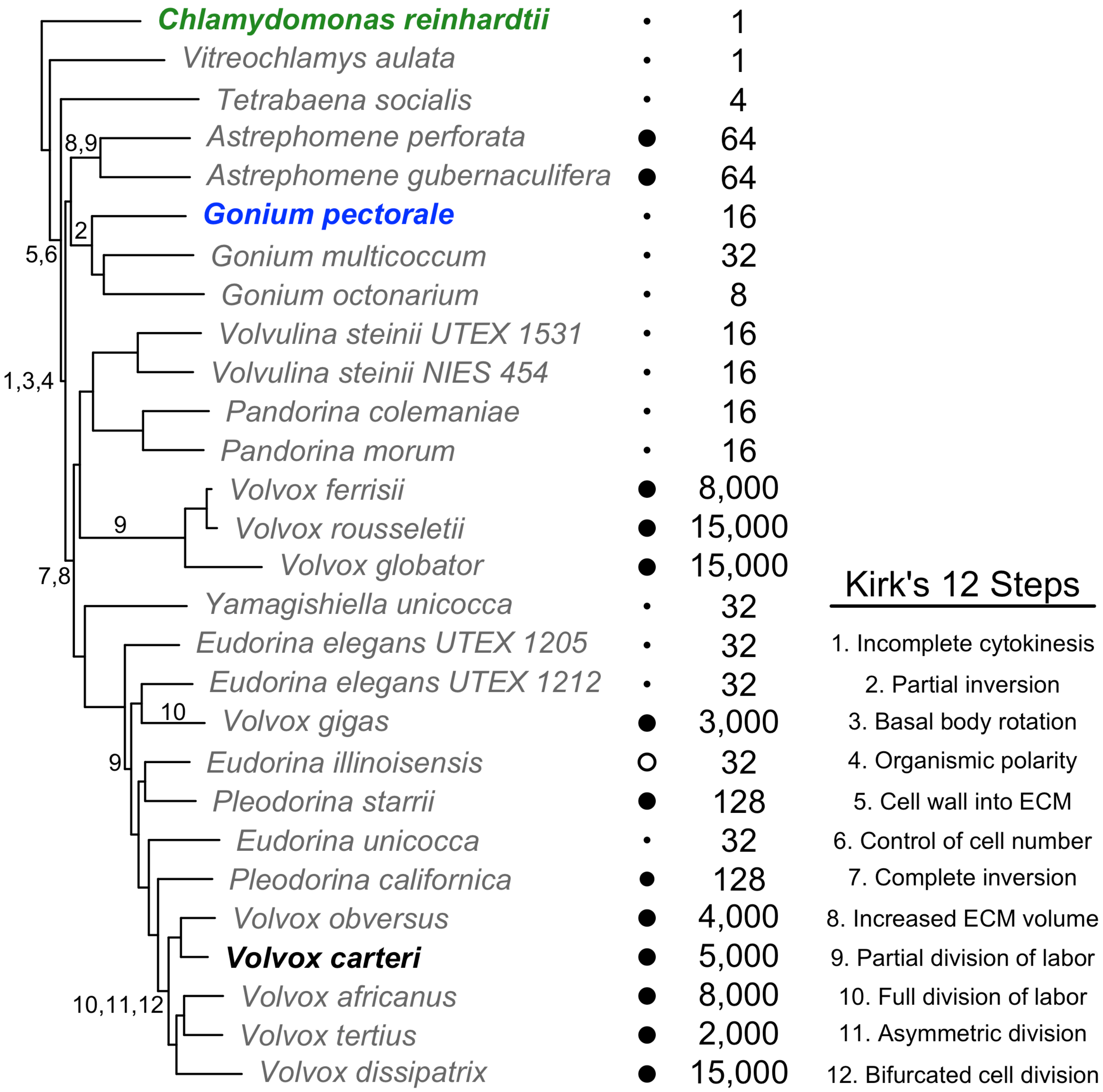
Chlamydomonas reinhardtii
1
Vitreochlamys aulata
1
Tetrabaena socialis
4
Astrephomene perforata
64
Astrephomene gubernaculifera
64
Gonium pectorale
16
Gonium multicoccum
32
Gonium octonarium
8
Volvulina steinii UTEX 1531
16
Volvulina steinii NIES 454
16
Pandorina colemaniae
16
Pandorina morum
16
Volvox ferrisii
8,000
Volvox rousseletii
15,000
Volvox globator
15,000
Yamagishiella unicocca
32
Eudorina elegans UTEX 1205
32
Eudorina elegans UTEX 1212
32
Volvox gigas
3,000
Eudorina illinoisensis
32
Pleodorina starrii
128
Eudorina unicocca
32
Pleodorina californica
128
Volvox obversus
4,000
Volvox carteri
5,000
Volvox africanus
8,000
Volvox tertius
2,000
Volvox dissipatrix
15,000
8,9
2
5,6
1,3,4
9
7,8
10
9
10,11,12
Kirk's 12 Steps
1. Incomplete cytokinesis
2. Partial inversion
3. Basal body rotation
4. Organismic polarity
5. Cell wall into ECM
6. Control of cell number
7. Complete inversion
8. Increased ECM volume
9. Partial division of labor
10. Full division of labor
11. Asymmetric division
12. Bifurcated cell division

Supplementary Figure 2. Asexual (vegetative) and sexual life cycles of *Chlamydomonas reinhardtii*, *Gonium pectorale*, and *Volvox carteri*. Adult *Chlamydomonas* undergo multiple rounds of cell division (multiple fission) and daughter cells hatch from the cell wall of the adult. When cells develop in the absence of nitrogen, cells differentiation into gametes (*plus* and *minus* mating types) which mate to form a diploid zygotic spore. When environmental conditions improve, the spore germinates, yielding four mitotic products. The life cycle in *Gonium* is similar, though forming colonies of approximately 8 cells (3 rounds of division). In *Volvox*, germ cells within an adult spheroid undergo multiple rounds of division then inversion (a post-cleavage embryo develops with flagella pointed towards the center of the embryo, which must invert to ensure flagella pointing towards the environment). The resulting juveniles hatch from the adult spheroid. When juveniles develop in the presence of a proteinacious hormone, sex inducer, reproductive cells differentiation as eggs (in mating type female, MTF) or sperm packets (in mating type male, MTM). Sperm packets penetrate the female spheroid to fertilize the eggs, forming a diploid zygotic spore. When environmental conditions improve, the zygote undergoes meiosis and a single meiotic product germinates and develops as a small asexual spheroid. Note that all three species are heterothallic, meaning that the male and female sexes are separate strains.

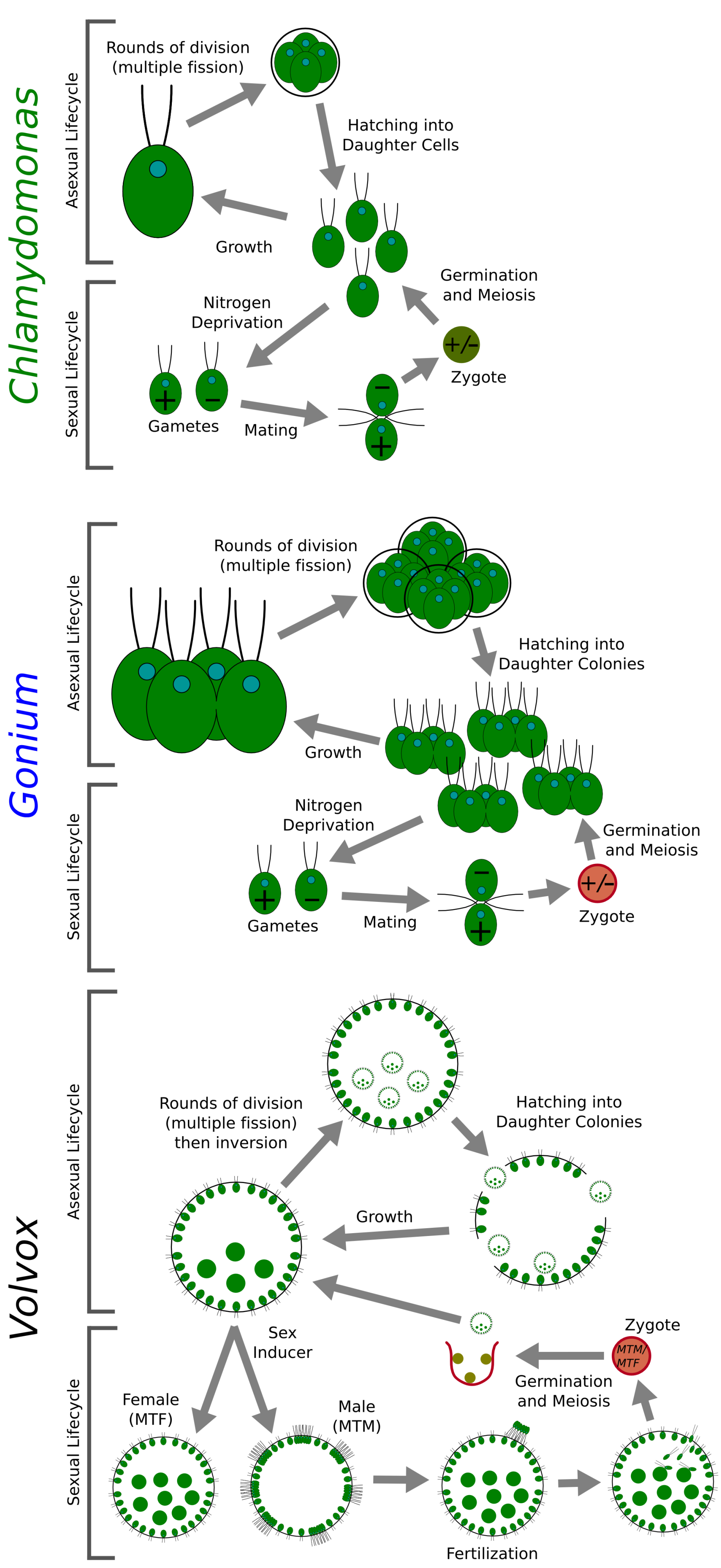
Chlamydomonas
Asexual Lifecycle
Rounds of division
(multiple fission)
Hatching into
Daughter Cells
Growth
Sexual Lifecycle
Nitrogen
Deprivation
Germination
and Meiosis
+/−
Zygote
Gametes
Mating
Gonium
Asexual Lifecycle
Rounds of division
(multiple fission)
Hatching into
Daughter Colonies
Growth
Sexual Lifecycle
Nitrogen
Deprivation
Germination
and Meiosis
+/−
Zygote
Gametes
Mating
Volvox
Asexual Lifecycle
Rounds of division
(multiple fission)
then inversion
Hatching into
Daughter Colonies
Growth
Sexual Lifecycle
Sex
Inducer
Zygote
MTM/
MTF
Germination
and Meiosis
Female
(MTF)
Male
(MTM)
Fertilization

Supplementary Figure 3. Heatmap of transcription associated proteins including *Chlamydomonas reinhardtii* version 5.3 and *Volvox carteri* version 1 genomes. Transcription associated protein abundance has been normalized by the total number of genes in each species and relative values to the maximum were calculated (yellow represents high transcription factor representation, green represents low transcription factor representation). White represents the absence of that transcription associated protein, species (columns) and transcription associated proteins (rows) are hierarchically clustered.

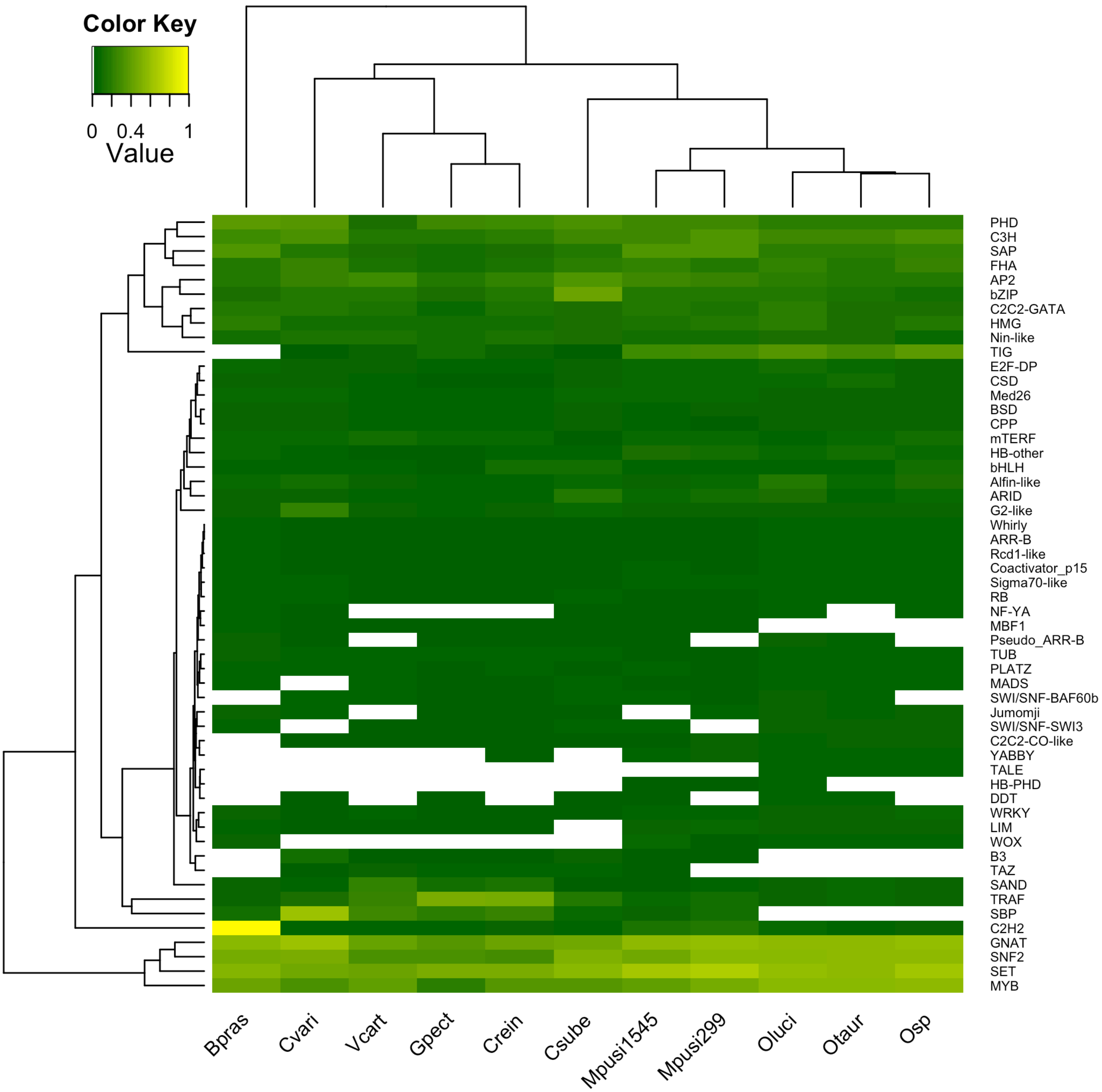

Color Key
0 0.4 1
Value
PHD
C3H
SAP
FHA
AP2
bZIP
C2C2-GATA
HMG
Nin-like
TIG
E2F-DP
CSD
Med26
BSD
CPP
mTERF
HB-other
bHLH
Alfin-like
ARID
G2-like
Whirly
ARR-B
Rcd1-like
Coactivator_p15
Sigma70-like
RB
NF-YA
MBF1
Pseudo_ARR-B
TUB
PLATZ
MADS
SWI/SNF-BAF60b
Jumomji
SWI/SNF-SWI3
C2C2-CO-like
YABBY
TALE
HB-PHD
DDT
WRKY
LIM
WOX
B3
TAZ
SAND
TRAF
SBP
C2H2
GNAT
SNF2
SET
MYB
Bpras
Cvari
Vcart
Gpect
Crein
Csube
Mpusi1545
Mpusi299
Oluci
Otaur
Osp

Supplementary Figure 4. Heatmap of significantly over- and under-represented Pfam A domains in multicellular species. Includes *Chlamydomonas reinhardtii* version 5.3 and *Volvox carteri* version 1 genomes. Abundance of Pfam A domains has been normalized by the total number of genes in each species and relative values to the maximum were calculated (yellow represents high domain representation, green represents low domain representation). Pfam domains that do not have a significant over- or under-representation in multicellular *Gonium* and *Volvox* using a G test of independence with William's correction (α=0.05) have been removed. 523 significantly over- (129) or under-represented (394) Pfam A domains were found. White represents the absence of that Pfam domain, species (columns) and Pfam domains (rows) are hierarchically clustered.

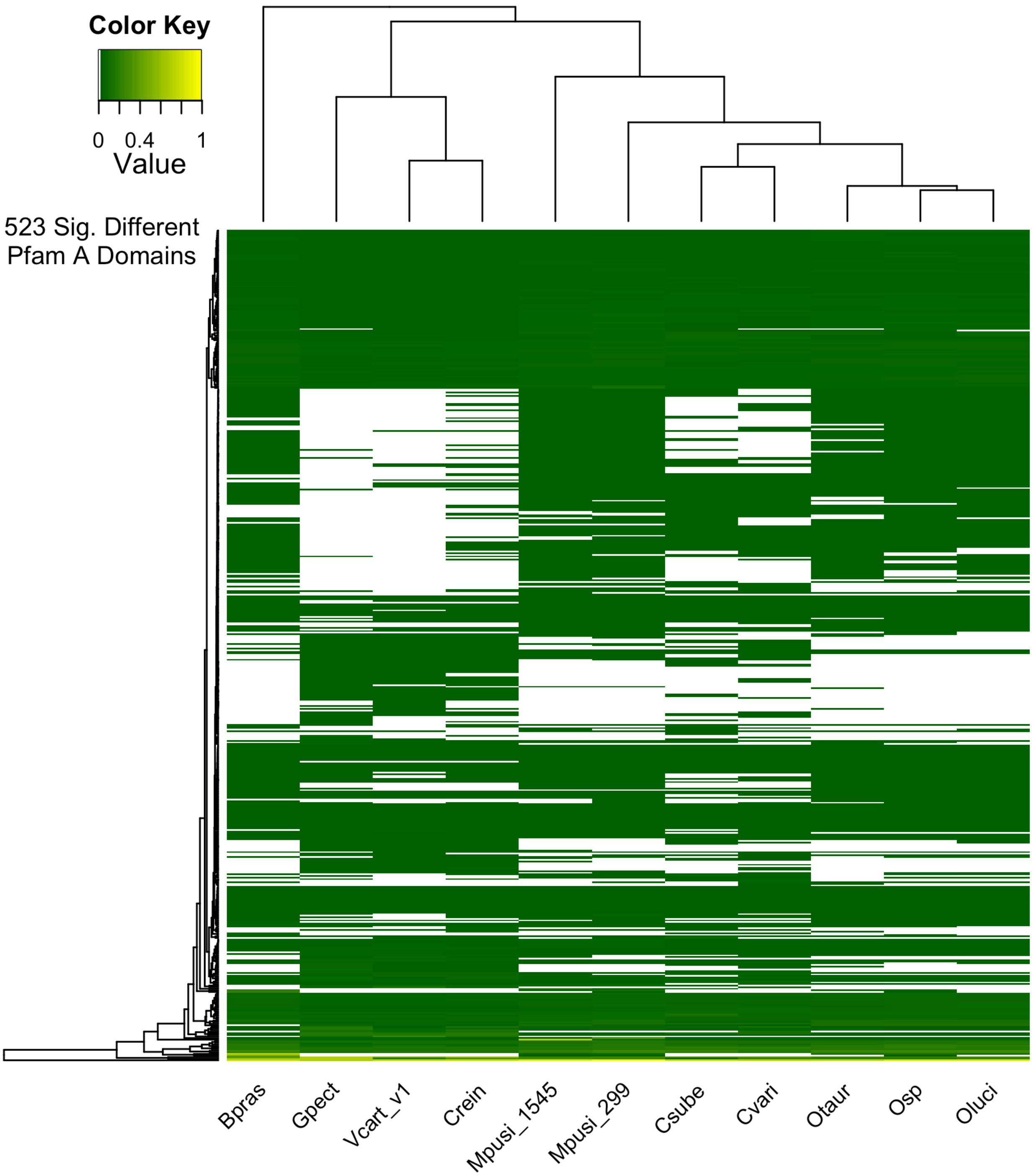

Color Key
0
0.4
1
Value
523 Sig. Different
Pfam A Domains
Bpras
Gpect
Vcart_v1
Crein
Mpusi_1545
Mpusi_299
Csube
Cvari
Otaur
Osp
Oluci

Supplementary Figure 5. Multiple sequence alignment of MAT3/RB. Alignment of *MAT3/RB* for *Chlamydomonas reinhardtii* (C.r.), *Gonium pectorale* (G.p.), *Volvox carteri* female (V.c. female), and *Volvox carteri* male (V.c. male). Solid inverted triangles denote conserved cyclin dependent kinase phosphorylation sites. Open inverted triangles denote degenerate or species specific cyclin dependent kinase phosphorylation sites. Shaded bars beneath the alignment show conserved regions N-terminus (N1-N3, gray), RB-A domain (black), Linker region (L, gray; as previously defined[5]), RB-B domain (black), and C-terminus (C1 and C4, gray). Black and gray shading within the alignment indicates conservation in all four proteins.

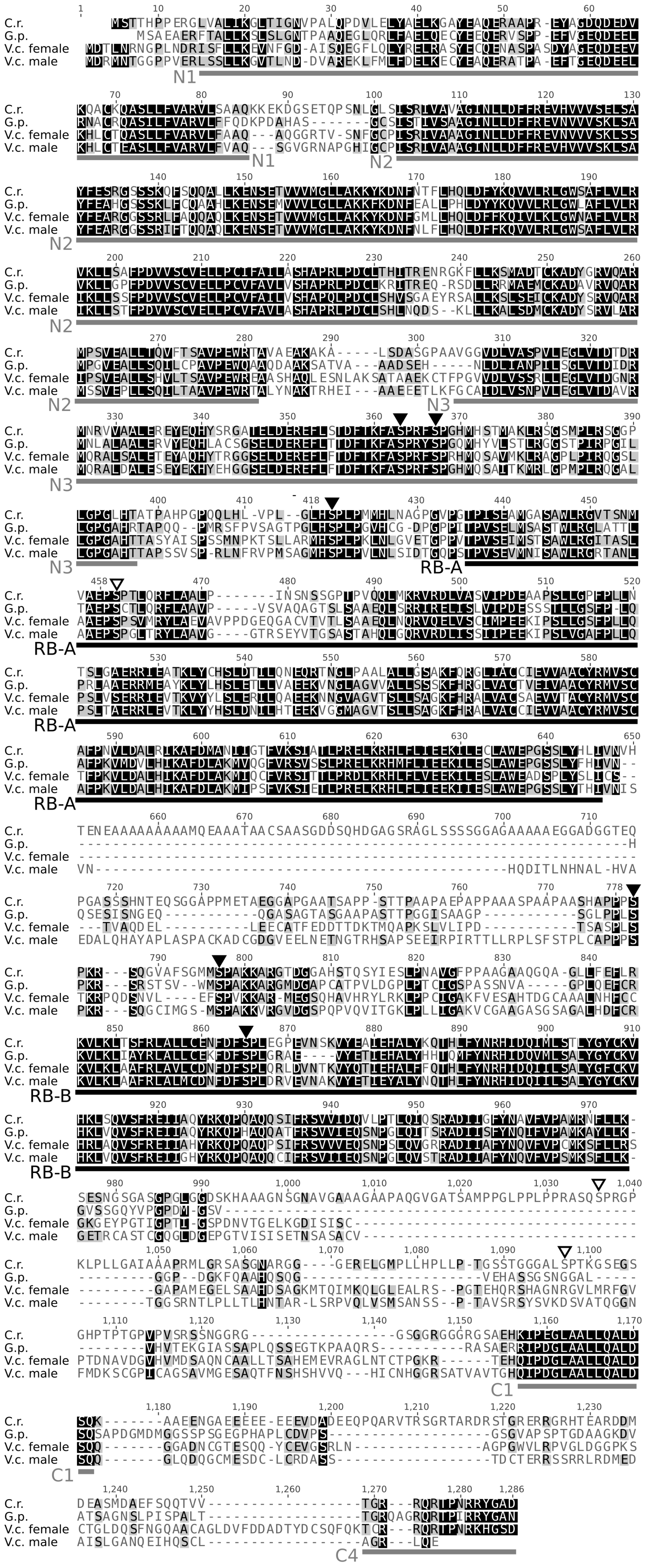
C.r.
G.p.
V.c. female
V.c. male
N1
N2
N3
RB-A
RB-B
C1
C4

Supplementary Figure 6. Protein-protein similarity plots for MAT3/RB proteins in *Chlamydomonas*, *Gonium*, *Volvox* male, and *Volvox* female. All pairwise comparisons are shown. A sliding window of 20 amino acids was used for all panels. N-terminal conservation (N), RB-A domain, RB-B domain, and C-terminal conservation (C) are indicated by horizontal bars.

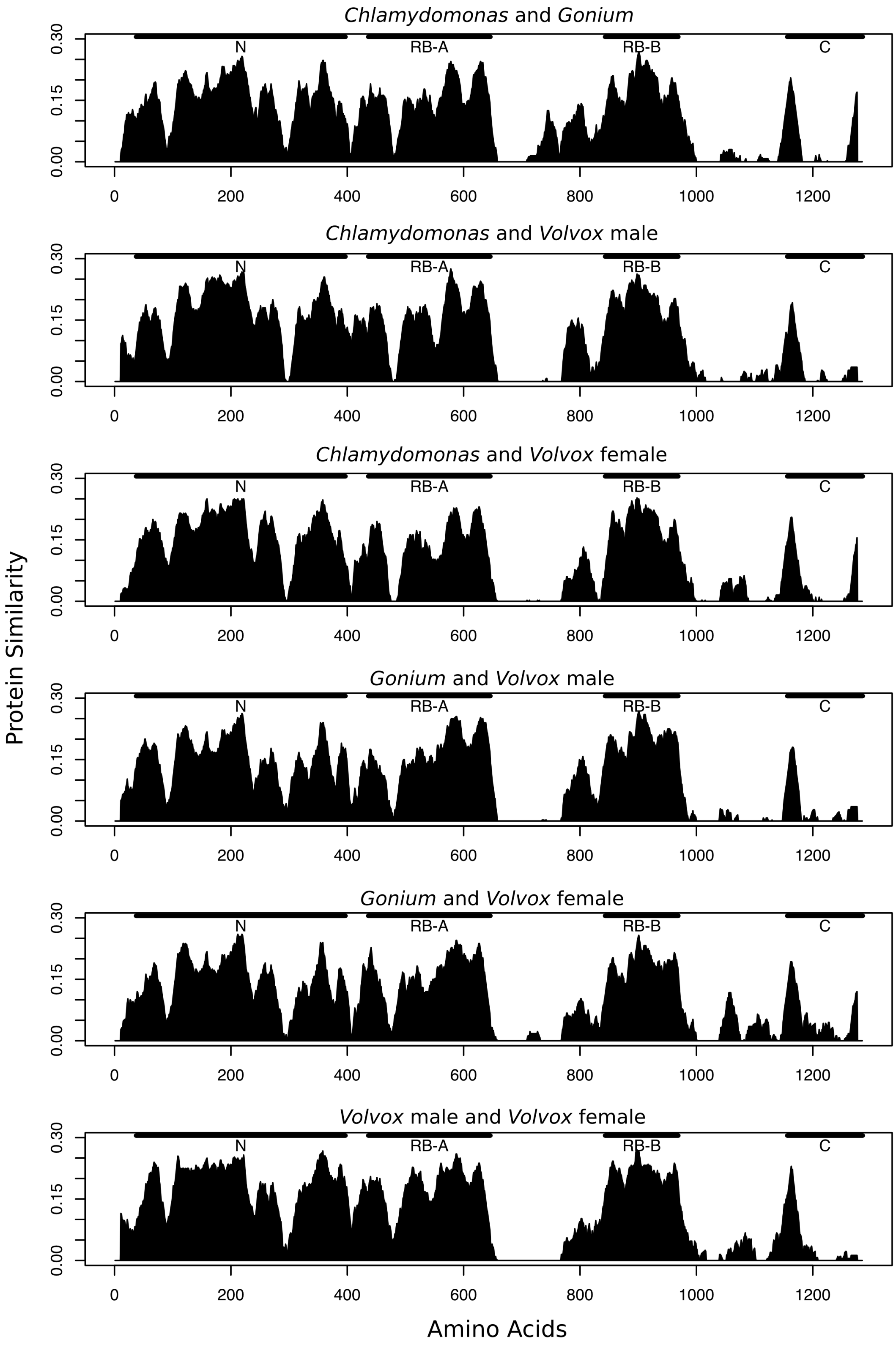
Chlamydomonas and Gonium
Chlamydomonas and Volvox male
Chlamydomonas and Volvox female
Gonium and Volvox male
Gonium and Volvox female
Volvox male and Volvox female
N
RB-A
RB-B
C
0.00
0.15
0.30
0
200
400
600
800
1000
1200
Protein Similarity
Amino Acids

Supplementary Figure 7. Differences between protein-protein similarity plots. Similarity data from Supplementary Figure 6 is subtracted, resulting in comparative protein similarity plots. How *Gonium* is more similar to all pairwise comparisons of *Chlamydomonas*, *Volvox* male and *Volvox* female are shown. A sliding window of 20 amino acids was used for all panels. N-terminal conservation (N), RB-A domain, RB-B domain, and C-terminal conservation (C) are indicated by horizontal bars.

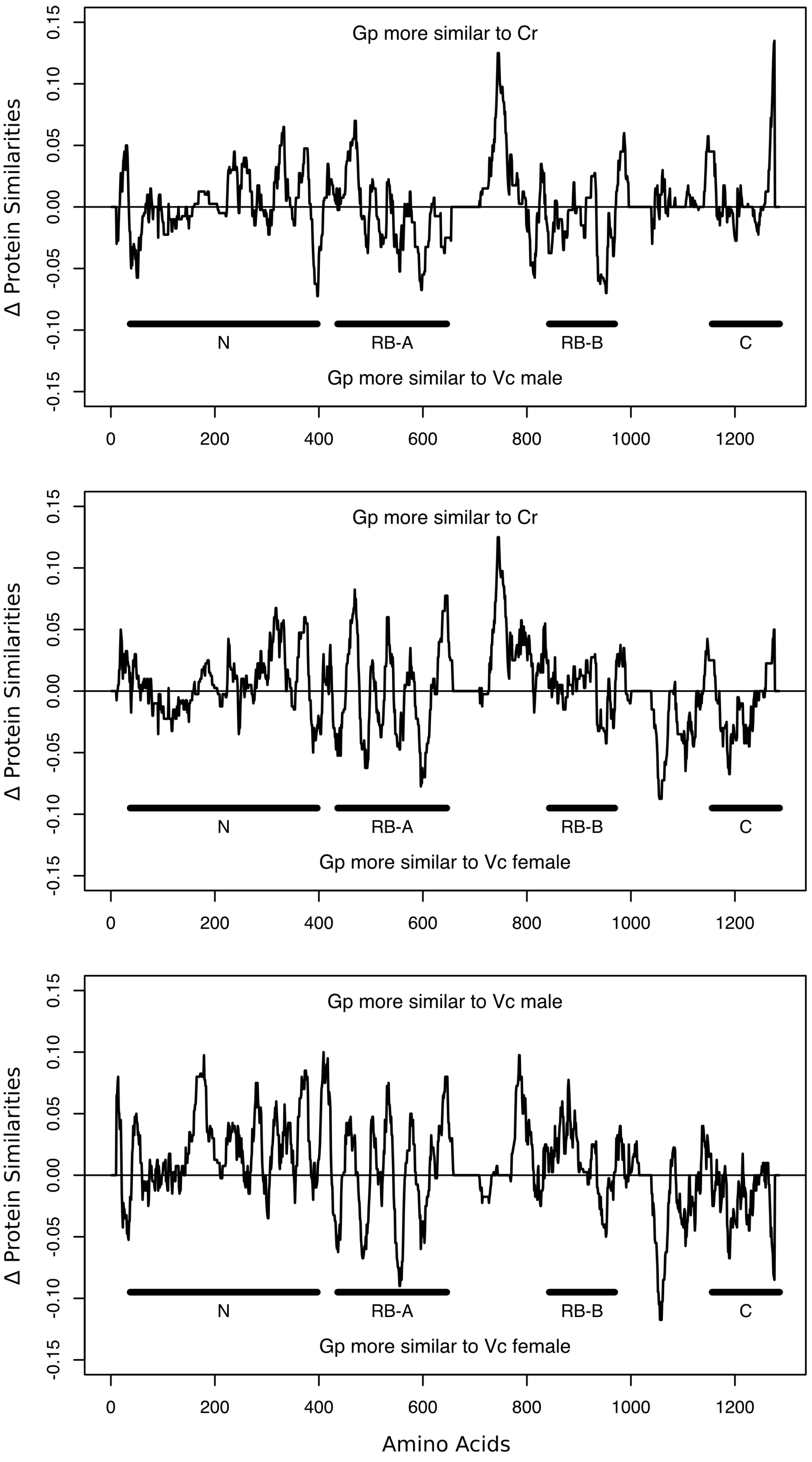
Gp more similar to Cr
N
RB-A
RB-B
C
Gp more similar to Vc male
Δ Protein Similarities
Gp more similar to Cr
N
RB-A
RB-B
C
Gp more similar to Vc female
Δ Protein Similarities
Gp more similar to Vc male
N
RB-A
RB-B
C
Gp more similar to Vc female
Δ Protein Similarities
Amino Acids

Supplementary Figure 8. Box-whisker distribution of interspecies pairwise dN (red), dS (blue), and dN/dS (purple) values for cell cycle regulators including *Chlamydomonas*, *Gonium*, and *Volvox*.

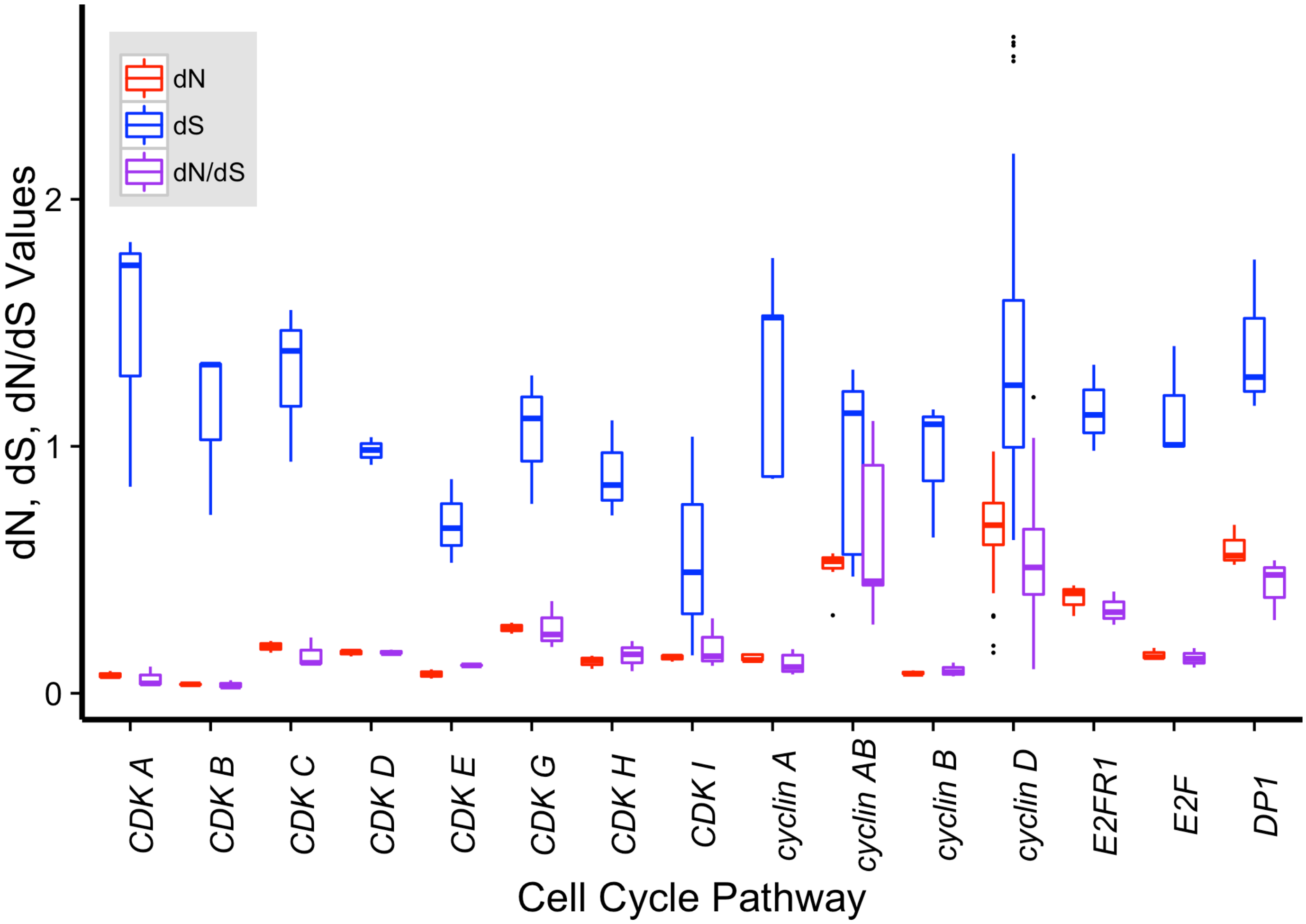

dN
dS
dN/dS
dN, dS, dN/dS Values
0
1
2
CDK A
CDK B
CDK C
CDK D
CDK E
CDK G
CDK H
CDK I
cyclin A
cyclin AB
cyclin B
cyclin D
E2FR1
E2F
DP1
Cell Cycle Pathway

Supplementary Figure 9. Histograms of size distribution for assembled contigs (or scaffolds when scaffolding was performed) for *Chlamydomonas* version 5.3 (green), *Gonium* (blue), *Volvox* version 1, and *Volvox* version 2 (black). A consistent bin number (100) was used and a 1/log(Contig Size) applied to visualize the diverse contig size, using the natural logarithm. On the X-axis, 0.06 represents approximately 17.3 Mb, 0.10 represents approximately 22 kB, and 0.14 represents approximately 1.27 kB.

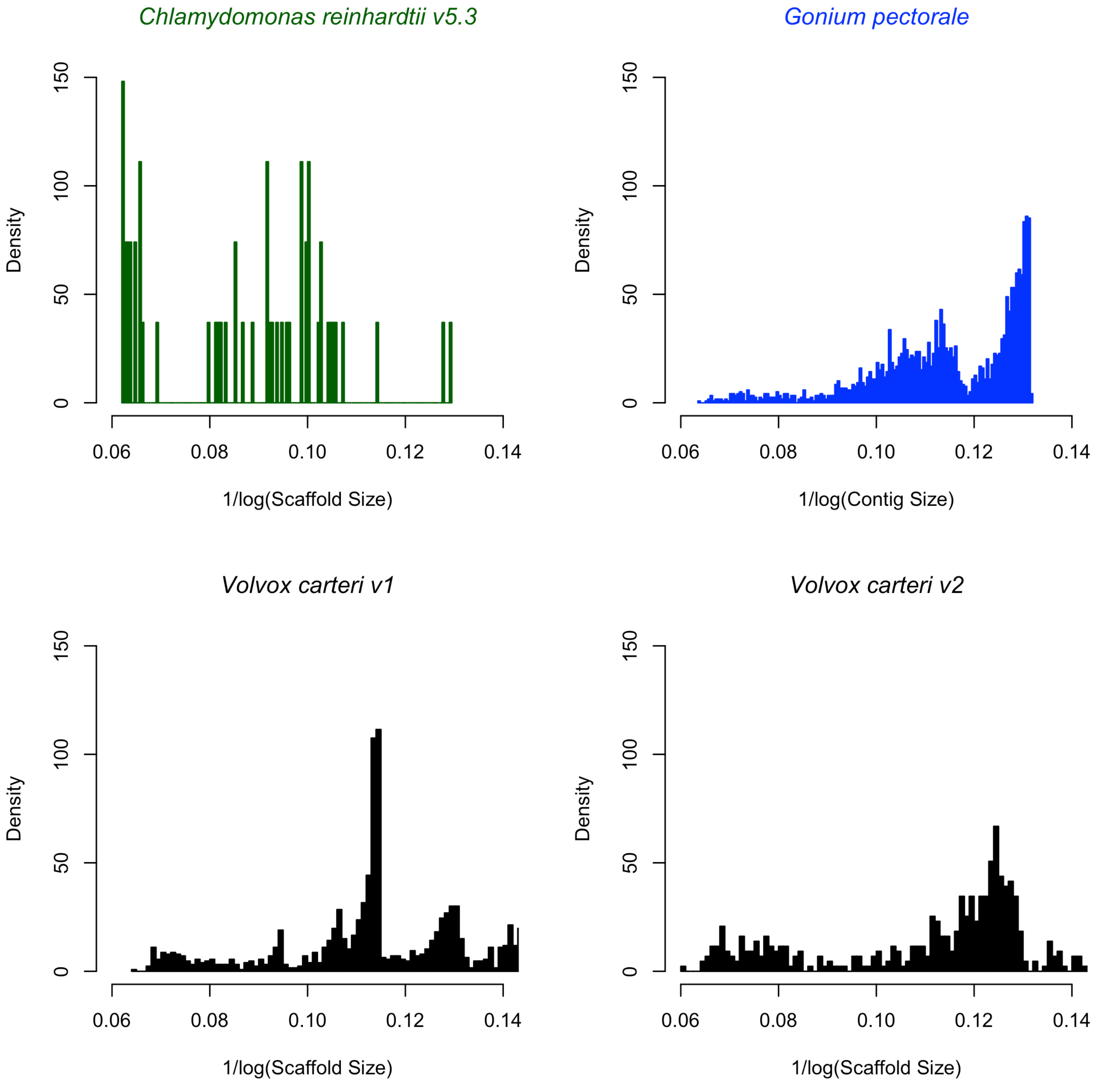

Chlamydomonas reinhardtii v5.3
Gonium pectorale
Density
0
50
100
150
0.06
0.08
0.10
0.12
0.14
1/log(Scaffold Size)
1/log(Contig Size)
Volvox carteri v1
Volvox carteri v2

Supplementary Figure 10. Unordered syntenic relationships between *Chlamydomonas* version 5.3 (green), *Gonium* (blue), and *Volvox* version 2 (black) genomes. Large blocks of synteny are evident. Contigs and scaffolds below 1MB have been removed and links are based on an initial OrthoMCL analysis using an inflation value of 1.5.

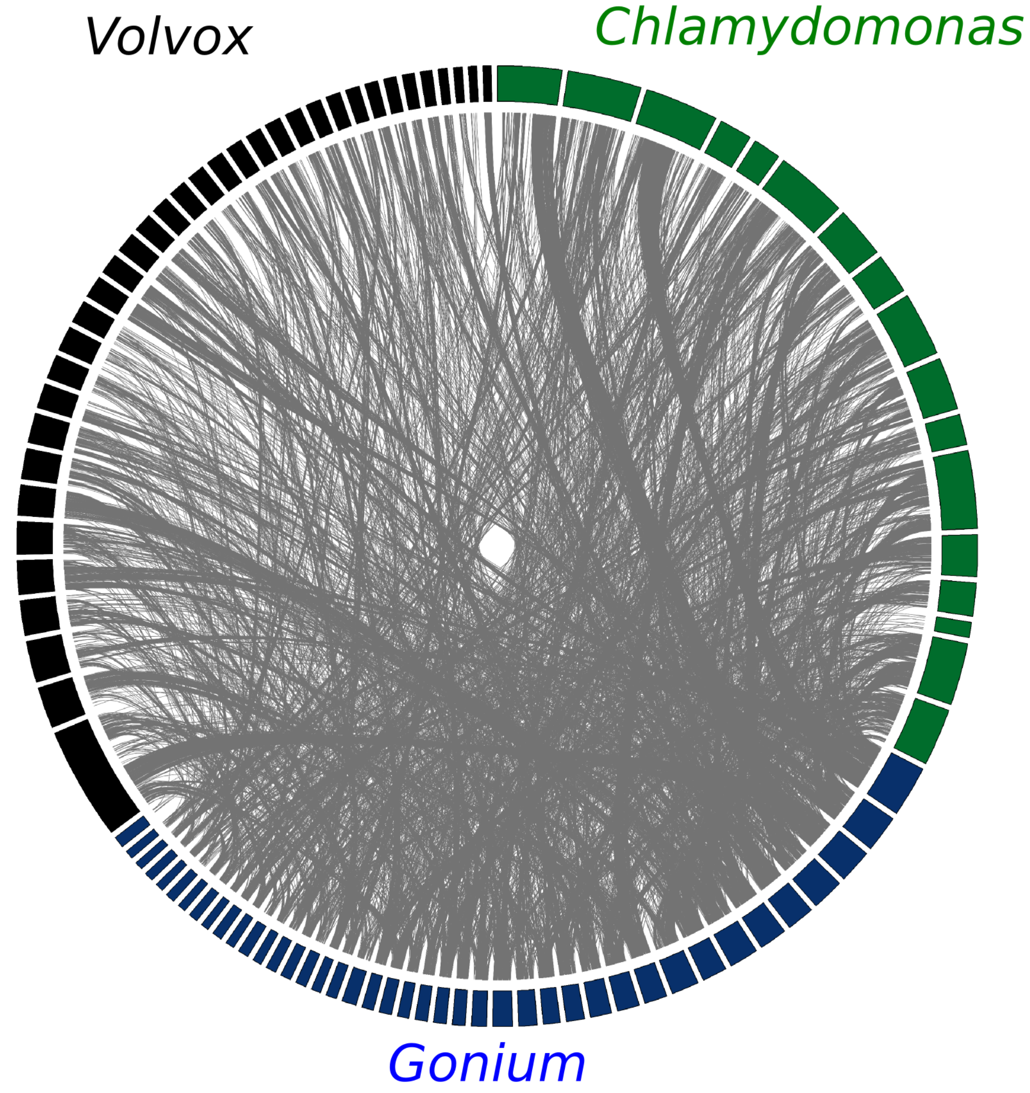
Volvox
Chlamydomonas
Gonium

Supplementary Figure 11. Unordered syntenic relationships between *Chlamydomonas* version 5.3 (green), *Gonium* (blue), and *Volvox* version 2 (black) genomes. In order to display large blocks of synteny, approximately 75% of links have been randomly removed. Contigs and scaffolds below 1MB have been removed and links are based on an initial OrthoMCL analysis using an inflation value of 1.5.

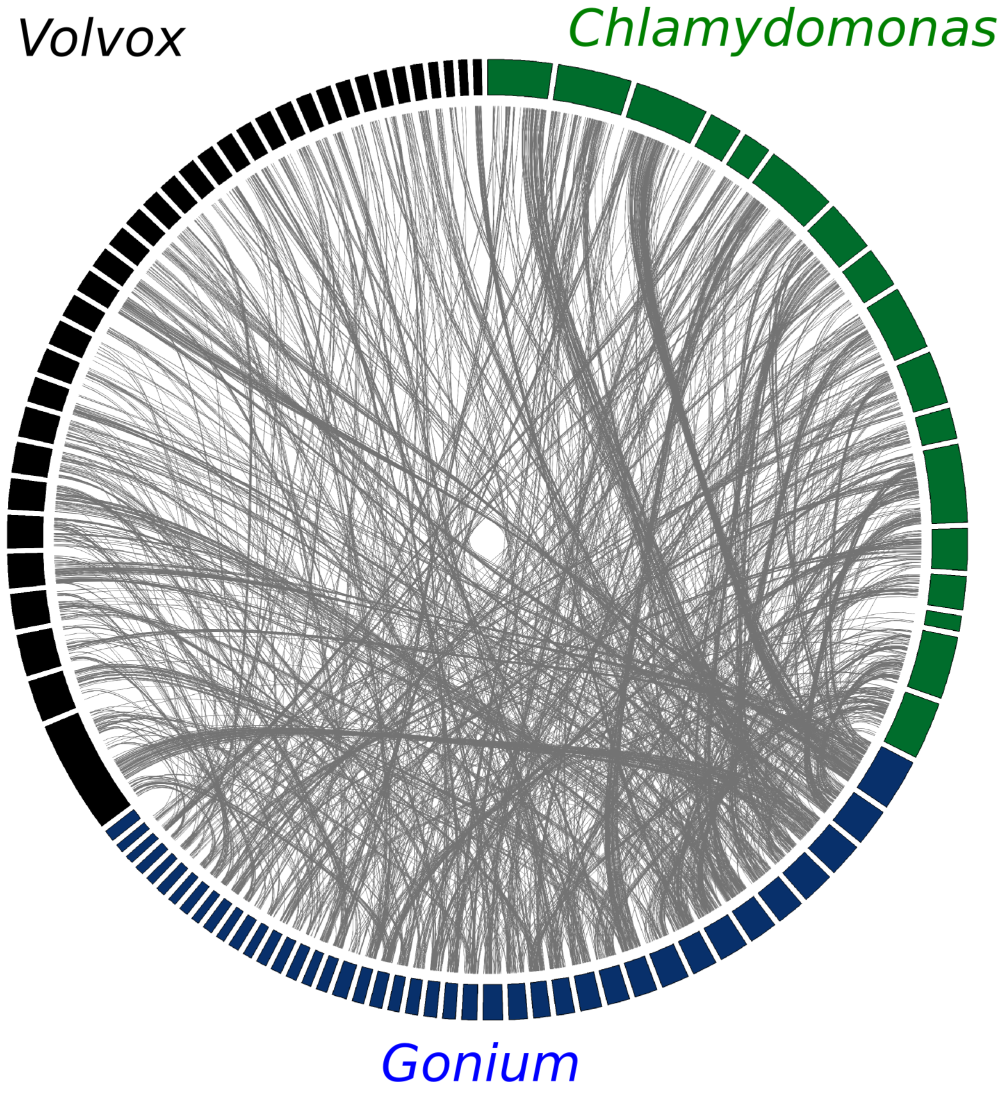
Volvox
Chlamydomonas
Gonium

Supplementary Figure 12. Heatmap of Pfam A domains including *Chlamydomonas reinhardtii* version 5.3 and *Volvox carteri* version 1 genomes. Abundance of Pfam A domains has been normalized by the total number of genes in each species and relative values to the maximum were calculated (yellow represents high domain representation, green represents low domain representation). White represents the absence of that Pfam domain, species (columns) and Pfam domains (rows) are hierarchically clustered.

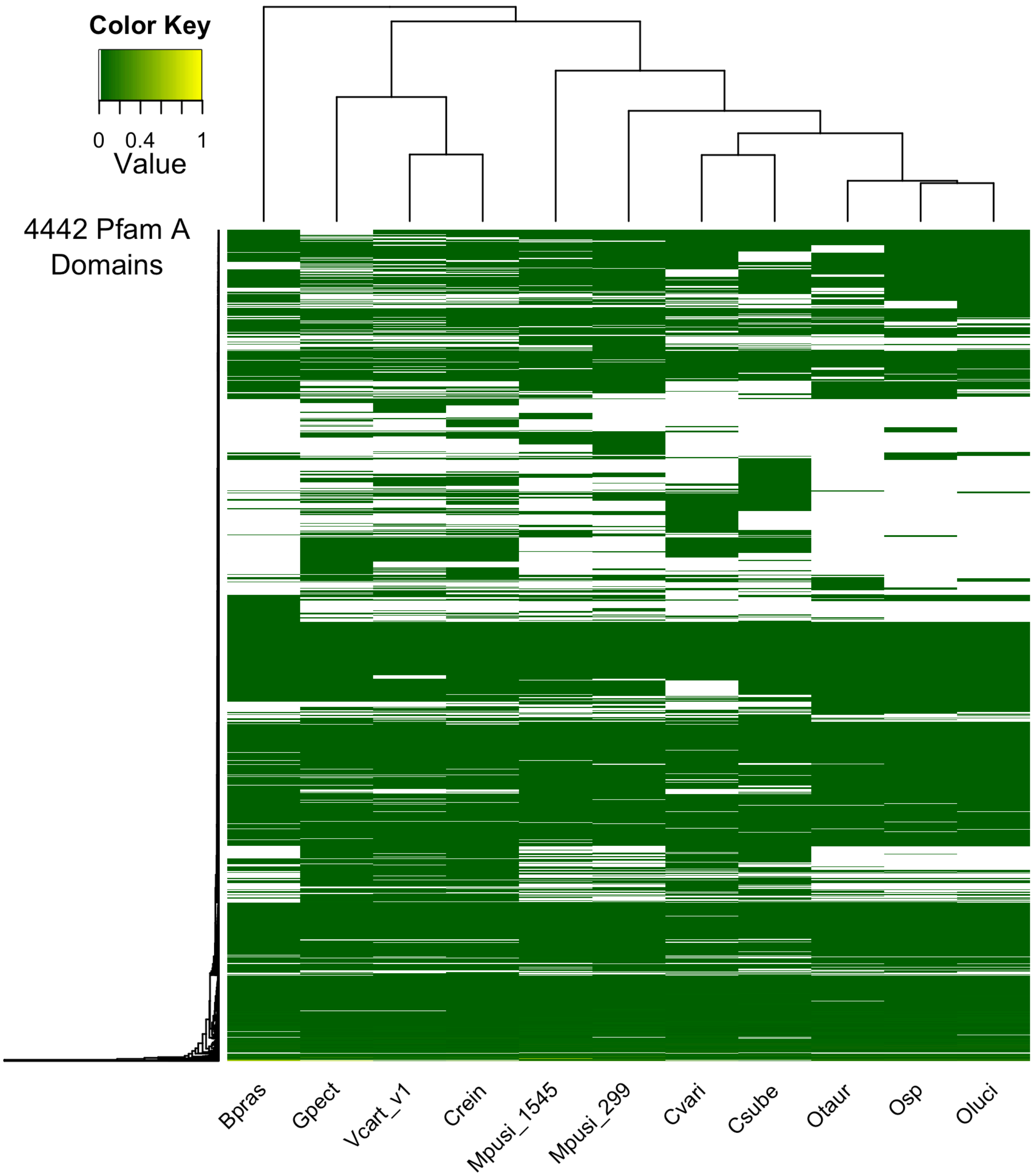
Color Key
0 0.4 1
Value
4442 Pfam A Domains
Bpras
Gpect
Vcart_v1
Crein
Mpusi_1545
Mpusi_299
Cvari
Csube
Otaur
Osp
Oluci

Supplementary Figure 13. Heatmap of Pfam A domains including *Chlamydomonas reinhardtii* version 5.3 and *Volvox carteri* version 2 genomes. Abundance of Pfam A domains has been normalized by the total number of genes in each species and relative values to the maximum were calculated (yellow represents high domain representation, green represents low domain representation). White represents the absence of that Pfam domain, species (columns) and Pfam domains (rows) are hierarchically clustered.

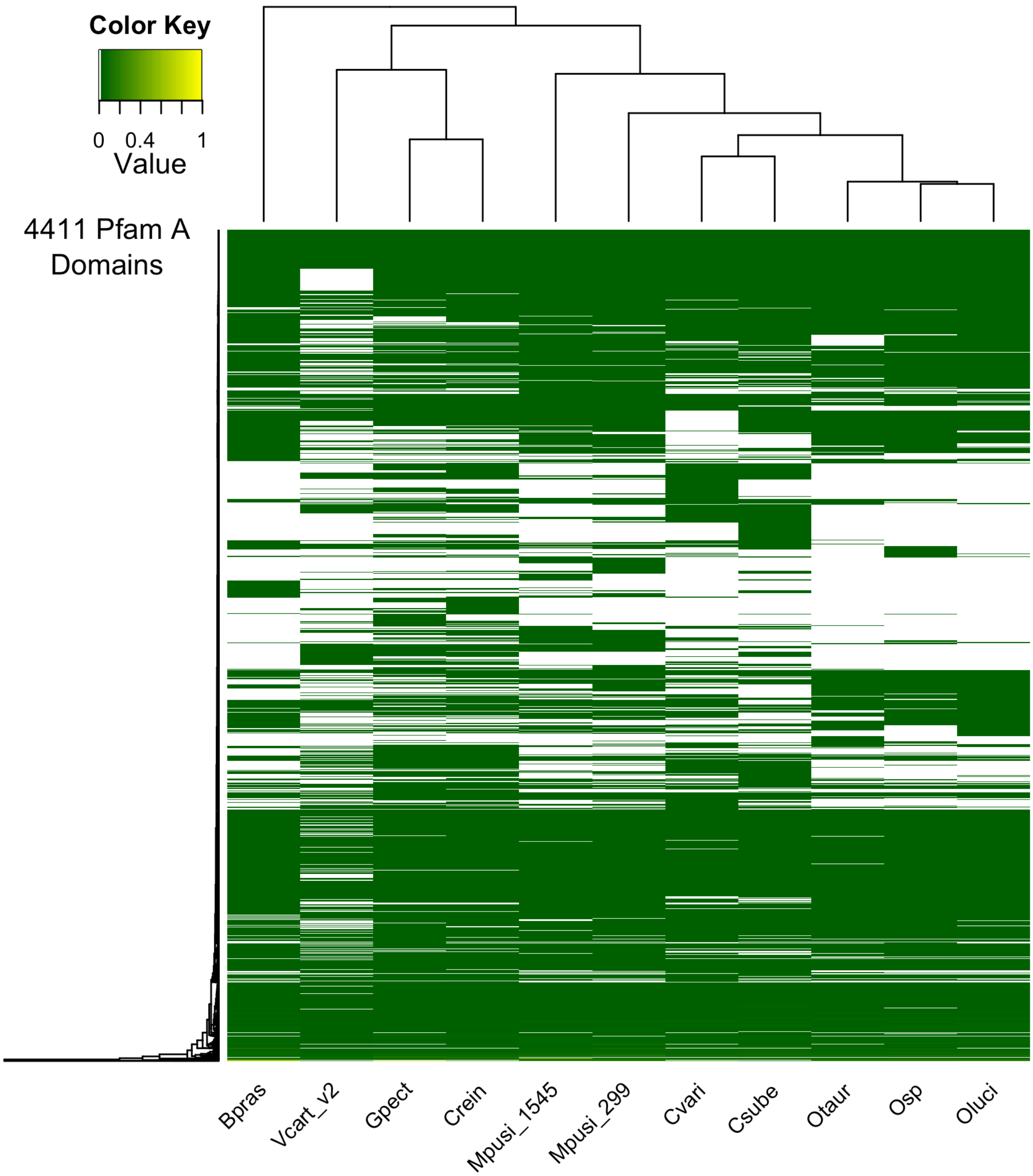
Color Key
0 0.4 1
Value
4411 Pfam A Domains
Bpras
Vcart_v2
Gpect
Crein
Mpusi_1545
Mpusi_299
Cvari
Csube
Otaur
Osp
Oluci

Supplementary Figure 14. Heatmap of Pfam B domains including *Chlamydomonas reinhardtii* version 5.3 and *Volvox carteri* version 1 genomes. Abundance of Pfam B domains has been normalized by the total number of genes in each species and relative values to the maximum were calculated (yellow represents high domain representation, green represents low domain representation). White represents the absence of that Pfam domain, species (columns) and Pfam domains (rows) are hierarchically clustered.

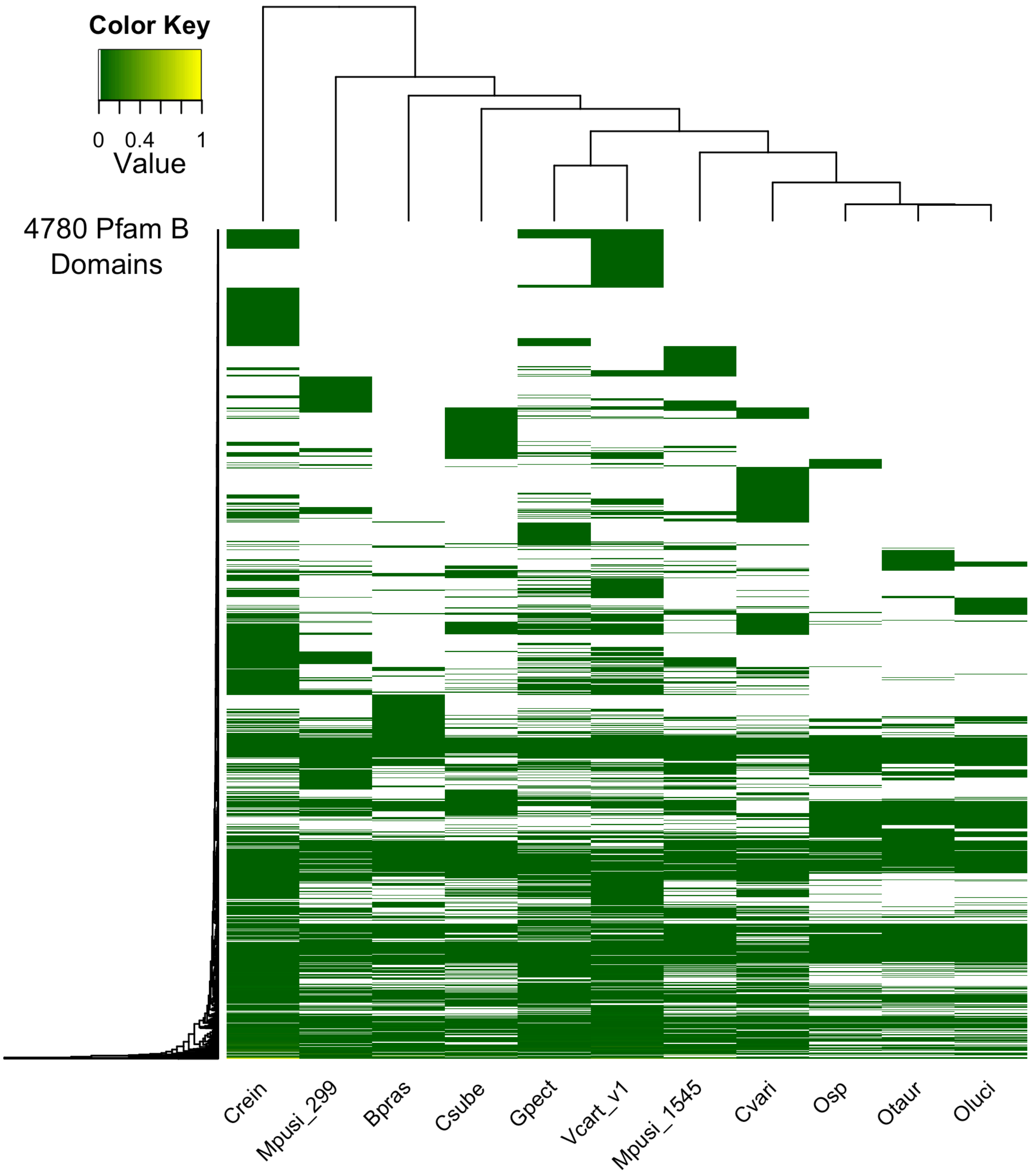
Color Key
0 0.4 1
Value
4780 Pfam B Domains
Crein
Mpusi_299
Bpras
Csube
Gpect
Vcart_v1
Mpusi_1545
Cvari
Osp
Otaur
Oluci

Supplementary Figure 15. Heatmap of Pfam B domains including *Chlamydomonas reinhardtii* version 5.3 and *Volvox carteri* version 2 genomes. Abundance of Pfam B domain has been normalized by the total number of genes in each species and relative values to the maximum were calculated (yellow represents high domain representation, green represents low domain representation). White represents the absence of that Pfam domain, species (columns) and Pfam domains (rows) are hierarchically clustered.

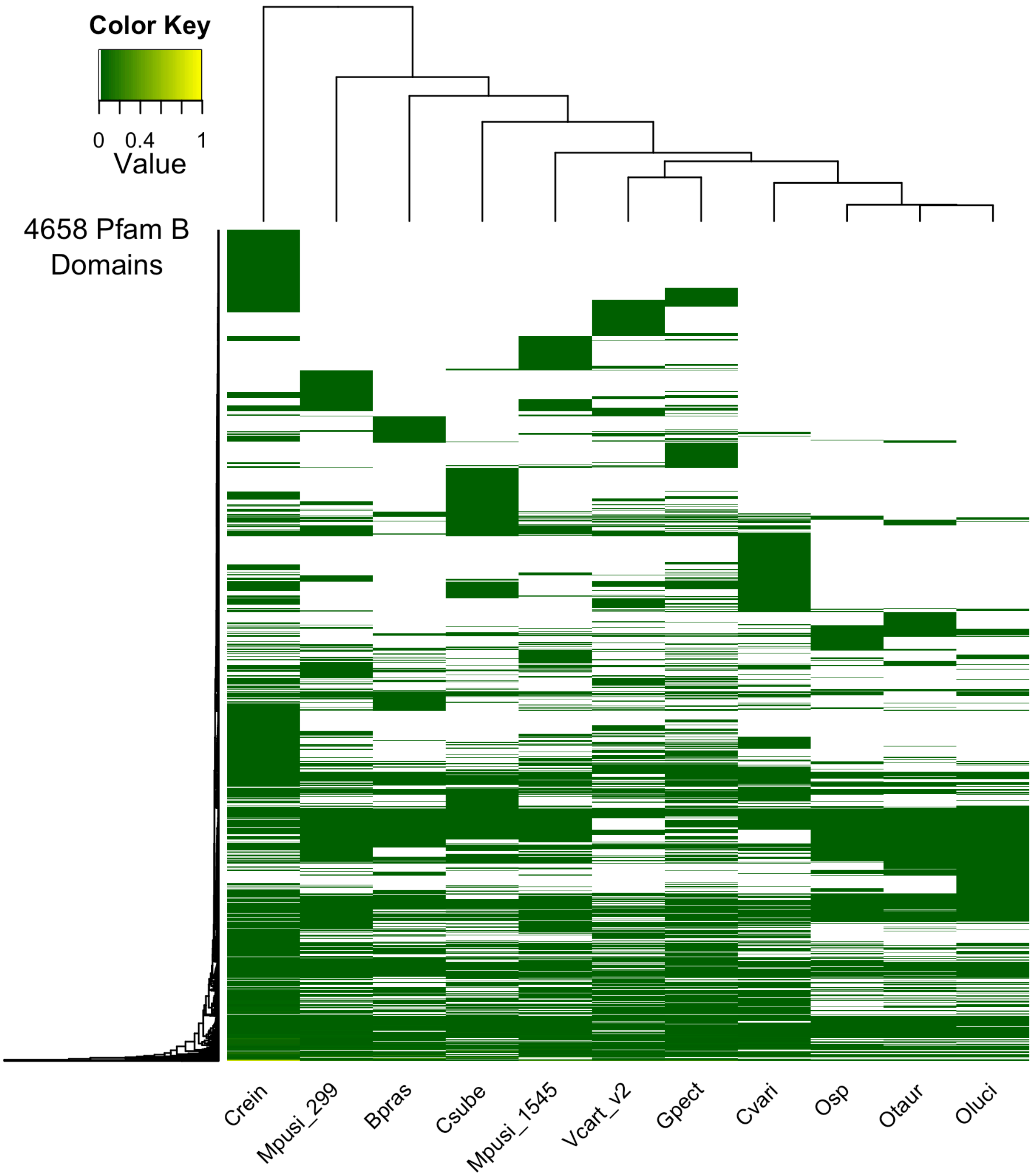
Color Key
0 0.4 1
Value
4658 Pfam B
Domains
Crein
Mpusi_299
Bpras
Csube
Mpusi_1545
Vcart_v2
Gpect
Cvari
Osp
Otaur
Oluci

Supplementary Figure 16. Box-whisker distribution of OrthoMCL cluster size across inflation values ranging from 1.2 to 4.0 including all published Chlorophyte genomes. *Volvox carteri* version 1 was included. Singletons not included. Data points greater than 1.5*IQR above Q_3 are denoted as outliers (dots).

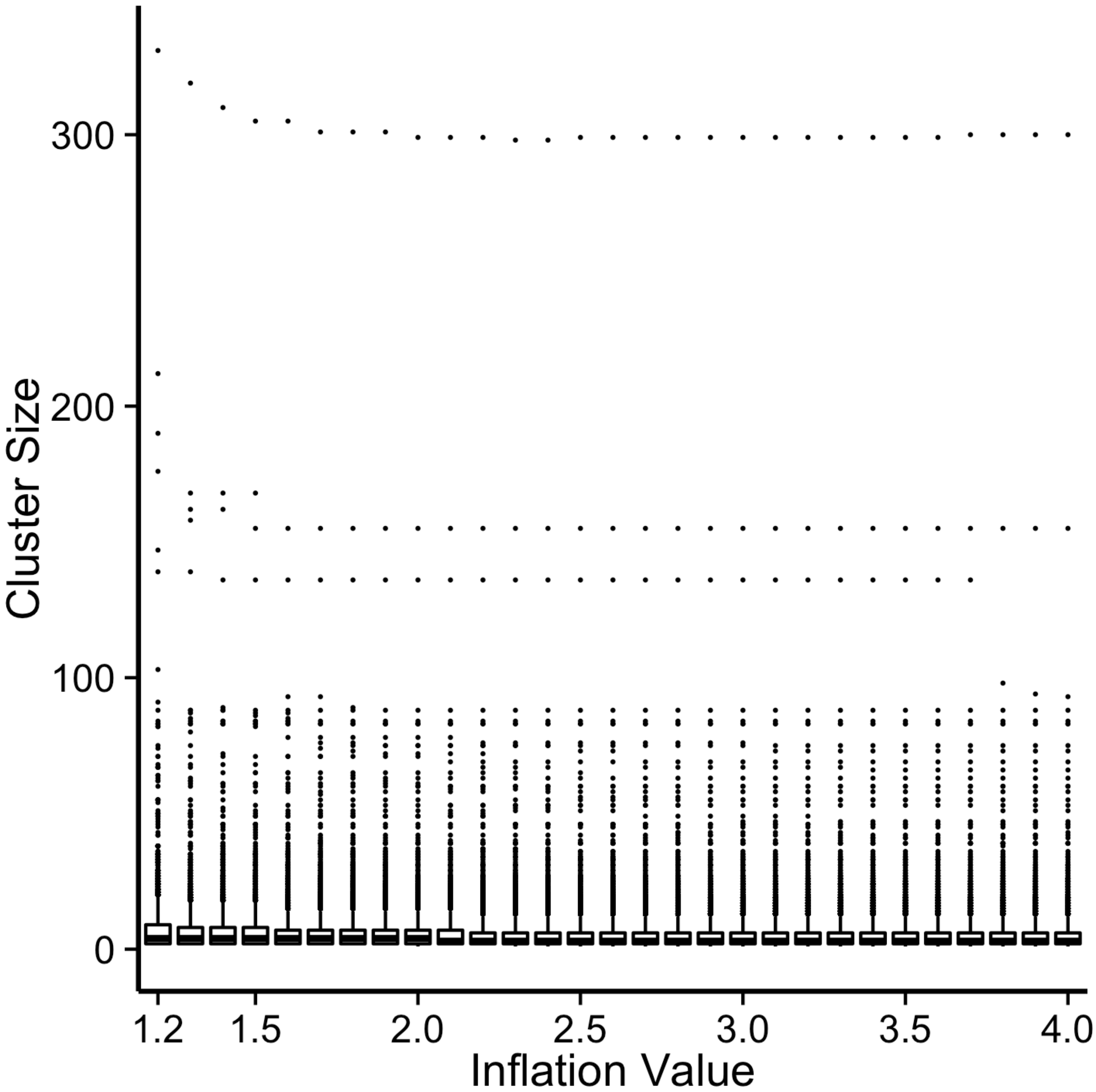

Cluster Size
300
200
100
0
1.2
1.5
2.0
2.5
3.0
3.5
4.0
Inflation Value

Supplementary Figure 17. Box-whisker distribution of OrthoMCL cluster size across inflation values ranging from 1.2 to 4.0 including all published Chlorophyte genomes. *Volvox carteri* version 2 was included. Singletons not included. Data points greater than 1.5*IQR above Q_3 are denoted as outliers (dots).

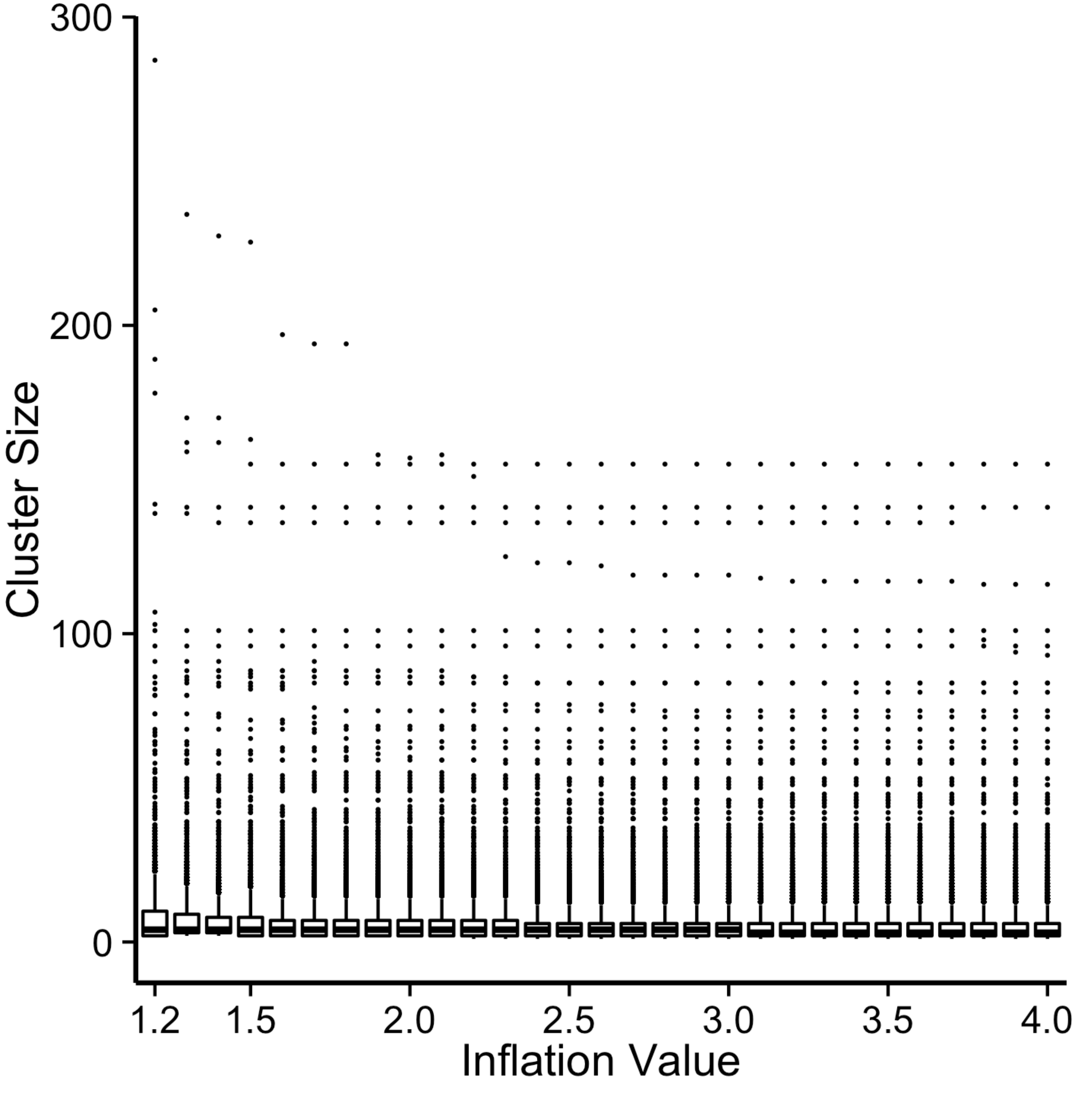
300
200
100
0
Cluster Size
1.2
1.5
2.0
2.5
3.0
3.5
4.0
Inflation Value

Supplementary Figure 18. Number of OrthoMCL clusters for OrthoMCL analysis using the *Volvox carteri* version 1 genome for a range of inflation values. Singletons not included.

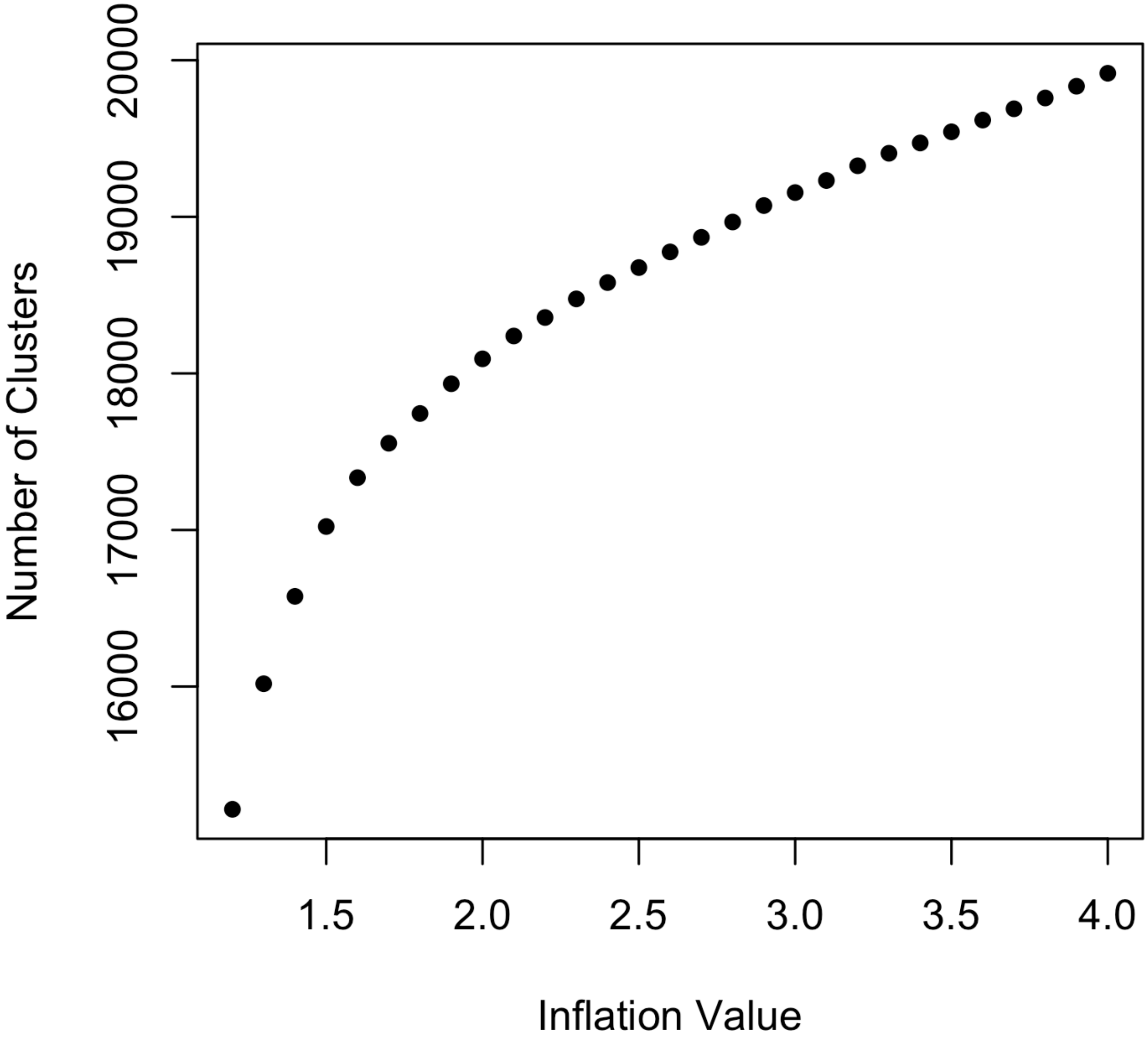

Number of Clusters
16000
17000
18000
19000
20000
1.5
2.0
2.5
3.0
3.5
4.0
Inflation Value

Supplementary Figure 19. Number of OrthoMCL clusters for OrthoMCL analysis using the *Volvox carteri* version 2 genome for a range of inflation values. Singletons not included.

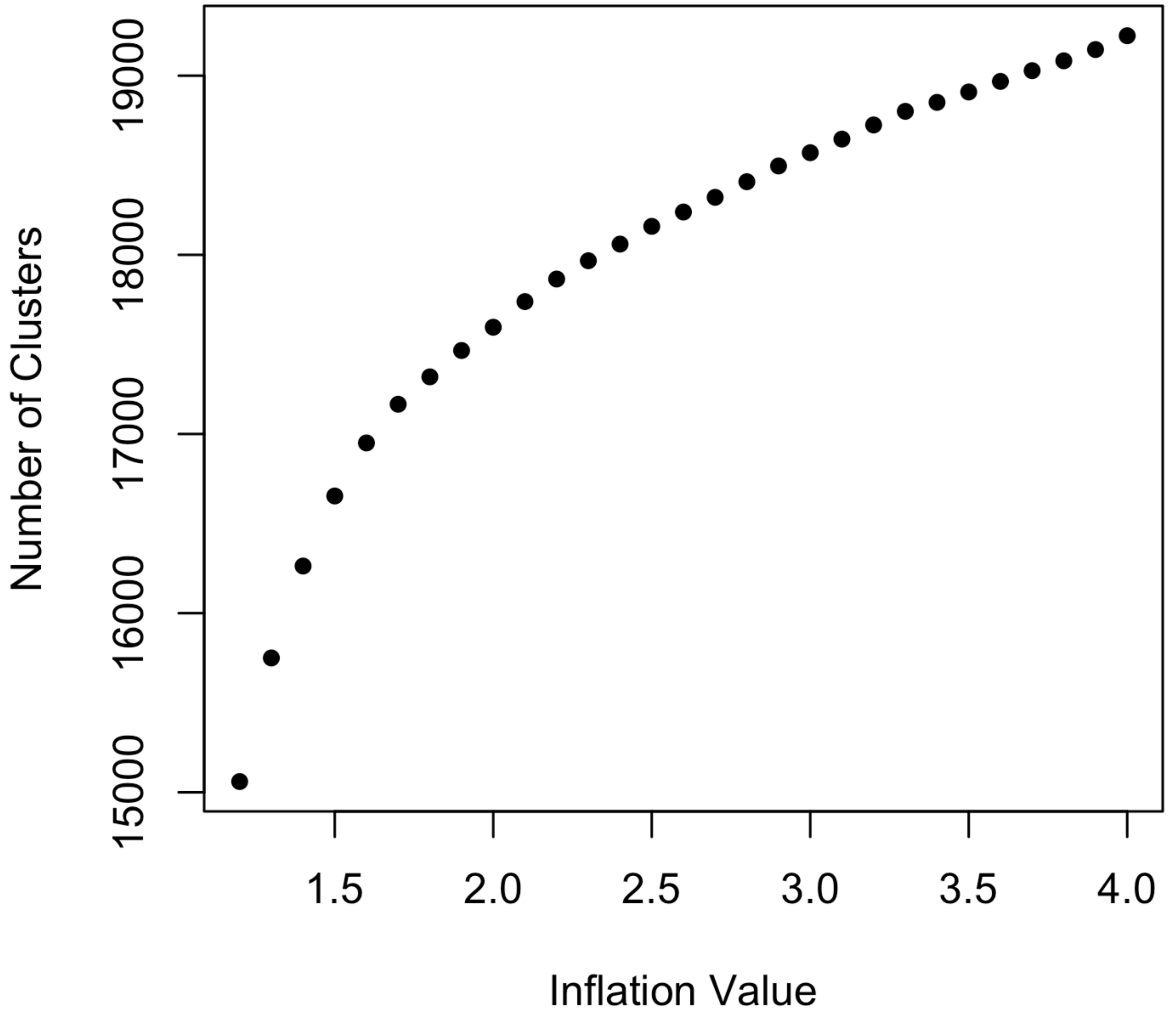
15000
16000
17000
18000
19000
Number of Clusters
1.5
2.0
2.5
3.0
3.5
4.0
Inflation Value

Supplementary Figure 20. Phylogenetic tree of utilized Chlorophyte genomes based on 1,457 genes from single copy OrthoMCL clusters. *Chlamydomonas* is denoted in green, *Gonium* is denoted in blue, and *Volvox* is denoted in black. Other species are in gray. All nodes have bootstrap values equal to 100%.

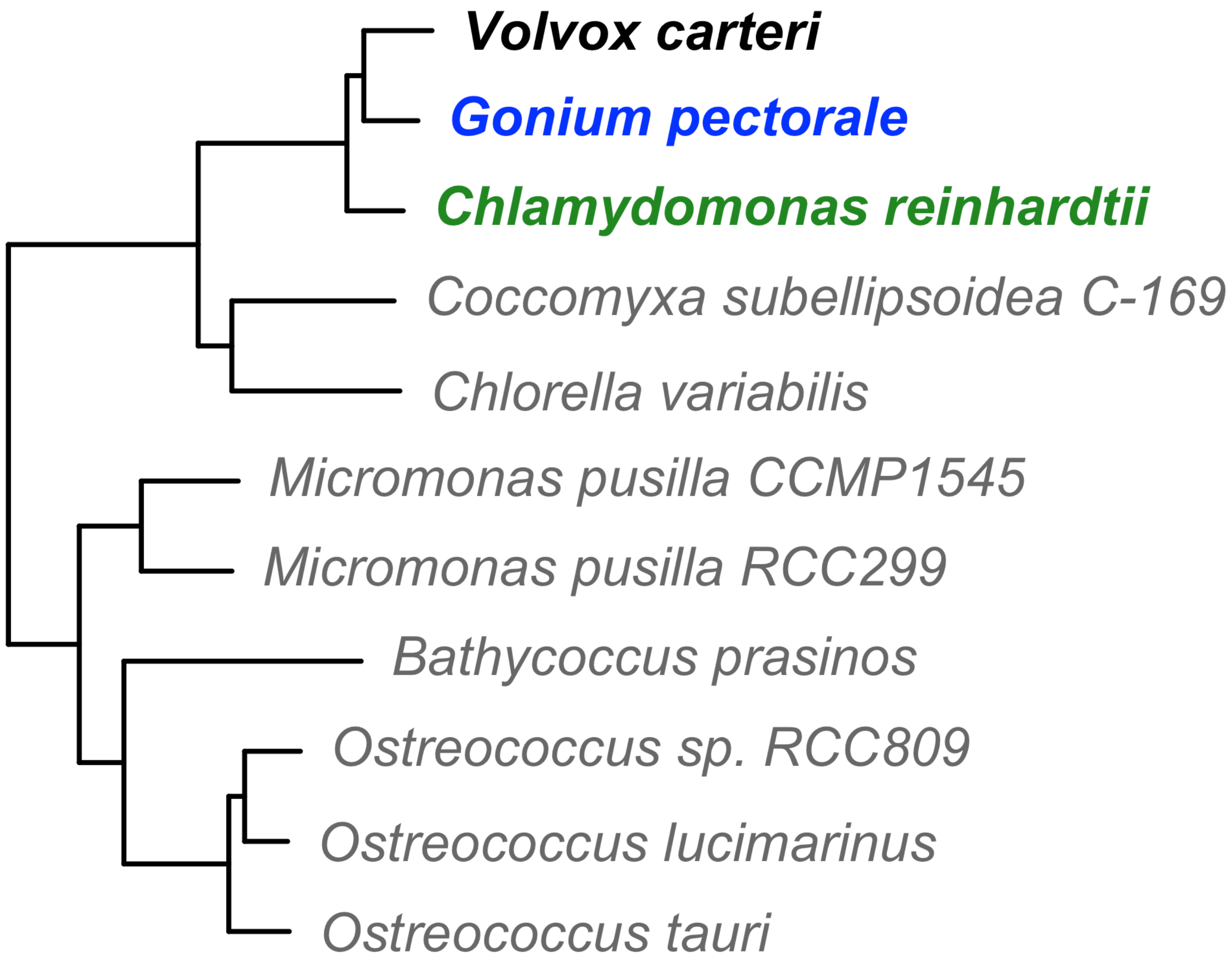
Volvox carteri
Gonium pectorale
Chlamydomonas reinhardtii
Coccomyxa subellipsoidea C-169
Chlorella variabilis
Micromonas pusilla CCMP1545
Micromonas pusilla RCC299
Bathycoccus prasinos
Ostreococcus sp. RCC809
Ostreococcus lucimarinus
Ostreococcus tauri

Supplementary Figure 21. Distributions of genome wide dN (A), dS (B), and dN/dS (C) for 6,154 1:1:1 orthologs in *Chlamydomonas*, *Gonium*, and *Volvox*. Blue histograms denote *Chlamydomonas* and *Gonium* pairwise comparisons, red histograms denote *Chlamydomonas* and *Volvox* pairwise comparisons, and green histograms denote *Gonium* and *Volvox* pairwise comparisons.

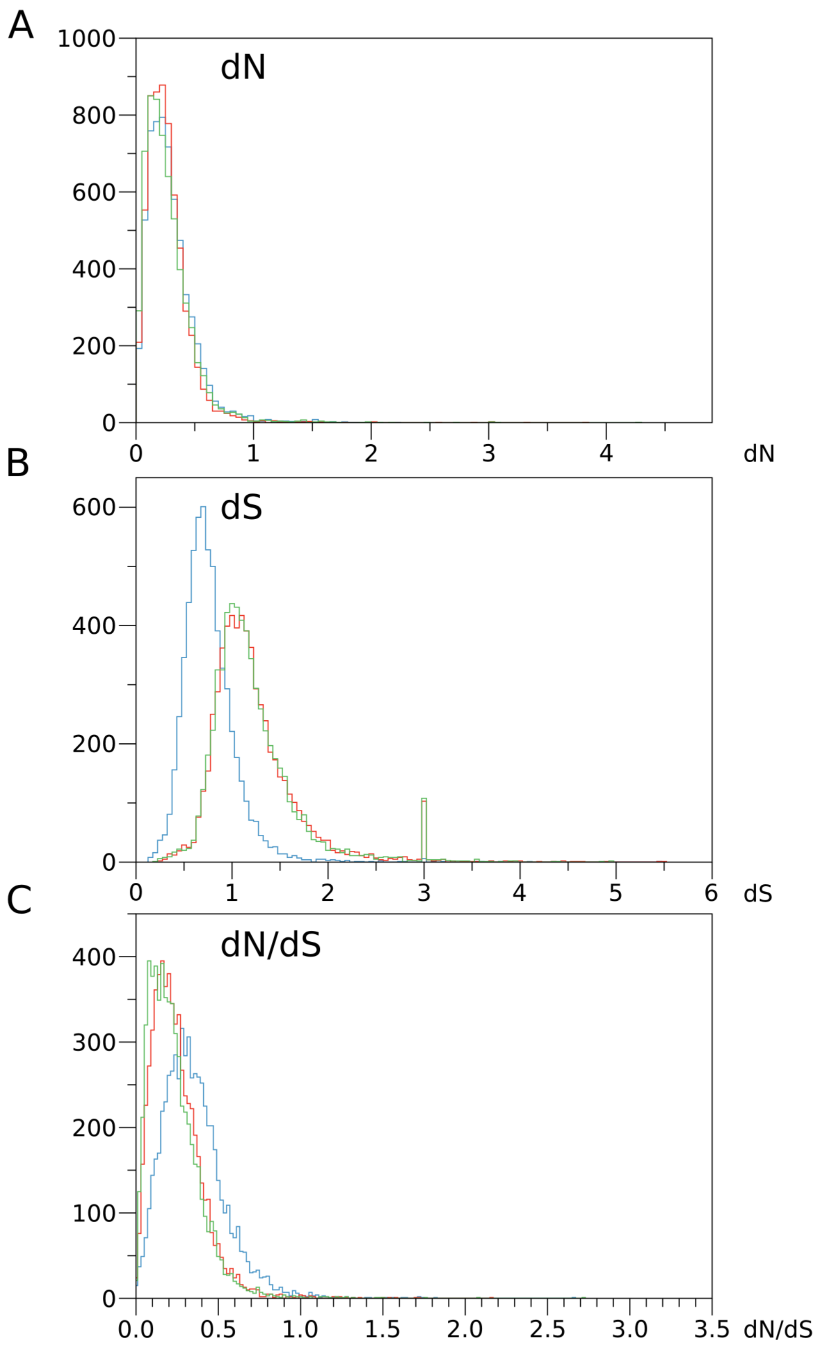
A
1000
800
600
400
200
0
dN
0
1
2
3
4
dN
B
600
400
200
0
dS
0
1
2
3
4
5
6
dS
C
400
300
200
100
0
dN/dS
0.0
0.5
1.0
1.5
2.0
2.5
3.0
3.5
dN/dS

Supplementary Figure 22. Intron conservation in cyclin D genes in *Chlamydomonas* (green), *Gonium* (blue), and *Volvox* (black). Genes with a unique, shared intron are in bold. Black brackets denote the shared intron and surrounding exons. Orange sections denote exons and purple sections denote introns. Scale bars for introns and exons are shown.

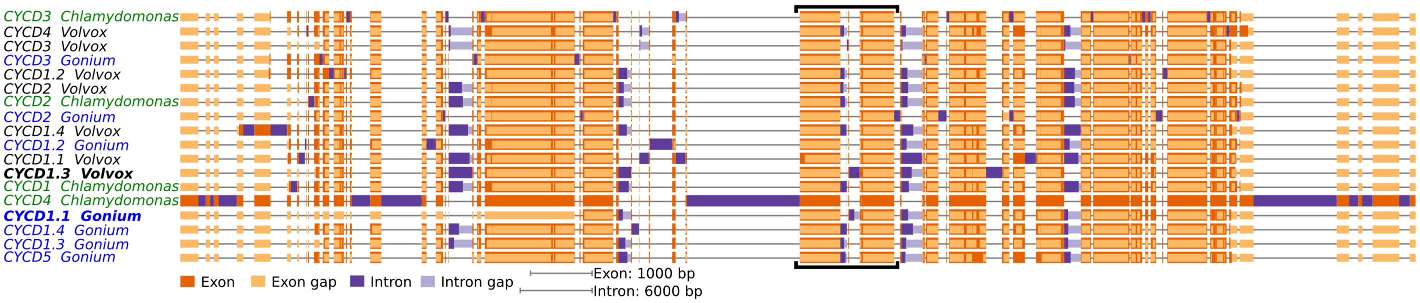

CYCD3 Chlamydomonas
CYCD4 Volvox
CYCD3 Volvox
CYCD3 Gonium
CYCD1.2 Volvox
CYCD2 Volvox
CYCD2 Chlamydomonas
CYCD2 Gonium
CYCD1.4 Volvox
CYCD1.2 Gonium
CYCD1.1 Volvox
CYCD1.3 Volvox
CYCD1 Chlamydomonas
CYCD4 Chlamydomonas
CYCD1.1 Gonium
CYCD1.4 Gonium
CYCD1.3 Gonium
CYCD5 Gonium
Exon
Exon gap
Intron
Intron gap
Exon: 1000 bp
Intron: 6000 bp

Supplementary Figure 23. Intron conservation in cyclin D genes in *Chlamydomonas* (green), *Gonium* (blue), and *Volvox* (black). Genes with a unique, shared intron are in bold and denoted by black arrows. Black brackets denote the shared intron and surrounding exons. Orange sections denote exons and purple sections denote introns. Scale bars for introns and exons are shown.

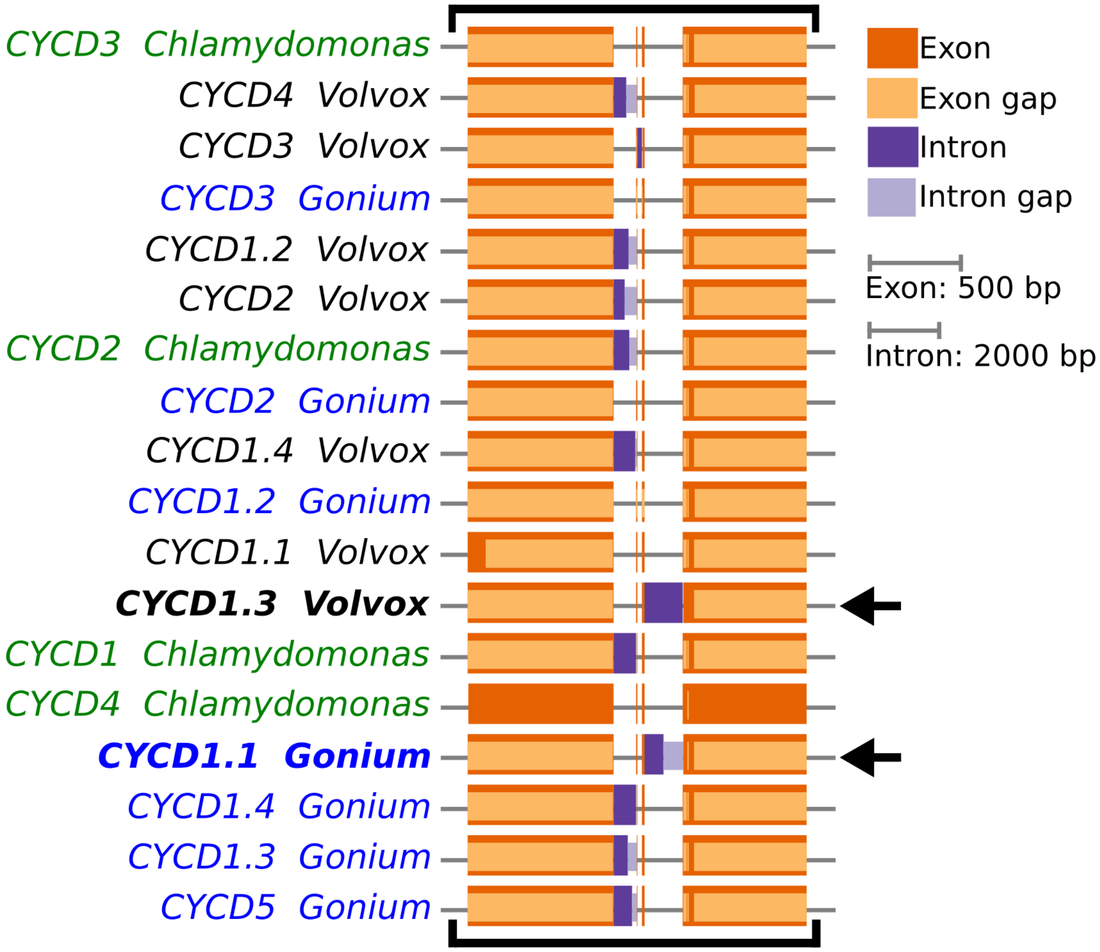
CYCD3 Chlamydomonas
CYCD4 Volvox
CYCD3 Volvox
CYCD3 Gonium
CYCD1.2 Volvox
CYCD2 Volvox
CYCD2 Chlamydomonas
CYCD2 Gonium
CYCD1.4 Volvox
CYCD1.2 Gonium
CYCD1.1 Volvox
CYCD1.3 Volvox
CYCD1 Chlamydomonas
CYCD4 Chlamydomonas
CYCD1.1 Gonium
CYCD1.4 Gonium
CYCD1.3 Gonium
CYCD5 Gonium
Exon
Exon gap
Intron
Intron gap
Exon: 500 bp
Intron: 2000 bp

Supplementary Figure 24. Phylogenetic relationships of matrix metalloprotease genes with and without the canonical metal binding domain[6] in *Chlamydomonas* (green), *Gonium* (blue), and *Volvox* (black) using maximum likelihood methods. The tree is un-rooted and thick branches denote nodes with bootstrap values equal to or greater than 50%. Clades of metalloprotease genes for which pairwise dN/dS values were calculated are numbered 1-4.

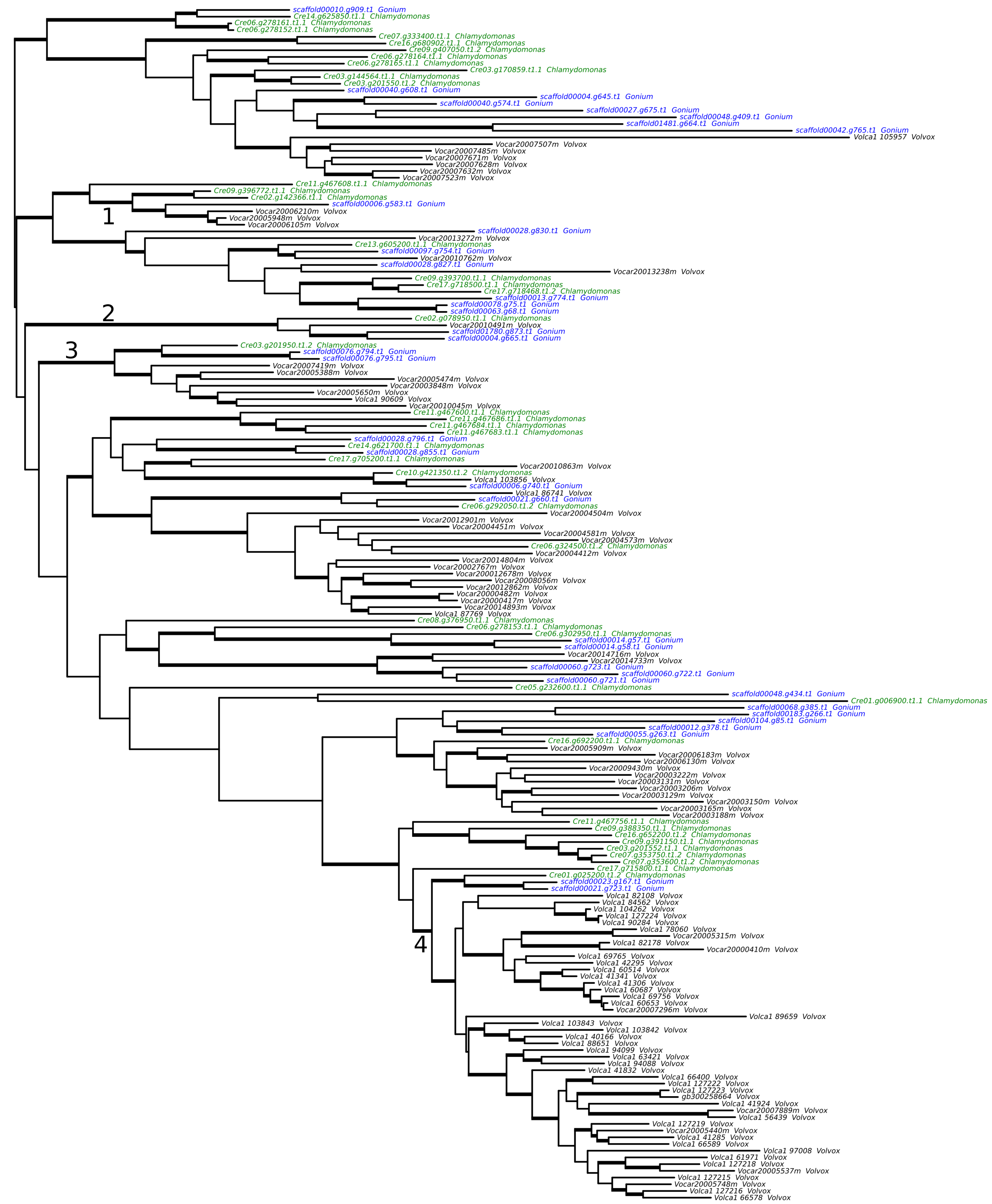

scaffold00010.g909.t1 Gonium
Cre14.g625850.t1.1 Chlamydomonas
Cre06.g278161.t1.1 Chlamydomonas
Cre06.g278152.t1.1 Chlamydomonas
Cre07.g333400.t1.1 Chlamydomonas
Cre16.g680902.t1.1 Chlamydomonas
Cre09.g407050.t1.2 Chlamydomonas
Cre06.g278164.t1.1 Chlamydomonas
Cre06.g278165.t1.1 Chlamydomonas
Cre03.g170859.t1.1 Chlamydomonas
Cre03.g144564.t1.1 Chlamydomonas
Cre03.g201550.t1.2 Chlamydomonas
scaffold00040.g608.t1 Gonium
scaffold00004.g645.t1 Gonium
scaffold00040.g574.t1 Gonium
scaffold00027.g675.t1 Gonium
scaffold00048.g409.t1 Gonium
scaffold01481.g664.t1 Gonium
scaffold00042.g765.t1 Gonium
Volca1 105957 Volvox
Vocar20007507m Volvox
Vocar20007485m Volvox
Vocar20007671m Volvox
Vocar20007628m Volvox
Vocar20007632m Volvox
Vocar20007523m Volvox
Cre11.g467608.t1.1 Chlamydomonas
Cre09.g396772.t1.1 Chlamydomonas
Cre02.g142366.t1.1 Chlamydomonas
scaffold00006.g583.t1 Gonium
1
Vocar20006210m Volvox
Vocar20005948m Volvox
Vocar20006105m Volvox
scaffold00028.g830.t1 Gonium
Vocar20013272m Volvox
Cre13.g605200.t1.1 Chlamydomonas
scaffold00097.g754.t1 Gonium
Vocar20010762m Volvox
scaffold00028.g827.t1 Gonium
Vocar20013238m Volvox
Cre09.g393700.t1.1 Chlamydomonas
Cre17.g718500.t1.1 Chlamydomonas
Cre17.g718468.t1.2 Chlamydomonas
scaffold00013.g774.t1 Gonium
scaffold00078.g75.t1 Gonium
scaffold00063.g68.t1 Gonium
2
Cre02.g078950.t1.1 Chlamydomonas
Vocar20010491m Volvox
scaffold01780.g873.t1 Gonium
scaffold00004.g665.t1 Gonium
3
Cre03.g201950.t1.2 Chlamydomonas
scaffold00076.g794.t1 Gonium
scaffold00076.g795.t1 Gonium
Vocar20007419m Volvox
Vocar20005388m Volvox
Vocar20005474m Volvox
Vocar20003848m Volvox
Vocar20005650m Volvox
Volca1 90609 Volvox
Vocar20010045m Volvox
Cre11.g467600.t1.1 Chlamydomonas
Cre11.g467686.t1.1 Chlamydomonas
Cre11.g467684.t1.1 Chlamydomonas
Cre11.g467683.t1.1 Chlamydomonas
scaffold00028.g796.t1 Gonium
Cre14.g621700.t1.1 Chlamydomonas
scaffold00028.g855.t1 Gonium
Cre17.g705200.t1.1 Chlamydomonas
Vocar20010863m Volvox
Cre10.g421350.t1.2 Chlamydomonas
Volca1 103856 Volvox
scaffold00006.g740.t1 Gonium
Volca1 86741 Volvox
scaffold00021.g660.t1 Gonium
Cre06.g292050.t1.2 Chlamydomonas
Vocar20004504m Volvox
Vocar20012901m Volvox
Vocar20004451m Volvox
Vocar20004581m Volvox
Vocar20004573m Volvox
Cre06.g324500.t1.2 Chlamydomonas
Vocar20004412m Volvox
Vocar20014804m Volvox
Vocar20002767m Volvox
Vocar20012678m Volvox
Vocar20008056m Volvox
Vocar20012862m Volvox
Vocar20000482m Volvox
Vocar20000417m Volvox
Vocar20014893m Volvox
Volca1 87769 Volvox
Cre08.g376950.t1.1 Chlamydomonas
Cre06.g278153.t1.1 Chlamydomonas
Cre06.g302950.t1.1 Chlamydomonas
scaffold00014.g57.t1 Gonium
scaffold00014.g58.t1 Gonium
Vocar20014716m Volvox
Vocar20014733m Volvox
scaffold00060.g723.t1 Gonium
scaffold00060.g722.t1 Gonium
scaffold00060.g721.t1 Gonium
Cre05.g232600.t1.1 Chlamydomonas
scaffold00048.g434.t1 Gonium
Cre01.g006900.t1.1 Chlamydomonas
scaffold00068.g385.t1 Gonium
scaffold00183.g266.t1 Gonium
scaffold00104.g85.t1 Gonium
scaffold00012.g378.t1 Gonium
scaffold00055.g263.t1 Gonium
Cre16.g692200.t1.1 Chlamydomonas
Vocar20005909m Volvox
Vocar20006183m Volvox
Vocar20006130m Volvox
Vocar20009430m Volvox
Vocar20003222m Volvox
Vocar20003131m Volvox
Vocar20003206m Volvox
Vocar20003129m Volvox
Vocar20003150m Volvox
Vocar20003165m Volvox
Vocar20003188m Volvox
Cre11.g467756.t1.1 Chlamydomonas
Cre09.g388350.t1.1 Chlamydomonas
Cre16.g652200.t1.2 Chlamydomonas
Cre09.g391150.t1.1 Chlamydomonas
Cre03.g201552.t1.1 Chlamydomonas
Cre07.g353750.t1.2 Chlamydomonas
Cre07.g353600.t1.2 Chlamydomonas
Cre17.g715800.t1.1 Chlamydomonas
Cre01.g025200.t1.2 Chlamydomonas
scaffold00023.g167.t1 Gonium
scaffold00021.g723.t1 Gonium
Volca1 82108 Volvox
Volca1 84562 Volvox
Volca1 104262 Volvox
Volca1 127224 Volvox
Volca1 90284 Volvox
Volca1 78060 Volvox
Vocar20005315m Volvox
4
Volca1 82178 Volvox
Vocar20000410m Volvox
Volca1 69765 Volvox
Volca1 42295 Volvox
Volca1 60514 Volvox
Volca1 41341 Volvox
Volca1 41306 Volvox
Volca1 60687 Volvox
Volca1 69756 Volvox
Volca1 60653 Volvox
Vocar20007296m Volvox
Volca1 89659 Volvox
Volca1 103843 Volvox
Volca1 103842 Volvox
Volca1 40166 Volvox
Volca1 88651 Volvox
Volca1 94099 Volvox
Volca1 63421 Volvox
Volca1 94088 Volvox
Volca1 41832 Volvox
Volca1 66400 Volvox
Volca1 127222 Volvox
Volca1 127223 Volvox
gb300258664 Volvox
Volca1 41924 Volvox
Vocar20007889m Volvox
Volca1 56439 Volvox
Volca1 127219 Volvox
Vocar20005440m Volvox
Volca1 41285 Volvox
Volca1 66589 Volvox
Volca1 97008 Volvox
Volca1 61971 Volvox
Volca1 127218 Volvox
Vocar20005537m Volvox
Volca1 127215 Volvox
Vocar20005748m Volvox
Volca1 127216 Volvox
Volca1 66578 Volvox

Supplementary Figure 25. Phylogenetic relationships of matrix metalloprotease genes with the canonical metal binding domain[6] in *Chlamydomonas* (green), *Gonium* (blue), and *Volvox* (black) using maximum likelihood methods. The tree is un-rooted and numerical values represent bootstrap values when equal to or greater than 50%.

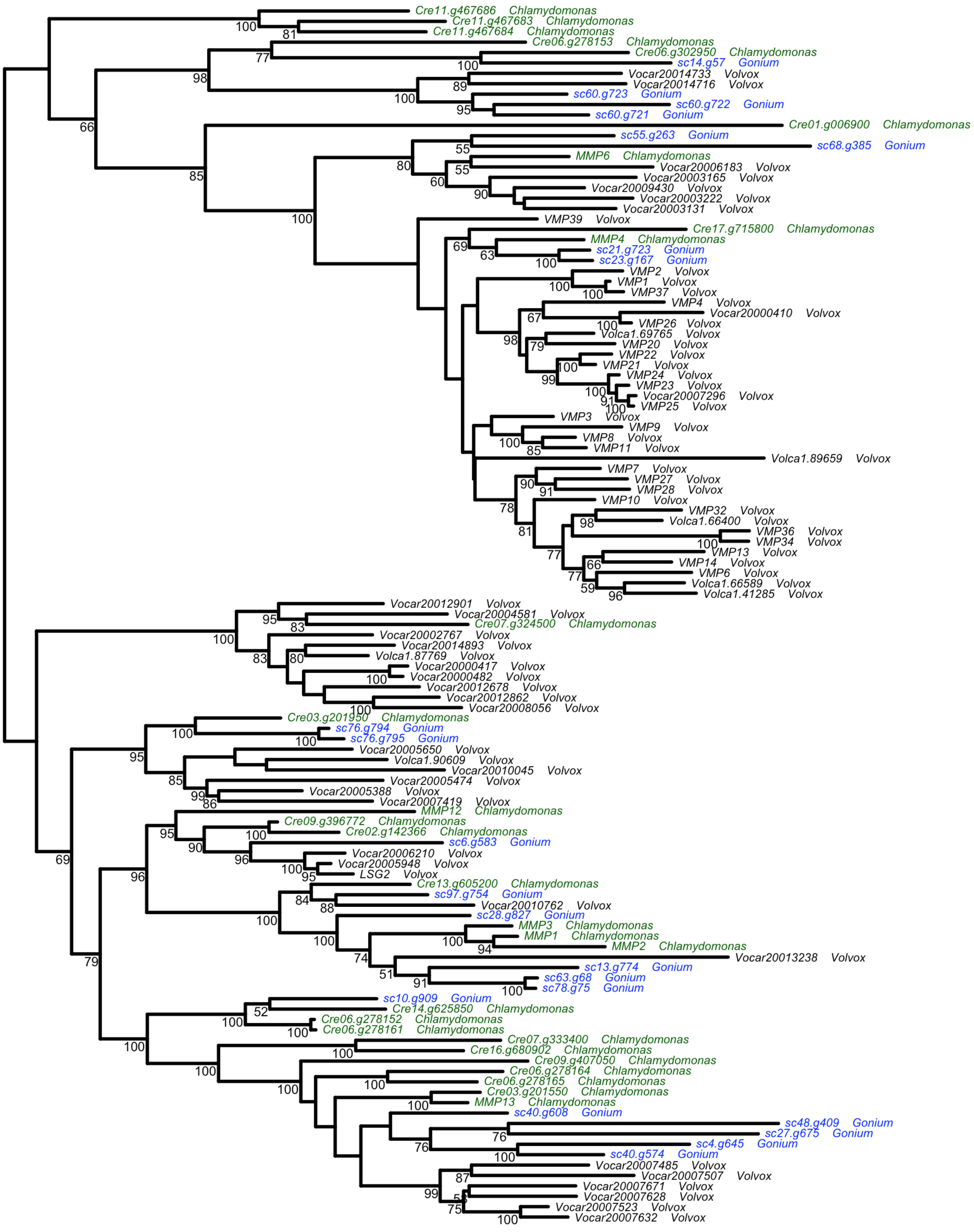
Cre11.g467686 Chlamydomonas
Cre11.g467683 Chlamydomonas
Cre11.g467684 Chlamydomonas
Cre06.g278153 Chlamydomonas
Cre06.g302950 Chlamydomonas
sc14.g57 Gonium
Vocar20014733 Volvox
Vocar20014716 Volvox
sc60.g723 Gonium
sc60.g722 Gonium
sc60.g721 Gonium
Cre01.g006900 Chlamydomonas
sc55.g263 Gonium
sc68.g385 Gonium
MMP6 Chlamydomonas
Vocar20006183 Volvox
Vocar20003165 Volvox
Vocar20009430 Volvox
Vocar20003222 Volvox
Vocar20003131 Volvox
VMP39 Volvox
Cre17.g715800 Chlamydomonas
MMP4 Chlamydomonas
sc21.g723 Gonium
sc23.g167 Gonium
VMP2 Volvox
VMP1 Volvox
VMP37 Volvox
VMP4 Volvox
Vocar20000410 Volvox
VMP26 Volvox
Volca1.69765 Volvox
VMP20 Volvox
VMP22 Volvox
VMP21 Volvox
VMP24 Volvox
VMP23 Volvox
Vocar20007296 Volvox
VMP25 Volvox
VMP3 Volvox
VMP9 Volvox
VMP8 Volvox
VMP11 Volvox
Volca1.89659 Volvox
VMP7 Volvox
VMP27 Volvox
VMP28 Volvox
VMP10 Volvox
VMP32 Volvox
Volca1.66400 Volvox
VMP36 Volvox
VMP34 Volvox
VMP13 Volvox
VMP14 Volvox
VMP6 Volvox
Volca1.66589 Volvox
Volca1.41285 Volvox
Vocar20012901 Volvox
Vocar20004581 Volvox
Cre07.g324500 Chlamydomonas
Vocar20002767 Volvox
Vocar20014893 Volvox
Volca1.87769 Volvox
Vocar20000417 Volvox
Vocar20000482 Volvox
Vocar20012678 Volvox
Vocar20012862 Volvox
Vocar20008056 Volvox
Cre03.g201950 Chlamydomonas
sc76.g794 Gonium
sc76.g795 Gonium
Vocar20005650 Volvox
Volca1.90609 Volvox
Vocar20010045 Volvox
Vocar20005474 Volvox
Vocar20005388 Volvox
Vocar20007419 Volvox
MMP12 Chlamydomonas
Cre09.g396772 Chlamydomonas
Cre02.g142366 Chlamydomonas
sc6.g583 Gonium
Vocar20006210 Volvox
Vocar20005948 Volvox
LSG2 Volvox
Cre13.g605200 Chlamydomonas
sc97.g754 Gonium
Vocar20010762 Volvox
sc28.g827 Gonium
MMP3 Chlamydomonas
MMP1 Chlamydomonas
MMP2 Chlamydomonas
Vocar20013238 Volvox
sc13.g774 Gonium
sc63.g68 Gonium
sc78.g75 Gonium
sc10.g909 Gonium
Cre14.g625850 Chlamydomonas
Cre06.g278152 Chlamydomonas
Cre06.g278161 Chlamydomonas
Cre07.g333400 Chlamydomonas
Cre16.g680902 Chlamydomonas
Cre09.g407050 Chlamydomonas
Cre06.g278164 Chlamydomonas
Cre06.g278165 Chlamydomonas
Cre03.g201550 Chlamydomonas
MMP13 Chlamydomonas
sc40.g608 Gonium
sc48.g409 Gonium
sc27.g675 Gonium
sc4.g645 Gonium
sc40.g574 Gonium
Vocar20007485 Volvox
Vocar20007507 Volvox
Vocar20007671 Volvox
Vocar20007628 Volvox
Vocar20007523 Volvox
Vocar20007632 Volvox

Supplementary Figure 26. Phylogenetic relationships of matrix metalloprotease genes without the canonical metal binding domain[6] in *Chlamydomonas* (green), *Gonium* (blue), and *Volvox* (black) using maximum likelihood methods. The tree is un-rooted and numerical values represent bootstrap values when equal to or greater than 50%.

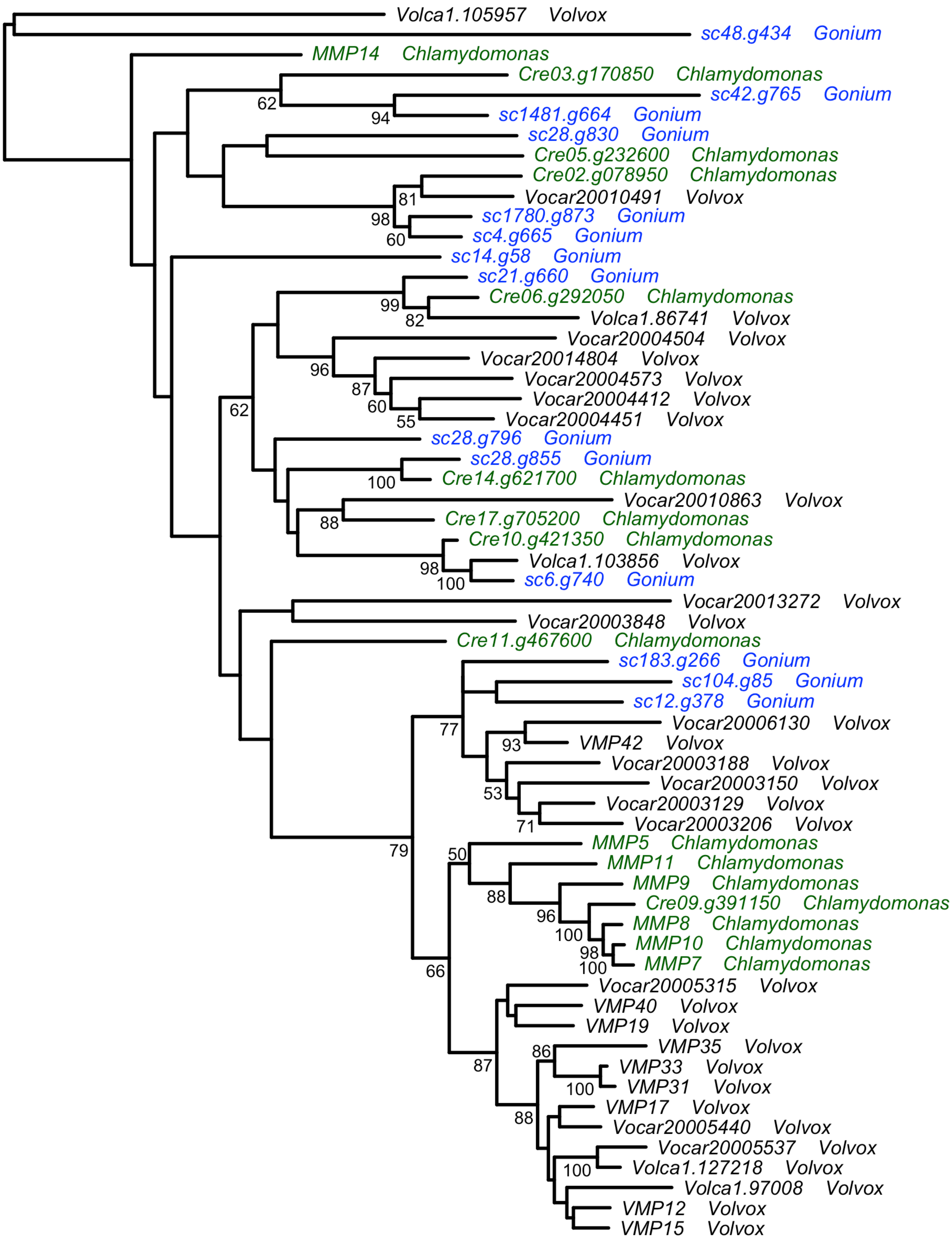
Volca1.105957 Volvox
sc48.g434 Gonium
MMP14 Chlamydomonas
Cre03.g170850 Chlamydomonas
sc42.g765 Gonium
sc1481.g664 Gonium
sc28.g830 Gonium
Cre05.g232600 Chlamydomonas
Cre02.g078950 Chlamydomonas
Vocar20010491 Volvox
sc1780.g873 Gonium
sc4.g665 Gonium
sc14.g58 Gonium
sc21.g660 Gonium
Cre06.g292050 Chlamydomonas
Volca1.86741 Volvox
Vocar20004504 Volvox
Vocar20014804 Volvox
Vocar20004573 Volvox
Vocar20004412 Volvox
Vocar20004451 Volvox
sc28.g796 Gonium
sc28.g855 Gonium
Cre14.g621700 Chlamydomonas
Vocar20010863 Volvox
Cre17.g705200 Chlamydomonas
Cre10.g421350 Chlamydomonas
Volca1.103856 Volvox
sc6.g740 Gonium
Vocar20013272 Volvox
Vocar20003848 Volvox
Cre11.g467600 Chlamydomonas
sc183.g266 Gonium
sc104.g85 Gonium
sc12.g378 Gonium
Vocar20006130 Volvox
VMP42 Volvox
Vocar20003188 Volvox
Vocar20003150 Volvox
Vocar20003129 Volvox
Vocar20003206 Volvox
MMP5 Chlamydomonas
MMP11 Chlamydomonas
MMP9 Chlamydomonas
Cre09.g391150 Chlamydomonas
MMP8 Chlamydomonas
MMP10 Chlamydomonas
MMP7 Chlamydomonas
Vocar20005315 Volvox
VMP40 Volvox
VMP19 Volvox
VMP35 Volvox
VMP33 Volvox
VMP31 Volvox
VMP17 Volvox
Vocar20005440 Volvox
Vocar20005537 Volvox
Volca1.127218 Volvox
Volca1.97008 Volvox
VMP12 Volvox
VMP15 Volvox
62
94
81
98
60
99
82
96
87
60
55
62
100
88
98
100
77
93
53
71
79
50
88
96
100
98
100
66
86
100
87
88
100

Supplementary Figure 27. Phylogenetic relationships of pherophorin cell wall genes in *Chlamydomonas* (green), *Gonium* (blue), and *Volvox* (black) using maximum likelihood methods and full gene alignments. The tree is un-rooted and thick branches denote nodes with bootstrap values equal to or greater than 50%. Clades of pherophorin genes for which pairwise dN/dS values were calculated are numbered 1-5.

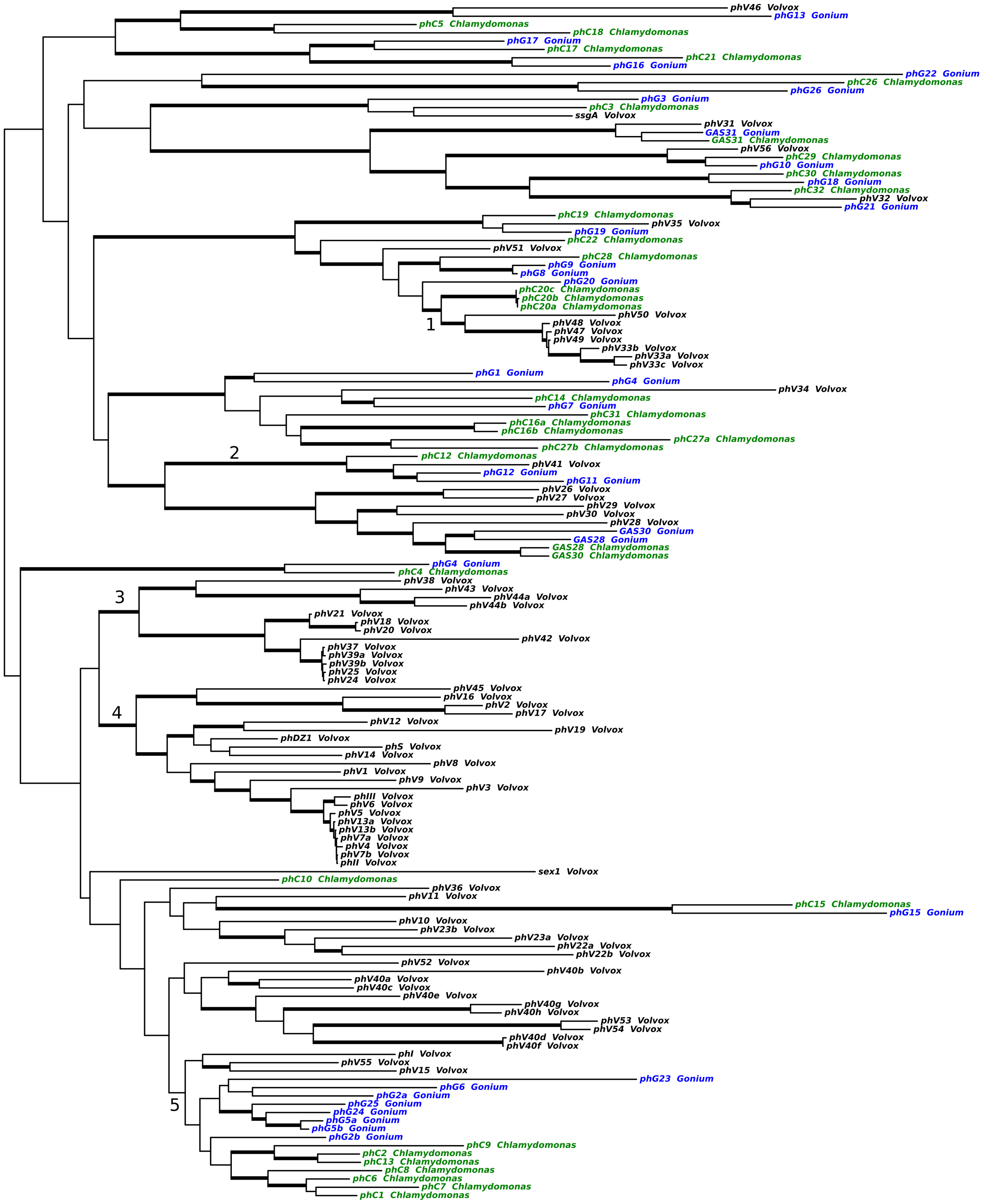

phV46 Volvox
phG13 Gonium
phC5 Chlamydomonas
phC18 Chlamydomonas
phG17 Gonium
phC17 Chlamydomonas
phC21 Chlamydomonas
phG16 Gonium
phG22 Gonium
phC26 Chlamydomonas
phG26 Gonium
phG3 Gonium
phC3 Chlamydomonas
ssgA Volvox
phV31 Volvox
GAS31 Gonium
GAS31 Chlamydomonas
phV56 Volvox
phC29 Chlamydomonas
phG10 Gonium
phC30 Chlamydomonas
phG18 Gonium
phC32 Chlamydomonas
phV32 Volvox
phG21 Gonium
phC19 Chlamydomonas
phV35 Volvox
phG19 Gonium
phC22 Chlamydomonas
phV51 Volvox
phC28 Chlamydomonas
phG9 Gonium
phG8 Gonium
phG20 Gonium
phC20c Chlamydomonas
phC20b Chlamydomonas
phC20a Chlamydomonas
phV50 Volvox
1
phV48 Volvox
phV47 Volvox
phV49 Volvox
phV33b Volvox
phV33a Volvox
phV33c Volvox
phG1 Gonium
phG4 Gonium
phV34 Volvox
phC14 Chlamydomonas
phG7 Gonium
phC31 Chlamydomonas
phC16a Chlamydomonas
phC16b Chlamydomonas
phC27a Chlamydomonas
phC27b Chlamydomonas
2
phC12 Chlamydomonas
phV41 Volvox
phG12 Gonium
phG11 Gonium
phV26 Volvox
phV27 Volvox
phV29 Volvox
phV30 Volvox
phV28 Volvox
GAS30 Gonium
GAS28 Gonium
GAS28 Chlamydomonas
GAS30 Chlamydomonas
phG4 Gonium
phC4 Chlamydomonas
phV38 Volvox
3
phV43 Volvox
phV44a Volvox
phV44b Volvox
phV21 Volvox
phV18 Volvox
phV20 Volvox
phV42 Volvox
phV37 Volvox
phV39a Volvox
phV39b Volvox
phV25 Volvox
phV24 Volvox
phV45 Volvox
phV16 Volvox
4
phV2 Volvox
phV17 Volvox
phV12 Volvox
phV19 Volvox
phDZ1 Volvox
phS Volvox
phV14 Volvox
phV8 Volvox
phV1 Volvox
phV9 Volvox
phV3 Volvox
phIII Volvox
phV6 Volvox
phV5 Volvox
phV13a Volvox
phV13b Volvox
phV7a Volvox
phV4 Volvox
phV7b Volvox
phII Volvox
sex1 Volvox
phC10 Chlamydomonas
phV36 Volvox
phV11 Volvox
phC15 Chlamydomonas
phG15 Gonium
phV10 Volvox
phV23b Volvox
phV23a Volvox
phV22a Volvox
phV22b Volvox
phV52 Volvox
phV40b Volvox
phV40a Volvox
phV40c Volvox
phV40e Volvox
phV40g Volvox
phV40h Volvox
phV53 Volvox
phV54 Volvox
phV40d Volvox
phV40f Volvox
phI Volvox
phV55 Volvox
phV15 Volvox
phG23 Gonium
phG6 Gonium
phG2a Gonium
5
phG25 Gonium
phG24 Gonium
phG5a Gonium
phG5b Gonium
phG2b Gonium
phC9 Chlamydomonas
phC2 Chlamydomonas
phC13 Chlamydomonas
phC8 Chlamydomonas
phC6 Chlamydomonas
phC7 Chlamydomonas
phC1 Chlamydomonas

Supplementary Figure 28. Phylogenetic relationships of *GP2* and *GP3* genes in *Chlamydomonas* (green), *Gonium* (blue), and *Volvox* (black) using maximum likelihood methods. The tree is a mid-point root and numerical values represent bootstrap values when equal to or greater than 50%.

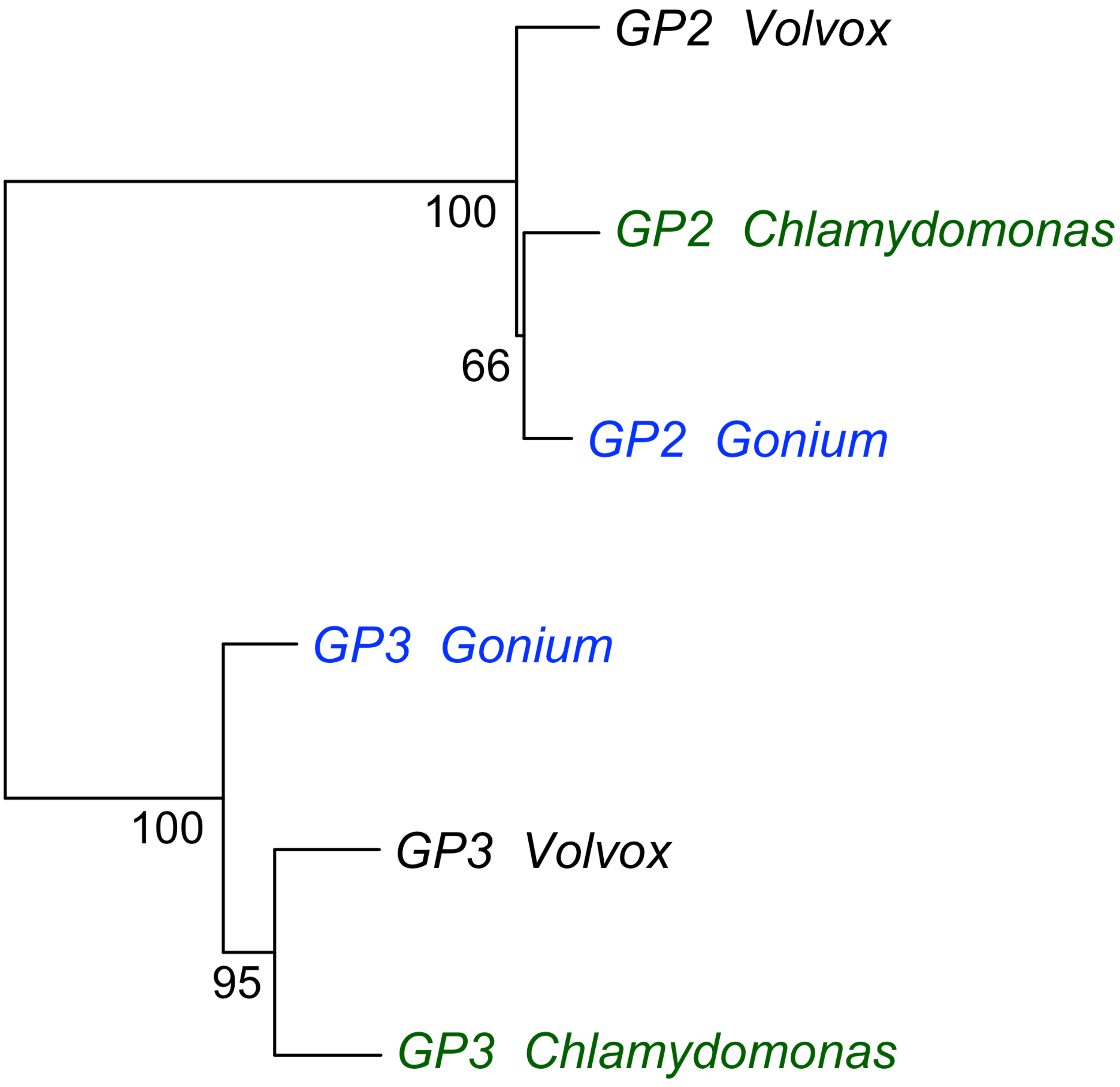
GP2 Volvox
100
GP2 Chlamydomonas
66
GP2 Gonium
GP3 Gonium
100
GP3 Volvox
95
GP3 Chlamydomonas

Supplementary Figure 29. Box-whisker distribution of pairwise dN/dS values for ECM subtrees from Supplementary Figure 24 and Supplementary Figure 27. Data points are included with a random jitter from the vertical axis.

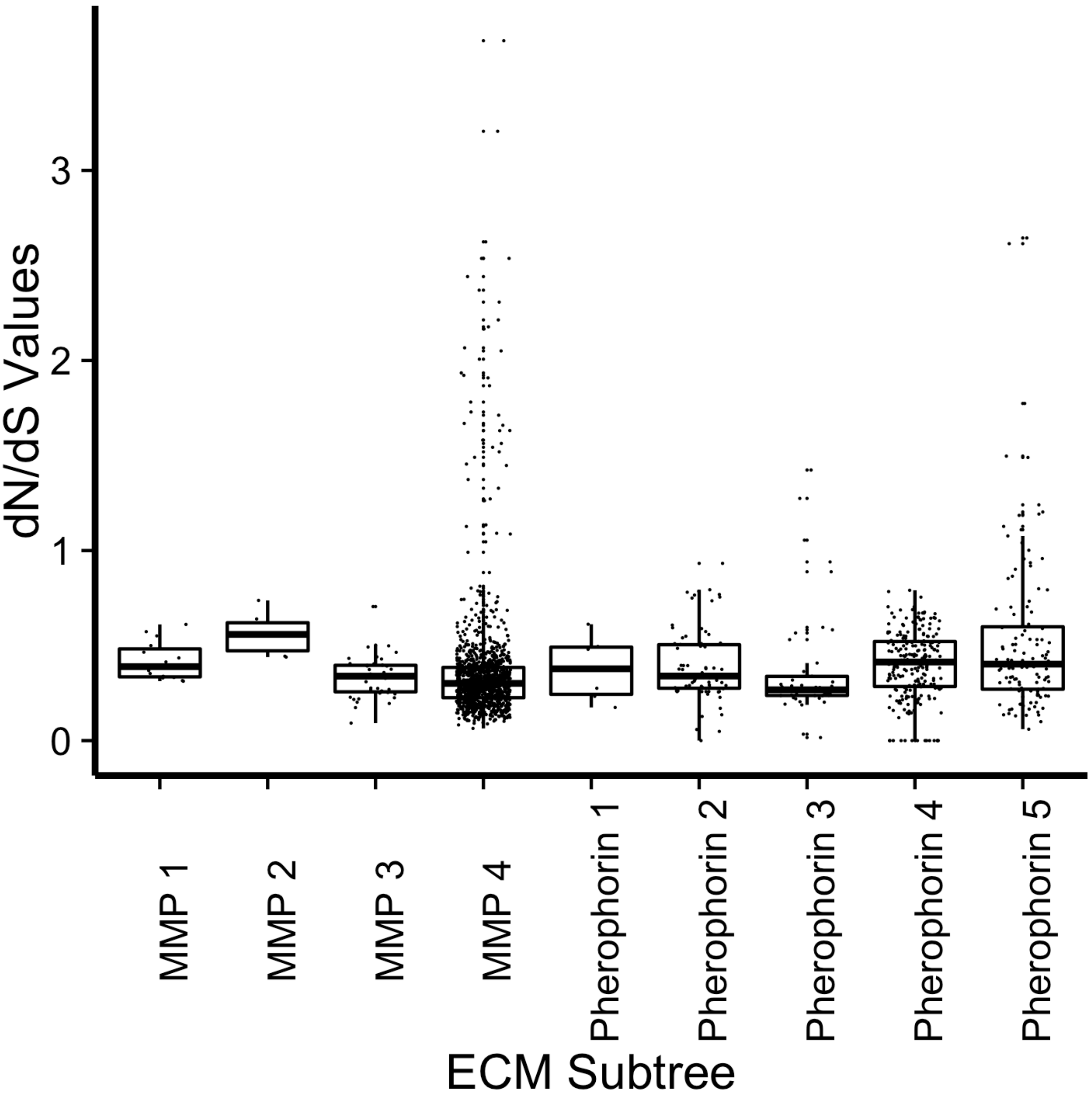

dN/dS Values
0
1
2
3
MMP 1
MMP 2
MMP 3
MMP 4
Pherophorin 1
Pherophorin 2
Pherophorin 3
Pherophorin 4
Pherophorin 5
ECM Subtree

Supplementary Tables

Supplementary Table 1. The number of proteins predicted in each Transcription Associated Protein (TAP) family in all published green algae genomes.

Transcription Factor Family	*Bathycoccus prasinos*	*Chlamydomonas reinhardtii*	*Coccomyxa subellipsoidea*	*Chlorella variabilis*	*Gonium pectorale*	*Micromonas pusilla CCMP1545*	*Micromonas pusilla RCC299*	*Ostreococcus lucimarinus*	*Ostreococcus sp RCC809*	*Ostreococcus tauri*	*Volvox carteri v1*
AP2	7	22	19	14	16	16	14	9	7	8	24
ARID	2	4	9	3	3	5	5	5	3	1	3
ARR-B	1	1	1	1	1	1	1	1	1	1	1
Alfin-like	3	3	4	5	3	3	4	7	5	3	4
B3	0	2	3	5	2	1	1	0	0	0	1
BSD	2	3	3	3	3	2	3	2	2	2	3
C2C2-CO-like	0	1	1	1	1	1	3	1	2	2	1
C2C2-GATA	7	14	7	9	8	11	8	9	5	5	11
C2H2	49	6	2	2	4	8	9	3	2	1	2
C3H	13	20	15	19	17	16	20	12	14	12	15
CPP	2	3	3	3	3	2	1	2	2	2	3
CSD	2	2	3	3	2	4	4	3	2	4	2
Coactivator p15	1	1	1	1	1	2	1	1	1	1	1
DDT	0	0	1	1	2	1	0	1	0	1	0
E2F-DP	3	3	3	3	3	4	4	4	2	3	4
FHA	8	15	11	14	11	14	10	10	11	8	12
G2-like	2	5	4	13	3	3	3	2	2	2	4
GNAT	27	46	26	36	38	37	37	27	27	27	36
HB-PHD	0	0	0	0	0	1	1	1	0	0	0
HB-other	3	3	2	2	1	7	6	3	3	4	1
HMG	9	10	7	5	9	8	11	9	7	5	8
Jumomji	2	1	1	2	1	0	2	2	2	1	0
LIM	1	1	0	1	1	3	4	2	2	2	1
MADS	1	2	2	0	2	1	1	1	1	1	2
MBF1	1	1	1	1	1	1	1	0	0	0	1
MYB	21	39	21	18	21	26	29	26	26	26	34
Med26	3	4	4	4	4	4	4	2	2	2	3
NF-YA	1	0	1	1	0	1	1	1	1	0	0
Nin-like	4	15	6	8	10	6	6	5	3	5	12
PHD	18	30	18	21	28	16	16	9	9	9	9
PLATZ	1	3	1	2	2	2	1	1	1	1	3
Pseudo ARR-B	2	2	1	1	1	1	0	2	0	1	0
RB	1	1	2	2	1	1	1	1	1	1	1
Rcd1-like	1	2	1	1	1	1	1	1	1	1	1
SAND	2	15	1	2	9	1	2	2	2	3	18

SAP	16	13	10	9	10	21	21	9	10	9	9
SBP	4	24	4	37	21	3	6	0	0	0	23
SET	26	54	32	27	54	42	43	28	29	27	38
SNF2	23	31	30	29	34	29	34	26	26	27	28
SWI/SNF-BAF60b	0	2	2	2	1	2	1	2	0	1	2
SWI/SNF-SWI3	1	1	2	0	1	2	0	2	2	2	1
Sigma70-like	1	1	1	2	1	2	2	1	1	1	1
TALE	0	0	0	0	0	0	0	1	1	1	0
TAZ	0	4	2	1	3	1	0	0	0	0	4
TIG	0	5	1	1	11	17	18	17	17	14	5
TRAF	2	50	9	6	55	4	5	2	2	3	20
TUB	2	3	2	2	3	1	1	1	1	1	3
WOX	2	0	0	0	0	4	1	1	1	1	0
WRKY	2	1	1	1	1	2	2	2	3	2	2
Whirly	1	1	1	1	1	1	1	1	1	1	1
YABBY	0	1	0	0	0	2	3	1	1	1	0
bHLH	1	10	5	2	2	2	2	1	4	1	2
bZIP	5	18	25	10	13	10	10	7	4	6	15
mTERF	3	7	1	4	7	4	4	1	4	3	8

Supplementary Table 2. Significantly over and under represented transcription associated proteins. P-values of significantly over- (1) and under- (-1) represented transcription factor families in multicellular algae (*Gonium* and *Volvox*) compared to unicellular green algae are included. Significance was determined using a conservative G test of independence with Williams correction.

Transcription Factor	P Value	Over (1) and Under (-1) Representation in Multicellular Algae
ARID	4.37E-02	-1
C2H2	1.63E-06	-1
C3H	1.05E-02	-1
FHA	3.15E-02	-1
GNAT	4.11E-03	-1
HB-other	1.31E-03	-1
MYB	2.50E-03	-1
NF-YA	4.61E-02	-1
PHD	3.87E-02	-1
SAND	9.92E-04	1
SAP	2.65E-04	-1
SBP	2.54E-02	1
SNF2	2.63E-03	-1
TIG	4.01E-03	-1
TRAF	3.18E-08	1
WOX	1.57E-02	-1
YABBY	2.23E-02	-1

Supplementary Table 3. Abundance of Pfam domain outliers. The abundance of previously identified Pfam domain outliers[7] is included for *Chlamydomonas*, *Gonium* and *Volvox* both versions 1 and 2.

	Histones	Ankyrin repeats	Cysteine protease	Gametolysin	Leucine-rich repeat
Pfam Domain	PF00125	PF00023	PF00112	PF05548	PF00560
Chlamydomonas	124	80	24	50	36
Gonium	134	143	18	41	21
Volvox version 1	56	31	16	109	23
Volvox version 2	53	48	16	60	17

Supplementary Table 4. Pfam domains correlating with the evolution of multicellularity. In the table, the Pfam domain number, Pfam domain name, *Gonium* and *Volvox* version 1 protein IDs, the E-value of *Volvox* hit using *Gonium* as a query sequence, and Pfam abstract/description, are given.

Pfam Domain	Domain Name	Gonium ID(s)	Volvox v1 JGI protein ID(s)	Pfam E-value of Gonium Hit	Pfam E-value of Volvox Hit	E-value of Reciprocal Hits	Pfam Abstract/Description
PF00331	Glycosyl hydrolase family 10	scaffold00046.g249.t1, scaffold00255.g643.t1	94058|PACid:18008356, 100182|PACid:17997083	4.1E-38, 2.5E-27	9.2E-42, 8.6E-39	0, 0, 2E-168, 6E-164	Glycoside hydrolases are a widespread group of enzymes that hydrolyse the glycosidic bond between two or more carbohydrates, or between a carbohydrate and a non-carbohydrate moiety.
PF02721	DUF223	scaffold00016.g684.t1	105399|PACid:17999423	8.90E-07	3.30E-05	0	No Pfam abstract.
PF07221	GlcNAc 2-epim	scaffold00038.g338.t1	86621|PACid:18008339	7.40E-06	3.70E-16	2E-171	This family contains a number of eukaryotic and bacterial N-acylglucosamine 2-epimerase (GlcNAc 2-epimerase) enzymes approximately 500 residues long. This coverts N-acul-D-glucasamine to N-acyl-D-mannosamine.
PF08167	RIX1	scaffold00003.g46.t1	88848|PACid:18004498	6.30E-08	2.60E-06	0	Rix1 is a nucleoplasmic particle involved in rRNA processing/ribosome assembly. It associates with two other proteins, Ipi1 and Ipi3, to form the RIX1 complex that allows Rea1- the AAA ATPase- to associate with the 60S subunit.
PF10022	DUF2264	scaffold00302.g823.t1	90361|PACid:17995048	1.10E-77	1.90E-83	1E-93	Members of this family of hypothetical bacterial proteins have no known function.
PF10049	DUF2283	scaffold00035.g889.t1	95550|PACid:18004197	1.00E-05	1.20E-05	1E-07	Members of this family of hypothetical bacterial proteins have no known function.
PF13402	Peptidase M60	scaffold00055.g330.t1	84647|PACid:18008584, 84573|PACid:17996278, 83195|PACid:18002318	1.30E-63	1.8E-67, 9.9E-65, 6.3E-65	1E-148, 1E-148, 4E-131	This family of peptidases contains a zinc metallopeptidase motif (HEXXHXE) and possesses mucinase activity. It inclues the viral enhancins as well as enhancin-like peptidases from bacterial species.
PF14252	DUF4347	scaffold00022.g783.t1	89515|PACid:18001700	6.80E-07	1.20E-07	0	This domain family is found in bacteria and eukaryotes, and is approximately 160 amino acids in length. There are two completely conserved residues that may be functionally important.
PF14924	DUF4497	scaffold00001.g16.t1	95994|PACid:17996519	3.20E-05	3.50E-06	1E-84	This domain family is found in eukarytoes, and is typically between 107 and 123 amino acids in length. There are two completely conserved G residues that may be functionally important.

Supplementary Table 5. Evolution of OrthoMCL clusters in the green algae using *Volvox* version 1 and symmetric Wagner parsimony. The first section of the table includes the number of predicted genes and gene families at each terminal (species) and internal (ancestor) node. The second section of the table includes the number of predicted gains, losses, and expansions of genes and gene families. Predictions were made using symmetric Wagner parsimony (each gene family may be gained or expanded multiple times and the gain penalty is equal to the loss penalty).

Number at node		
Node	Genes	Families
Otaur	7725	7420
Oluci	7796	7214
Osp	7492	7227
Bpras	7919	7387
Mpusi299	10103	9555
Mpusi1545	10660	10126
Cvari	9791	8455
Csube	9629	8425
Crein	17737	14786
Gpect	17984	14551
Vcv1	14542	12201
Oluci Osp	6816	6707
Ostreococcus	6499	6403
Ostreo Bathy	5616	5533
Micromonas	7491	7347
Prasinophytes	5424	5343
Trebuxiophytes	5498	5328
Multicelluar	4575	4496
Volvocales	4783	4698
Chlorophytes	5033	4946
Green Algae	4141	4077

Change along branch					
Node	Gene Gain	Family Gain	Family Expansions	Gene Loss	Family Loss
Otaur	1528	1318	69	302	301
Oluci	1094	617	408	114	110
Osp	898	734	62	222	214
Bpras	2500	2047	77	197	193
Mpusi299	2716	2311	134	104	103
Mpusi1545	3281	2889	120	112	110
Cvari	4657	3480	228	364	353
Csube	4515	3474	214	384	377
Crein	12954	10088	226	0	0
Gpect	13409	10055	267	0	0
Vcv1	14542	12201	0	4575	4496
Oluci Osp	347	332	10	30	28
Ostreococcus	1057	1040	13	174	170
Ostreo Bathy	494	484	6	302	294
Micromonas	2119	2052	46	52	48
Prasinophytes	1283	1266	14	0	0
Trebuxiophytes	606	518	46	141	136
Multicellular Volvocales	0	0	0	208	202
Volvocales	0	0	0	250	248
Chlorophytes	892	869	10	0	0

Supplementary Table 6. Evolution of OrthoMCL clusters in the green algae using *Volvox* version 1 and asymmetric Wagner parsimony. The first section of the table includes the number of predicted genes and gene families at each terminal (species) and internal (ancestor) node. The second section of the table includes the number of predicted gains, losses, and expansions of genes and gene families. Predictions were made using asymmetric Wagner parsimony (each gene family may be gained or expanded multiple times and the gain penalty is two times higher than the loss penalty).

Number at node		
Node	Genes	Families
Otaur	7725	7420
Oluci	7796	7214
Osp	7492	7226
Bpras	7919	7386
Mpusi299	10103	9548
Mpusi1545	10660	10112
Cvari	9791	8463
Csube	9629	8427
Crein	17737	14837
Gpect	17984	14650
Vcart	14971	13146
Oluci Osp	7308	7157
Ostreococcus	7147	7000
Ostreo Bathy	6681	6539
Micromonas	8328	8135
Prasinophytes	7323	7149
Trebuxiophytes	6924	6619
Multicelluar	10521	9841
Volvocales	10330	9651
Chlorophytes	7084	6807
Green Algae	9123	8793

Change along branch					
Node	Gene Gain	Family Gain	Family Expansions	Gene Loss	Family Loss
Otaur	1120	941	67	542	521
Oluci	841	395	401	353	338
Osp	660	507	65	476	438
Bpras	2022	1609	62	784	762
Mpusi299	2218	1837	125	443	424
Mpusi1545	2937	2557	122	605	580
Cvari	3911	2805	235	1044	961
Csube	3842	2874	209	1137	1066
Crein	7602	5357	326	195	171
Gpect	8211	5473	425	748	664
Vcart	6243	4804	211	1793	1499
Oluci Osp	242	232	7	81	75
Ostreococcus	908	880	21	442	419
Ostreo Bathy	264	256	4	906	866
Micromonas	1224	1190	27	219	204
Prasinophytes	0	0	0	1800	1644
Trebuxiophytes	380	315	34	540	503
Multicellular Volvocales	447	396	40	256	206
Volvocales	3918	3504	98	672	660
Chlorophytes	0	0	0	2039	1986

Supplementary Table 7. Evolution of OrthoMCL clusters in the green algae using *Volvox* version 2 and symmetric Wagner parsimony. The first section of the table includes the number of predicted genes and gene families at each terminal (species) and internal (ancestor) node. The second section of the table includes the number of predicted gains, losses, and expansions of genes and gene families. Predictions were made using symmetric Wagner parsimony (each gene family may be gained or expanded multiple times and the gain penalty is equal to the loss penalty).

Number at node		
Node	Genes	Families
Otaur	7725	7420
Oluci	7796	7214
Osp	7492	7226
Bpras	7919	7386
Mpusi299	10103	9548
Mpusi1545	10660	10112
Cvari	9791	8463
Csube	9629	8427
Crein	17737	14837
Gpect	17984	14650
Vcart	14971	13146
Oluci Osp	6814	6705
Ostreococcus	6493	6397
Ostreo Bathy	5611	5528
Micromonas	7500	7347
Prasinophytes	5438	5355
Trebuxiophytes	5514	5344
Multicelluar	8587	8258
Volvocales	8149	7885
Chlorophytes	5115	5017
Green Algae	4142	4076

Change along branch					
Node	Gene Gain	Family Gain	Family Expansions	Gene Loss	Family Loss
Otaur	1531	1321	69	299	298
Oluci	1098	621	409	116	112
Osp	897	733	85	219	212
Bpras	2502	2049	76	194	191
Mpusi299	2710	2306	133	107	105
Mpusi1545	3273	2876	125	113	111
Cvari	4657	3487	227	380	368
Csube	4506	3467	220	391	384
Crein	9621	6984	362	33	32
Gpect	9590	6571	409	193	179
Vcart	6798	5289	238	414	401
Oluci Osp	351	336	10	30	28
Ostreococcus	1062	1044	14	180	175
Ostreo Bathy	488	481	4	315	308
Micromonas	2117	2043	51	55	51
Prasinophytes	1296	1279	13	0	0
Trebuxiophytes	548	470	40	149	143
Multicellular Volvocales	512	446	53	74	73
Volvocales	3258	3090	60	224	222
Chlorophytes	973	941	15	0	0

Supplementary Table 8. Evolution of OrthoMCL clusters in the green algae using *Volvox* version 2 and asymmetric Wagner parsimony. The first section of the table includes the number of predicted genes and gene families at each terminal (species) and internal (ancestor) node. The second section of the table includes the number of predicted gains, losses, and expansions of genes and gene families. Predictions were made using asymmetric Wagner parsimony (each gene family may be gained or expanded multiple times and the gain penalty is two times higher than the loss penalty).

Number at node		
Node	Genes	Families
Otaur	7725	7420
Oluci	7796	7214
Osp	7492	7226
Bpras	7919	7386
Mpusi299	10103	9548
Mpusi1545	10660	10112
Cvari	9791	8463
Csube	9629	8427
Crein	17737	14837
Gpect	17984	14650
Vcart	14971	13146
Oluci Osp	7308	7157
Ostreococcus	7147	7000
Ostreo Bathy	6681	6539
Micromonas	8328	8135
Prasinophytes	7323	7149
Trebuxiophytes	6924	6619
Multicelluar	10521	9841
Volvocales	10330	9651
Chlorophytes	7084	6807
Green Algae	9123	8793

Change along branch					
Node	Gene Gain	Family Gain	Family Expansions	Gene Loss	Family Loss
Otaur	1120	941	67	542	521
Oluci	841	395	401	353	338
Osp	660	507	85	476	438
Bpras	2022	1609	62	784	762
Mpusi299	2218	1837	125	443	424
Mpusi1545	2937	2557	122	605	580
Cvari	3911	2805	235	1044	961
Csube	3842	2874	209	1137	1066
Crein	7602	5357	326	195	171
Gpect	8211	5473	425	748	664
Vcart	6243	4804	211	1793	1499
Oluci Osp	242	232	7	81	75
Ostreococcus	908	880	21	442	419
Ostreo Bathy	264	256	4	906	866
Micromonas	1224	1190	27	219	204
Prasinophytes	0	0	0	1800	1644
Trebuxiophytes	380	315	34	540	503
Multicellular Volvocales	447	396	40	256	206
Volvocales	3918	3504	98	672	660
Chlorophytes	0	0	0	2039	1986

Supplementary Table 9. Cyclin Dependent Kinase (CDK) and Cyclin protein motifs. In the table, the protein name (*Chlamydomonas*/*Gonium*/*Volvox*), protein ID and signature motif for each protein is shown for *Chlamydomonas*, *Gonium*, and *Volvox.* Dashes in the protein name and blank cells indicate absence.

Protein name Cr/Gp/Vc	Chlamydomonas v4 ID	Chlamydomonas v5.0 ID	Gonium ID	Volvox v1 ID	Volvox v2 ID	Chlamydomonas Motif	Gonium Motif	Volvox Motif
CDKA1/CDKA1/cdka1	127285	Au9.Cre10.g465900	scaffold00047.g387.t1	127504	Vocar20015085m	PSTAIRE	PSTAIRE	PSTAIRE
CDKB1/CDKB1/cdkb1	59842	Au9.Cre08.g372550	scaffold00079.g124.t1	103386	Vocar20013545m	PSTTLRE	PSTTLRE	PSTTLRE
CDKC1/CDKC1/cdkc1	148395	Au9.Cre08.g385850	scaffold00087.g425.t1	82776	Vocar20004488m	PITAIRE	PITAIRE	PITAIRE
CDKD1/CDKD1/cdkd1	137457	Au9.Cre09.g388000	scaffold00005.g340.t1	65162	Vocar20003575m	DPTALRE	DPTALRE	DPTALRE
CDKE1/CDKE1/cdke1	120881	Au9.Cre04.g213850	scaffold00054.g202.t1	68336	Vocar20002074m	SPTAIRE	SPTAIRE	SPTAIRE
CDKG1/CDKG1/cdkg1	126776	Au9.Cre06.g271100	scaffold00010.g1058.t1	127266	Vocar20002754m	SDSTIRE	SDSTIRE	SDSTIRE
CDKG2/-/cdkg2	139908	Au9.Cre17.g742250		127318				
CDKH1/CDKH1/cdkh1	153970	Au9.Cre07.g355400	scaffold00006.g738.t1	83876	Vocar20006848m	PVTSIRE	PVTSIRE	PVTSIRE
CDKI1/CDKI1/cdki1	195781	Au9.Cre12.g494500	scaffold00019.g245.t1	119542	Vocar20013243m	PDVVVRE	PDVVIRE	PDVVVRE
CYCD1/CYCD1.1/cycd1.1	195780	Au9.Cre11.g467772	scaffold00047.g299.t1	127281	Vocar20010063m	LICTE		LTCTE
-/CYCD1.2/cycd1.2			scaffold00047.g300.t1	127284	Vocar20010067m		LLCDE	
-/CYCD1.3/cycd1.3			scaffold00047.g301.t1	127282	Vocar20010127m			
-/CYCD1.4/cycd1.4			scaffold00100.g16.t1	127283	Vocar20013188m		LLCTE	LLCTE
CYCD2/CYCD2/cycd2	191762	Au9.Cre06.g289750	scaffold00041.g668.t1	127277	Vocar20013437m	LQCDE	LECEE	LICEE
CYCD3/CYCD3/cycd3	206110	Au9.Cre06.g298750	scaffold00044.g48.t1	127287	Vocar20007422m	LFCGE	LECED	LHCED
CYCD4/CYCD4/cycd4	206166	Au9.Cre06.g259500		127321	Vocar20007145m	LDCTE		LECSE
-/CYCD5/-			scaffold00011.g188.t1					

Supplementary Table 10. Summary statistics for Pfam domain and transcription factor analyses for *Chlamydomonas* (version 5.3), *Gonium*, and *Volvox* (version 1 and version 2). Number of transcription factors and Pfam domains (both unique and total) have been scaled by the number of protein coding loci.

Characteristic	Chlamydomonas v5.3	Gonium	Volvox v1	Volvox v2
# Unique trxn factors	49	50	46	44
Scaled # unique trxn factors	0.00276	0.00278	0.00316	0.00294
# Total trxn factors	506	432	383	311
Scaled # total trxn factors	0.02853	0.02402	0.02634	0.02077
# Unique Pfam domains	3482	3340	3269	2495
Scaled # unique Pfam domains	0.19631	0.18572	0.22480	0.16666
# Total Pfam domains	16200	15786	13160	7795
Scaled # total Pfam domains	0.91334	0.87778	0.90496	0.52067

Supplementary Table 11. Summary of genome wide dN/dS values including *Chlamydomonas* version 5.3, *Gonium*, and *Volvox* version 2. Values in the table are averages across 6,221 genes (those for which there is 1:1:1 orthology, determined using OrthoMCL, for *Chlamydomonas*, *Gonium* and *Volvox*).

	Chlamydomonas versus *Gonium*	*Chlamydomonas* versus *Volvox*	*Gonium* versus *Volvox*
dN	0.2904	0.2668	0.2695
dS	0.7671	1.2352	1.2246
dN/dS	0.3484	0.2426	0.2249

Supplementary Table 12. dN/dS values above one for pairwise comparison between *Chlamydomonas* version 5.3 and *Gonium*. dN, dS, and dN/dS values are shown.

Chlamydomonas v5.3 ID	Gonium ID	dN Value	dS Value	dN/dS Value
Cre12.g534400	scaffold00013.g826	0.3927	0.8166	2.6694
Cre12.g495600	scaffold00267.g692	0.4578	1.6398	1.8295
Cre12.g552400	scaffold00021.g760	0.4790	1.0321	1.7321
g6799	scaffold00041.g736	1.3361	0.7419	1.7291
g14374	scaffold02017.g1037	1.8537	0.8513	1.7124
g7330	scaffold00099.g826		0.6790	1.6502
Cre06.g285200.1	scaffold00642.g752	0.2621	1.5549	1.6464
Cre12.g539000	scaffold00124.g485	0.0804	0.7123	1.6235
g6813	scaffold00005.g107	0.4550	0.1472	1.5564
Cre03.g150050	scaffold00026.g468	0.6515	1.1086	1.5195
Cre07.g329950	scaffold00154.g73	0.9444	0.2667	1.4117
Cre06.g307050	scaffold00008.g108	0.6682	1.0475	1.3920
Cre12.g543550.1	scaffold00007.g1225	0.8568	1.0482	1.3276
Cre14.g628000	scaffold00023.g98	0.3509	1.0836	1.2751
Cre16.g687400	scaffold00004.g922	0.3448	1.2286	1.2702
g4498	scaffold00109.g197	1.6988	1.6347	1.2377
Cre14.g632400	scaffold00039.g382	0.8501	0.7139	1.2287
g8447	scaffold00033.g672	1.2105	1.2631	1.2236
g11471	scaffold01642.g773	0.3295	3.1872	1.1993
g16787	scaffold00002.g1380	0.1460	0.9058	1.1628
Cre12.g523900	scaffold00008.g124	0.2458	1.2395	1.1518
g3099	scaffold00026.g561	0.7680	0.5967	1.1455
Cre10.g440000	scaffold00038.g288	1.2784	1.2265	1.1415
Cre12.g559450	scaffold01180.g441	0.3366	0.5176	1.1316
g17444	scaffold00011.g19	0.3295	0.7829	1.1224
Cre02.g075600	scaffold00279.g736	0.3243	0.7388	1.1050
Cre06.g254100	scaffold01894.g949	0.4675	3.0000	1.1040
Cre13.g575600	scaffold00022.g850	0.8726	0.7580	1.1003
g5122	scaffold00045.g182	0.9887	0.7208	1.0996
Cre17.g736100	scaffold00011.g216	1.0802	1.0713	1.0851
Cre02.g119800	scaffold00005.g397	1.1264	1.0219	1.0759
g5204	scaffold00046.g235	0.1057	0.7894	1.0731
g4911	Minus_MT.g1294	1.2588	1.1172	1.0690
Cre07.g321100	scaffold00001.g197	0.2717	1.2529	1.0585
Cre13.g603450	scaffold00008.g2	0.8819	0.7964	1.0571
Cre02.g097200	scaffold00028.g751	0.5771	0.6880	1.0551
Cre12.g560100	scaffold00075.g725	0.3289	1.3798	1.0533
g18018	scaffold00009.g730	1.5186	0.7987	1.0523
Cre11.g477750	scaffold01025.g295	1.4092	1.4884	1.0512
Cre06.g281286	scaffold00002.g1120	1.1048	1.2182	1.0469

Cre11.g476600	scaffold00002.g1339	0.8254	0.7835	1.0360
g18292	scaffold00055.g258	0.8562	0.8136	1.0179
g5045	scaffold00016.g602	0.4435	0.6162	1.0162
Cre17.g698750	scaffold00024.g258	0.5210	0.6950	1.0157

Supplementary Table 13. dN/dS values above one for pairwise comparison between *Chlamydomonas* version 5.3 and *Volvox* version 2. dN, dS, and dN/dS values are shown.

Chlamydomonas v5.3 ID	Volvox v2 ID	dN Value	dS Value	dN/dS Value
g14374	Vocar20008877m	1.4578	0.8551	2.1678
Cre12.g501050	Vocar20014074m	0.3010	1.0460	1.7207
Cre12.g499100	Vocar20004852m	0.4008	0.9080	1.7039
g6799	Vocar20008488m	1.2828	0.8445	1.5821
Cre06.g263800	Vocar20002515m	0.2055	0.9374	1.5316
g4370	Vocar20007162m	0.4672	1.2029	1.4966
Cre10.g448350	Vocar20007005m	0.3373	1.1338	1.3617
Cre06.g257250	Vocar20002903m	0.0863	1.1797	1.3119
Cre04.g228550	Vocar20008205m	0.4121	1.3922	1.2701
g15195	Vocar20001657m	0.4072	1.3737	1.2352
Cre13.g591700	Vocar20013486m	0.1266	1.2392	1.2301
g6813	Vocar20004950m	0.2291	0.3748	1.2140
Cre03.g180200	Vocar20007821m	0.3670	1.2034	1.1997
Cre12.g524750	Vocar20011203m	0.5200	1.6713	1.1949
g4498	Vocar20007263m	2.0232	1.5619	1.0876
Cre02.g112950	Vocar20008811m	1.2350	1.4191	1.0710
Cre12.g537550	Vocar20011135m	0.1134	1.2405	1.0672
Cre12.g535400	Vocar20011119m	0.5006	1.4078	1.0628
Cre08.g369250	Vocar20002365m	0.4720	1.1201	1.0576
g18399	Vocar20007497m	0.1695	1.5023	1.0402
Cre10.g440000	Vocar20008764m	1.4000	1.2439	1.0277
Cre05.g236100	Vocar20012914m	0.4690	1.5144	1.0204
Cre10.g421350	Vocar20000960m	0.3262	1.5765	1.0163
g18018	Vocar20003971m	0.8405	1.5043	1.0095
g5121	Vocar20012593m	0.3033	1.4703	1.0090
Cre12.g525600	Vocar20003754m	0.4779	1.1011	1.0067

Supplementary Table 14. dN/dS values above one for pairwise comparison between *Gonium* and *Volvox* version 2. dN, dS, and dN/dS values are shown.

Gonium ID	Volvox v2 ID	dN Value	dS Value	dN/dS Value
scaffold01894.g949	Vocar20003473m	4.2729	1.5732	2.7161
scaffold00642.g752	Vocar20010146m	3.0891	1.4913	2.0714
scaffold00008.g108	Vocar20007054m	1.9954	1.1277	1.7694
scaffold00466.g382	Vocar20010734m	2.7373	1.8363	1.4907
scaffold00037.g139	Vocar20004852m	1.4191	0.9646	1.4712
scaffold01642.g773	Vocar20007459m	2.4742	1.6892	1.4647
scaffold00001.g197	Vocar20003181m	1.3595	1.0239	1.3278
scaffold00030.g217	Vocar20014074m	1.5950	1.2424	1.2838
scaffold00064.g146	Vocar20007162m	1.6257	1.2724	1.2777
scaffold00005.g1	Vocar20002903m	1.5566	1.2286	1.2670
scaffold00267.g692	Vocar20013346m	3.0000	2.4090	1.2453
scaffold01600.g746	Vocar20000707m	3.0000	2.4474	1.2258
scaffold00023.g98	Vocar20001559m	1.4542	1.1929	1.2190
scaffold00819.g48	Vocar20001657m	1.2877	1.0917	1.1795
scaffold00124.g485	Vocar20011145m	1.1029	0.9489	1.1623
scaffold00017.g783	Vocar20013486m	1.5844	1.3761	1.1514
scaffold00010.g870	Vocar20002515m	1.1988	1.0448	1.1474
scaffold00021.g760	Vocar20006753m	1.9151	1.6859	1.1360
scaffold00026.g468	Vocar20000660m	1.7048	1.5109	1.1283
scaffold00005.g107	Vocar20004950m	0.3643	0.3259	1.1178
scaffold00007.g1151	Vocar20006412m	1.6628	1.5716	1.0580
scaffold00033.g672	Vocar20012846m	1.5073	1.4258	1.0572
scaffold00002.g1380	Vocar20007051m	1.0452	1.0277	1.0170
scaffold00008.g163	Vocar20003754m	1.0224	1.0140	1.0083
scaffold00624.g719	Vocar20008205m	1.4007	1.3964	1.0031

Supplementary Materials and Methods

Strain and Genome Sequencing

The *Gonium pectorale* strain K3-F3-4 (mating type minus, NIES-2863 from the Microbial Culture Collection at National Institute for Environmental Studies, Tsukuba, Japan, http://mcc.nies.go.jp/) was used for genome sequencing. *Gonium* was grown in 200-300 mL VTAC media at 20C with a 14:10 hour light-dark cycle using cool-white fluorescent lights (165-175 $\mu mol*m^{-2}*s^{-1}$).

For next-generation sequencing and construction of a fosmid library, total DNA was extracted[8]. Sequencing libraries were prepared using the GS FLX Titanium Rapid Library Preparation Kit (F. Hoffmann-La Roche, Basel, Switzerland) and the TruSeq DNA Sample Prep Kit (Illumina Inc., San Diego, CA, USA) and were run on both GS FLX (F. Hoffmann-La Roche) and MiSeq (Illumina Inc.) machines. Newbler v2.6 was used to assemble the GS FLX reads. A fosmid library was constructed in-house using vector pKS300. The fosmid library (23,424 clones) and BAC library (18,048 clones, Genome Institute (CUGI), Clemson Univ., Clemson, SC, USA) were end sequenced using a BigDye terminator kit v3 (Life Technologies, Carlsbad, California, USA) analyzed on automated ABI3730 capillary sequencers (Life Technologies). The resulting *Gonium* assembly is of relatively high quality (Supplementary Figures 9-11; Table 1).

Evidence Based Gene Prediction

Introns hint file generation was done through a two-step, iterative mapping approach using Bowtie/Tophat command lines and custom Perl scripts written by Mario Stanke as part of AUGUSTUS[9], (available at: http://bioinf.uni-greifswald.de/bioinf/wiki/pmwiki.php?n=IncorporatingRNAseq.Tophat). AUGUSTUS version 2.6.1 was selected because its algorithm has been successfully tuned to predict genes in *Chlamydomonas* and *Volvox* genomes, which contain high GC content[9]. Reads were first mapped to the genome assembly with Tophat version 2.0.2[10] and the raw alignments were filtered to create an initial (intron) hints file, which was subsequently provided to AUGUSTUS during gene prediction. An exon-exon junction database was generated from the initial AUGUSTUS prediction via a Perl script, and reads were aligned to this database with Bowtie version 0.12.8[11]. The twice-mapped reads (once to the genome and once to the exon-exon sequences) were then merged, filtered, and a final intron hints file was created. From this the final gene prediction with AUGUSTUS was performed.

Pfam Domain Analysis

Diversity and abundance of Pfam domains was determined for all published green algae genomes. Chlorophyte genomes including *Bathycoccus prasinos*[12], *Chlamydomonas reinhardtii*[13], *Chlorella variabilis*[14], *Coccomyxa subellipsoidea* C-169[15], *Micromonas pusilla* CCMP1545[16], *Micromonas pusilla* RCC299[16], *Ostreococcus tauri*[17], *Ostreococcus lucimarinus*[18], Ostreococcus sp. RCC809 (US Department of Energy, Phytozome), and *Volvox carteri* (both versions 1 and 2)[19] were searched using direct submission of Pfam A and Pfam B domains using Bioperl. Subsequent hits were counted and produced a matrix of Pfam domain diversity and abundance across green algae. Comparing to unicellular Chlorophyte outgroups, there are 210 Pfam domains unique to

the volvocine algae. Of these 210 domains, nine Pfam domains correlate with the origin of multicellularity in the volvocine algae (Figure 2d, Supplementary Table 5). Of these nine Pfam domains, five Pfam domains are "Domain of Unknown Function" (DUF) domains. Of the four annotated domains, two are likely related to metabolic processes; Glycoside hydrolase family 10, where glycoside hydrolases break down carbohydrates, and GlcNAc 2-epim, an enzyme which coverts glucosamine to mannosamine. One annotated Pfam domain (RIX1) is involved in the rRNA processing/ribosome assembly and is thus unlikely to be associated with the evolution of multicellularity. The last annotated Pfam domain (Peptidase M60) is a zinc metallopeptidase, which breaks down mucin (glycosylated proteins). As the extracellular matrix (ECM) of *Volvox* is largely composed of glycosylated proteins[20,21], genes containing this Pfam domain may be involved in breaking down the ECM during reproduction, thus warranting further investigation.

Significant increases or decreases in the number of Pfam domains were determined using a conservative G test of independence with Williams correction[22] to compare the abundance of each Pfam domain in *Gonium* and *Volvox* to unicellular Chlorophyte species, comparing the abundance of each Pfam domain to the total number of genes in each species with $\alpha = 0.05$ (Supplementary Figure 4). A multitude of Pfam A domains are significantly over- (129) or under-represented (394) in colonial/multicellular *Gonium* and *Volvox* compared to unicellular green algae (Supplementary Figure 4; Supplementary Data 1). The observation that Pfam domain innovation is not correlated with the evolution of multicellularity is still robust when using $\alpha = 0.0001$. In this case, 94 Pfam domains are differentially represented, with 43 over-represented and 51 under-represented in colonial/multicellular *Gonium* and *Volvox* compared to unicellular green algae (Supplementary Data 1).

The diversity and abundance of Pfam domains was normalized by the number of gene sequences in each genome and scaled to unity in order visualize in a heatmap. A heatmap of all Pfam A domains (*Volvox carteri* version 1, Supplementary Figure 12; *Volvox carteri* version 2, Supplementary Figure 13) and Pfam B domains (*Volvox carteri* version 1, Supplementary Figure 14; *Volvox carteri* version 2, Supplementary Figure 15) shows overall conservation with relatively few volvocine and colonial/multicellular volvocine innovations. The total number of Pfam domains is included in Supplementary Table 10, and significance of Pfam domain over- and under-representation is included in Supplementary Data 1. When *Volvox carteri* version 2 is included in this analysis, it is apparent that numerous Pfam A domains present across all other green algae, including *Chlamydomonas* and *Gonium*, are absent in *Volvox carteri* (Supplementary Figure 12). While the cause of this phenomenon is unknown, given this peculiarity, much of our further analyses utilized *Volvox* version 1.

Analysis of Transcription Associated Proteins

Transcription associated proteins (TAPs) include transcription factors (TFs, enhance or repress transcription) and transcription regulators (TRs, proteins which indirectly regulate transcription such as scaffold proteins, histone modification or DNA methylation). We combined three TAP classification rules for plants; PlantTFDB[23], PlnTFDB[24], and PlanTAPDB[25] to make a set of classification rules for 96 TAP families. Conflicts between the three sets of rules were manually resolved using the rule that included more genes as transcription associated proteins.

Each transcription family includes at least one, up to three, mandatory domains. Families may include up to six forbidden domains (*i.e.*, a gene *G* cannot be in family *F* if domain *D* is present); not all families have defined forbidden domains. All mandatory and forbidden domains were represented by a full length, global, Hidden Markov Model (HMM). Available HMMs were retrieved from Pfam_ls database[26,27]. When HMMs were not available from the Pfam_ls database, custom HMMs were made using multiple sequence alignments from PlnTFDB[24] and the HMM was calculated using HMMER version 3.0[28] using "hmmbuild" with default parameters and "hmmcalibrate --seed 0".

Gathering cutoff thresholds (GA) for the custom HMMs were set as the lowest score of a true positive hit using a "hmmscan" search against several complete Chlorophyte genomes. Chlorophyte genomes including *Bathycoccus prasinos*[12], *Chlamydomonas reinhardtii*[13], *Chlorella variabilis*[14], *Coccomyxa subellipsoidea* C-169[15], *Micromonas pusilla* CCMP1545[16], *Micromonas pusilla* RCC299[16], *Ostreococcus tauri*[17], *Ostreococcus lucimarinus*[18], Ostreococcus sp. RCC809 (available on the DOE Phytozome website, version 10.1), and *Volvox carteri*[19] were searched using "hmmscan" to search the library of 103 domains against the predicted protein sequences. Analyses were replicated with both *Volvox* version 1 and version 2; however, as results were not qualitatively different, results from version 1 are provided (Supplementary Figure 3). Subsequent hits were classified into a TAP family. Conflicts between multiple TAP families were resolved by assigning the gene to the TAP family with the highest score (Supplementary Table 2).

The total number of transcription factors in colonial/multicellular algae (*Gonium*, 432/17984; *Volvox* version 1, 383/14542; *Volvox* version 2, 311/14971) is lower than a closely related unicellular relative (*Chlamydomonas*, 506/17737). This result is statistically significant (p=0.000064, two-tailed conservative G test of independence with Williams correction, using *Volvox* version 2 data), though this p-value is highly sensitive to *Volvox* version 2 data. When *Volvox* version 1 data is used, the p-value is increased substantially (p=0.02446, two-tailed conservative G test of independence with Williams correction).

Significant increases or decreases for each TAP family were determined using a conservative G test of independence with Williams correction[22] to compare the number of transcription factors in *Gonium* and *Volvox* to unicellular Chlorophyte species with $\alpha = 0.05$ (Figure 2b; Supplementary Table 3). There is a significant reduction of transcription factors in colonial/multicellular *Gonium* and *Volvox* including PHD (chromatin binding), C2H2 (DNA binding transcription factors) and GNAT (acetyltransferase). Significant increases of transcription factor families in *Gonium* and *Volvox* include SAND, (DNA binding), SBP (DNA binding) and TRAF (general transcription factors). The increase in the SAND family is likely an increase throughout the volvocine algae due to the VARL gene family. Each gene in the VARL gene family contains one SAND domain (see Analysis of VARL Genes section). Given relative conservation of the VARL gene family across the volvocine green algae (Figure 5b), this TAP family is unlikely to be associated with the evolution of multicellularity. When a decreased significance value ($\alpha = 0.005$) is used, there is little transcription factor innovation during the evolution of multicellularity (2 transcription factors are over-represented and 7 transcription factors are under-represented in colonial/multicellular *Gonium* and *Volvox* (Supplementary Table 3)).

The number of transcription factors in each TAP family was normalized by the number of gene sequences in each genome and then these data were scaled to unity in order visualize in a heatmap. A heatmap of all transcription associated proteins for all Chlorophyte genomes is shown in Supplementary Figure 3 and the number of transcription factors is included in Supplementary Table 2.

Construction of Protein Families

Protein families were created using OrthoMCL[29] with a variety of inflation values ranging from 1.2 to 4.0 in steps of 0.1 (Supplementary Figures 16-17). This analysis was performed using Chlorophyte genomes available on the DOE JGI Phytozome website, version 10.1 including *Bathycoccus prasinos*[12], *Chlamydomonas reinhardtii*[13], *Chlorella variabilis*[14], *Coccomyxa subellipsoidea* C-169[15], *Micromonas pusilla* CCMP1545[16], *Micromonas pusilla* RCC299[16], *Ostreococcus tauri*[17], *Ostreococcus lucimarinus*[18], *Ostreococcus* sp. RCC809 (available on the DOE Joint Genome Institute website), and *Volvox carteri*[19]. This analysis was repeated for both *Volvox* version 1 and *Volvox* version 2. The inflation value of 1.9 was used for both analyses for consistency and was chosen in order to have relatively large, coarser grained clusters that were robust to higher inflation values (Supplementary Figures 16-19). In order to avoid bias introduced by not including all genes for each species, genes not assigned to a gene family (singletons) were assigned to single gene families and included in all subsequent phylogenetic gene family analyses.

A species tree was calculated by extracting OrthoMCL gene families containing only one copy in each species, for a total of 1,457 genes. The OrthoMCL run with an inflation value of 1.5 was chosen to use larger, coarser grained clusters, thus increasing the likelihood of capturing true 1:1:1 orthologs. This species tree included *Volvox carteri* version 2. These genes were independently aligned using Muscle version 3.8.31[30] and concatenated. A phylogenetic tree was produced using RAxML version 8.0.20[31] using the Protein Gamma model with automatic model selection on a per gene basis via partitions for each protein. A rapid bootstrapping analysis to search for the best-scoring ML tree was run with 100 bootstraps. The resulting species tree is consistent with previous results[16,32,1,33,34] and had 100 bootstrap support at every node (Supplementary Figure 20). This result is also consistent with numerous morphological characteristics supporting a closer relationship of *Gonium* and *Volvox*[35].

Gene family evolution within the Volvocine algae was analyzed using Count version 10.04[36] to perform several parsimony analyses including symmetric Wagner parsimony (each gene family may be gained or expanded multiple times and the gain penalty is equal to the loss penalty) and asymmetric Wagner parsimony (each gene family may be gained or expanded multiple times and the gain penalty is 2 times higher than the loss penalty). This analysis was repeated for both *Volvox* version 1 and version 2 genomes (Supplementary Tables 6-9). All previously mentioned Chlorophyte genomes were included in both analyses and the calculated phylogenetic tree (Supplementary Figure 20) was used to guide gene family evolution. A Dollo parsimony analysis, where only presence or absence, not size, of the family is considered and each gene family may only be gained once, thereby preventing convergent evolution between lineages, was also performed. This analysis included qualitatively more gene/family loss and is not included here.

The results from all four analyses (two *Volvox* versions with two Wagner parsimony analyses each) were qualitatively similar. Aggregate information for lineage-specific gene family changes was collected using Count command line[36] and all analyses suggested that while there is more gene and gene family turnover throughout the evolution of the volvocine algae compared to other green algae, there is relatively little predicted gene family innovation at the origin of multicellularity (Figure 2c; Supplementary Tables 6-9). This is consistent with a relatively short time before the radiation of multicellular volvocine algae (that is, the *Gonium* lineage and *Volvox* lineage speciated quickly after evolving undifferentiated multicellularity). There is substantial lineage specific evolution in the volvocine algae, which may be instead attributed to ecological pressures, if these expanded gene families are adaptive at all.

dN/dS Analysis

During our OrthoMCL construction of protein gene families, we identified 6,154 clusters with exactly one copy in *Chlamydomonas* (version 5.3), *Gonium*, and *Volvox* (version 2). The number of genes from other unicellular (non-*Chlamydomonas*) Chlorophyte species was ignored. This criteria is relatively strict as it does not include any genes with a duplicate in any species (copy number greater than one in any species) or any genes which are not essential (no copy present in any species) resulting in 1:1:1 orthologs. Given the relatively high gene duplication rates in volvocine algae (data not shown), these strict criteria support an interpretation of 1:1:1 orthology. Genome wide pairwise comparisons of dN, dS, and dN/dS were calculated (Supplementary Figure 21; Supplementary Table 11) using PAML and codeml (ML analysis[37]) based on nucleotide translation based alignments (proteins were aligned using MUSCLE[30]). These genome wide values are relatively high for genome wide comparisons (dN: 0.2695-0.2904, dS: 0.7671-1.2352, dN/dS: 0.2249-0.3484; Supplementary Table 11), which is likely explained by the relatively long divergence time (*Chlamydomonas* lineage from *Gonium*/*Volvox* lineage is ~250 million years, *Gonium* lineage from *Volvox* lineage is ~210 million years) within the volvocine algae[38]. While dS values are approaching saturation, artificially increasing dN/dS values, these estimates of dN, dS, and dN/dS are reliable for the purposes of identifying putative targets of selection (Supplementary Tables 11-14).

Using pairwise comparisons between these three putative orthologs, we identified relatively few genes with high dN/dS (dN/dS>1). For the *Chlamydomonas* versus *Gonium* comparison, 44 genes show dN/dS greater than one (Supplementary Table 12). For the *Chlamydomonas* versus *Volvox* comparison, 26 genes show dN/dS greater than one (Supplementary Table 13). For the *Gonium* versus *Volvox* comparison, 25 genes show dN/dS greater than one (Supplementary Table 14). This is a relatively small number of genes showing strong positive selection (dN/dS: 1.0031-2.7161; Supplementary Tables 11-14), compared to the number of orthologs studied (6,154 genes) or the number of genes in each genome (approximately 15,000-17,000 genes) suggesting that relatively few genes experienced strong positive selection throughout the volvocine algae, including genes beneficial for the evolution of multicellularity and adaptations to environmental conditions.

Prediction of Lineage-specific Genes

To understand when genes appear in the evolution of the volvocine algae, we determined the evolutionary birth date of every gene in the genomes of *Chlamydomonas*, *Gonium*, and *Volvox* using genomic phylostratigraphy[39]. This method places a gene in evolutionary age categories, or phylostrata (PS), depending on the presence of homologs in other species.

The phylostratigraphy method[39] assumes Dollo's parsimony (*i.e.*, it is more likely that a gene observed in two distant clades was present in the common ancestor, and multiple independent gains are not possible). This provides an entry point for testing evolutionary hypotheses related to the age of genes and to quantify how much gene-level innovation has occurred along each phylogenetic branch. Old genes are classified in low phylostrata (present in distant species, PS1-PS7) and young genes are classified in higher phylostrata (e.g., genus- or species- specific genes, PS8-PS9). The resolution of each phylostratum strictly depends on the availability of reliable outgroups (the availability of reliable genomic outgroups is relatively low in Chlorophyte algae). The phylogenetic classes were defined from those in each NCBI Taxonomy entry for *Chlamydomonas*, *Gonium*, and *Volvox*, resulting in nine expected phylostrata for each species. All proteins were subjected to a BLASTP[40] search with an E-value threshold of 0.001 against the NCBI nr database. Placement in phylostrata was derived from the taxonomic information of these hits for each protein, using the most distant hit, and following Dollo's parsimony. Additionally, two filtering steps were included. Proteins with identical sequence to other proteins, proteins with illegal amino acid characters or stop codons within the protein sequence, proteins shorter than 60 amino acids, and proteins longer than 4,000 amino acids were excluded from analysis. Furthermore, to correct for absence of the newly annotated sequences of the algal genomes in this study from the NCBI nr database, BLASTP analyses were performed between all three species, correcting the database size to match that of the NCBI nr.

Genomes were arranged by their evolutionary age of birth and assigned a phylostratum identity. A surprisingly high number of genes are species-specific (PS9; Figure 2a). There are more than 1,000 new genes in each of the three genomes (*Chlamydomonas*, 2748; *Gonium*, 1334; *Volvox*, 2887), which is consistent with the long divergence time between these three lineages. In contrast, far fewer genes exist in the PS7 phylostratum (Figure 2a), corresponding to the age when multicellularity evolved. Relatively few new genes appeared during the evolution of multicellularity (PS7). At PS7 relatively few new genes appeared in *Gonium*: maximum of n=344, *Volvox*: maximum of n=188), which may play a role in the evolution of multicellularity in the volvocine algae. Compared to gene innovation at the origin of the *Chlamydomonadales* (PS6) or species-specific innovation (PS9; Figure 2a), there are relatively few genes that coincide with the evolution of multicellularity. This suggests gene innovation ancestral to the *Chlamydomonadales* that resulted in a predisposition for the evolution of multicellularity, but may also be related to environmental adaptation.

Analysis of Cell Cycle Genes

In order to investigate cell cycle regulation during the evolution of multicellularity, we annotated cell cycle regulatory genes as previously done for *Chlamydomonas*[41]. As in *Chlamydomonas*, all of the cell cycle regulatory genes in *Gonium* and *Volvox* are single copy, with the exception of the cyclin AB genes in

Gonium (three copies) and cyclin D1 genes which are expanded in both *Gonium* and *Volvox*. Both *Gonium* and *Volvox* have four cyclin D1 genes (Figure 3c), whereas *Chlamydomonas* has only one cyclin D1 gene (Figure 3c; Supplementary Tables 10, Supplementary Data 4).

Briefly, identification of the cell cycle regulatory genes in *Gonium* involved multiple steps, blasting *Chlamydomonas* v.5.3 cell cycle regulatory protein sequences against *Gonium*. For all blast analyses, an E-value cut off of 10^{-4} was used. *Gonium* scaffolds and protein models with the most hits and lowest E-value cut off (for example, multiple hits to scaffold 47 all with E-values less than 10^{-4}) were identified as the *Gonium* cell cycle regulatory genes, in this example, cyclin D genes.

Specifically, the *Gonium* scaffolds on which the target genes were located were first identified using a "tblastn" search using the *Chlamydomonas* gene sequence as the query and the *Gonium* genome as the subject. A custom Perl script was used to identify the nucleotide sequences of the top hit *Gonium* scaffolds. Next, protein sequences for these top hit *Gonium* scaffolds were obtained from the *Gonium* protein models. Finally, a "blastp" search was performed with the *Chlamydomonas* cell cycle regulatory protein against the *Gonium* protein sequences. For cyclin genes in *Gonium*, gene models were manually inspected and modified.

Cell cycle regulatory gene motifs were identified (Supplementary Table 10[41]). All *Gonium* cyclin dependent kinases (CDKs) have the same CDK motif as *Chlamydomonas*, except for *Gonium* CDKI, which has the motif PDVVIRE where *Chlamydomonas* and *Volvox* both have CDKI motif of PDVVVRE (Supplementary Table 10). The conserved cyclin motif of LXCXE, where X represents any amino acid, is present in all of *Chlamydomonas* cyclin D genes but is absent in three *Gonium* cyclin D genes (cyclin D1.1, cyclin D1.3, and cyclin D5, an apparent novel *Gonium* cyclin gene) and two *Volvox* cyclin D genes (cyclin D1.2 and cyclin D1.3; Supplementary Table 10). *Gonium* does not appear to have a cyclin D4 gene and instead has a novel cyclin gene, cyclin D5. For the *Gonium* genes that lack the conserved cyclin motif, two of them (cyclin D1.3 and cyclin D5) have a conserved cyclin N terminal domain (determined using NCBI Conserved Domain Search), whereas cyclin D1.1 in *Gonium* does not have any conserved cyclin domains. The cyclin tree (Figure 3c) was created using the standard phylogenetic script described below, with the modification of an automatically determined number of bootstraps[31] resulting in 1,000 bootstraps.

The cyclin tree has relatively few sites available (1,032 distinct alignment patterns) suggesting that *Gonium* cyclin D1 genes are most closely related to each other and all *Volvox* cyclin D1 genes are most closely related to each other (Figure 3c). We investigated the possibility of independent duplications of the cyclin D1 genes in *Gonium* and *Volvox*. Using GenePainter version 2[42], we aligned exon and intron sequences including the frame in which these introns occur. There are five conserved introns, in position and frame, across the cyclin D1, D2, D3, D4, and D5 genes in *Chlamydomonas*, *Gonium*, and *Volvox*, which have been subsequently lost in several apparently gene-specific events (Supplementary Figure 22). Of note is a unique intron that is shared, in position and frame, between cyclin D1.1 in *Gonium* and cyclin D1.3 in *Volvox* (Supplementary Figure 23). Additionally, this shared intron is very near the position of one of the conserved introns across cyclin D genes (Supplementary Figure 23). This

conserved intron has apparently been lost in both cyclin D1.3 in *Volvox* and cyclin D1.1 in *Gonium*.

Given the close proximity in these two introns, we hypothesize this intron is a derived synapomorphy in cyclin D1.1 in *Gonium* and cyclin D1.3 in *Volvox*[43], suggesting that the cyclin D1 expansions in *Gonium* and *Volvox*, relative to *Chlamydomonas*, are not tandem duplications but rather a single event. Our hypothesis suggests that in the common ancestor of *Gonium* and *Volvox*, the cyclin D1 gene was duplicated, and in one gene, a novel intron evolved near the third conserved intron. As often occurs with multiple nearby introns[43], we hypothesize one intron was lost (the conserved intron) and following the speciation of *Gonium* and *Volvox* lineages, this gene became cyclin D1.1 in *Gonium* and cyclin D1.3 in *Volvox*. While this evidence is indicative, it is not conclusive. Further genomic analysis of other volvocine algae (Supplementary Figure 1) would likely resolve the single duplication and independent duplication hypotheses.

A pairwise dN/dS analysis was performed on cell cycle genes for *Chlamydomonas*, *Gonium*, and *Volvox*, following the methods outlined above. When compared between species, most cell cycle regulators appear to be under strong stabilizing selection (consistent with genome wide dN/dS values (Supplementary Figure 21), as expected for core cell machinery such as cell cycle regulators. However, two classes of genes appear to have higher values of pairwise dN/dS, cyclin AB genes (elevated by *Chlamydomonas* and *Gonium* comparisons) and cyclin D genes (Supplementary Figure 8). Given that cyclin AB genes are expanded in *Gonium*, future transcriptomic investigation may illuminate the evolutionary explanation and current function of these genes. For the cyclin D genes, elevated dN/dS values are consistent with positive selection, likely associated with the evolution of multicellularity (Supplementary Figure 8). Future transcriptomic investigation of the expression profiles of these genes promises to inform the evolutionary history and adaptive value of this expansion. Calculation of dN/dS for *RB* was not possible as synonymous sites are at saturation (pairwise dN values for *Chlamydomonas*, *Gonium*, and *Volvox* (both male and female alleles) are 0.269-0.377). This sequence evolution is consistent with the shorter linker region (both are likely the product of rapid evolution at the *RB* locus) and previous observations of rapid evolution in the sex loci[44], further bolstering the argument that *RB* is critical for the evolution of multicellularity.

Analysis of retinoblastoma genes in the volvocine algae revealed relatively rapid evolution in the linker region of the *RB* protein, likely affecting structure and thus function of the protein. The differences in the length of this linker region (Figure 3d) are consistent with other volvocine algae and unicellular relatives[45,46]. The *RB* gene in the *Chlamydomonas* lineage may not represent the ancestral sequence; additional *RB* sequences from many unicellular relatives are necessary before fully reconstructing the evolutionary history of the structure of the *RB* gene in the volvocine algae.

Analysis of VARL genes

The VARL gene family (Volvocine Algal RegA-Like[47]) includes *regA*, which is known to regulate somatic differentiation in *Volvox carteri*[48,49], likely through regulation of nuclear-encoded chloroplast biogenesis genes[48,50–52]. VARL genes contain a single SAND domain. There are 12 (*RLS1*-*RLS12*) VARL genes in *Chlamydomonas reinhardtii* and 14 (*rlsA*-*rlsM*, *regA*) in *Volvox carteri* f. *nagariensis*. In *Volvox*, *rlsA*, *rlsB*, *rlsC*, and *regA* (known as the *regA* cluster) are a tandem duplicated array of 4 genes.

Recombination has resulted in a translocation of this cluster away from *rlsD* in *Volvox carteri*. The *regA* cluster in *Volvox carteri* and *Volvox ferrisii* share conserved protein sequence motifs, synteny, and intron position, suggesting this tandem array arose from duplication of *rlsD*[53]. The ortholog of *rlsD* in *Volvox* is *RLS1* in *Chlamydomonas* and *Gonium*. In *Chlamydomonas*, the *RLS1* gene is known to be up regulated in stressful environments (*i.e.*, light, phosphorus, nitrogen, sulphur depletion), which is consistent with down regulation of reproduction[51,52].

VARL genes, including the *regA* gene cluster and related *RLS/rls* genes, are putative transcription factors known to encode a single DNA-binding SAND domain[47,54] which is approximately 75 amino acids long. Outside of this domain, there is poor conservation among VARL genes with the exception of very short conserved sequences of unknown (if any) function in *rlsA*, *rlsB*, *rlsC*, and *regA*[53]. Therefore, any phylogenetic analyses are necessarily restricted to the short, conserved SAND domain.

In order to identify VARL genes present in *Gonium*, we took all published VARL gene sequences from *Chlamydomonas*, *Volvox carteri* f. *nagariensis*, *Volvox ferrisii*, and *Volvox gigas*[13,47,53,54] and searched both the predicted genes and assembly of *Gonium* using a "blastn" search with an E-value of 1. For hits to the assembly where a previously predicted gene model was not present, models were built manually. The presence of a SAND domain in computationally predicted and manually constructed VARL genes were verified using Pfam version 26.0 and SMART version 7.0[27,55] with an E-value of 10^{-7} and $5*10^{-2}$, respectively. There were two cases (*sc5:g127*, *sc11:g233*) where predicted domains did not pass the SMART threshold but were retained after manual inspection and highly significant (10^{-9}) Pfam E-values. Only one VARL gene (*sc11:g146b*) not predicted was found and subsequently added to the gene models. A total of eight VARL genes were found in *Gonium*.

In order to determine phylogenetic relationship, VARL genes from all available genome sequences (*Chlamydomonas*, *Gonium*, *Volvox carteri* f. *nagariensis*) were aligned using MAFFT version 6.859b with the L-INS-I option[56]. A phylogenetic tree was produced using RAxML version 8.0.20[31] with the Protein Gamma model and automatic model selection. The rapid bootstrapping analysis to search for the best-scoring ML tree was run with 1000 bootstraps.

This phylogenetic tree did not predict a *regA* cluster tandem duplication in *Gonium* because no tandem VARL duplication formed a clade with the *Volvox carteri regA* cluster (Figure 5b). Consistent with previous results[47], *RLS1* in *Gonium* contains only a single intron within the VARL domain, at position 4. As no other VARL genes in *Gonium* contain this architecture, intron position supports the *regA* cluster being absent in *Gonium*. Several other phylogenetic trees including *Volvox gigas*, *Volvox ferrisii*, *Volvox obversus*, and *Volvox africanus*[53], and four possible combinations thereof, were built with the same phylogenetic results (data not shown). In addition, orthology of syntenic genes surrounding *RLS1*, was compared to *Chlamydomonas* and *Volvox*, using a protein search of nearby genes (blastp with an E-value threshold of 10^{-25}). Consistent with the gene phylogeny (Figure 5b), the *regA* tandem duplication is not present in the expected location in *Gonium* (Figure 5a). This tandem duplication is not present in another location, due to recombination, given the syntenic distribution of VARL genes in *Gonium*.

The absence of the *regA* tandem duplication in *Gonium* supports the hypothesis that this cluster evolved after the speciation of *Chlamydomonas* and *Volvox* lineages[53] rather than evolving before this speciation with subsequent loss in the *Chlamydomonas* lineage[47]. It is possible that this cluster is indeed ancestral to the volvocine algae and both the *Chlamydomonas* and *Gonium* lineages lost the *regA* cluster; however, this requires further investigation into the presence/absence of the *regA* cluster in the genomes of other small, colonial volvocine algae. While the volvocine species tree predicts the presence of a regA cluster in small, undifferentiated species such as *Pandorina* and *Volvulina* (Supplementary Figure 1), this remains to be tested.

The presence of the *regA* cluster in divergent *Volvox* species (*Volvox carteri* and *Volvox ferrisii*) and the role *regA* plays in somatic regulation in *V. carteri* suggests that *regA* may also be regulating somatic cells in *V. ferrisii*, which is predicted to have an independent evolution of somatic cells[38]. A third lineage, the genus *Astrephomene*, is also predicted to have independently evolved somatic cells. While somatic cells in *Pleodorina/Volvox* lineages initially evolve in the anterior pole of the colony and are known to provide motility through flagellar beating[57–59], the somatic cells of *Astrephomene* are in the posterior pole and function as a directive "rudder"[60,61]. As *Gonium* and *Astrephomene* are sister lineages, the absence of *regA* in *Gonium* may indicate the absence of *regA* in *Astrephomene* as well. How then are somatic cells in *Astrephomene* regulated? Given the different functions of somatic cells in *Astrephomene* and *Volvox*, these lineages may utilize different genetic mechanisms to regulate somatic cells, thus indicating the possibility of multiple genetic pathways for the evolution of cellular differentiation. If so, these alternate genetic mechanisms may explain the alternate morphology and function. The subsequent evolutionary consequences of these somatic morphologies and functions remains to be explored.

The presence of VARL genes in *Gonium* also helps to reveal previously un-explored orthologous relationships amongst VARL genes in the volvocine algae. Though not strongly supported, there appears to be independent expansions in all three lineages forming *RLS4* and *RLS7* in *Chlamydomonas*, *sc5:g127*, *sc11:146*, *sc11:g146b*, *and sc11:g147* in *Gonium*, and *rlsJ* and *rlsK* in *Volvox*. Given the close synteny of *sc11:146*, *sc11:g146b*, and *sc11:147* in *Gonium*, these appear to be a tandem gene duplication. The intron structure within the VARL domain of these genes is the canonical intron 3 and intron 7[47]. Furthermore, there are at least ten syntenic marker genes around *sc11:g146-147* that are syntenic with *rlsJ* and *rlsK* in *Volvox* and *RLS4* and *RLS7* in *Chlamydomonas* (data not shown), demonstrating this tandem duplication is not a *regA* cluster that has been relocated.

RLS10 in *Chlamydomonas*, *sc788:g9* in *Gonium*, and *rlsL* in *Volvox* also have strong support as conserved orthologs. Lastly, there is strong support for orthologs of *sc11:g233* in *Gonium* and *rlsF* in *Volvox*. While the function of these orthologs, especially the independent expansions, remains unknown, their potential function may prove interesting in understanding the evolutionary history of *regA*, somatic cell evolution, and transcription factors in the volvocine algae.

Analysis of Matrix Metalloprotease Genes

The cell wall in *Chlamydomonas* is made of three layers[62,4]. In multicellular lineages, this cell wall, specifically the innermost layer[63], was coopted to form the extracellular matrix (ECM) with a corresponding expansion of ECM-related proteins

such as pherophorin and matrix metalloprotease (MMP) gene products[19,6]. In *Gonium*, the outer and middle layer of the cell wall surrounds the entire colony while the inner layer surrounds each cell[63,64], suggesting relatively little innovation of the cell wall in early colonial species. It is this inner layer that is greatly expanded in size, producing most of the volume of a *Volvox* colony. Larger volvocine algae, such as *Volvox*, are substantially composed of ECM, which can comprise greater than 95% of colony volume[6]. This ECM not only provides structure[4] but also acts as storage for nutrients such as phosphate and nitrogen[65,66]. MMP genes are composed of a single Pfam metalloprotease domain (Peptidase M11, PF05548) and a hydroxyproline rich repeat. The MMP domain is a metal binding domain which binds zinc or, in *Volvox*, copper ions[67]. In *Chlamydomonas*, MMP genes are thought to degrade and modify the cell wall during growth and gametogenesis, in which cells differentiate into gametes for sexual reproduction[68]. Similarly, in *Volvox*, MMP genes are expressed in somatic cells during sexual reproduction and are thought to degrade the ECM[6].

All previously annotated matrix metalloprotease genes (*Chlamydomonas*, MMP; *Volvox*, VMP) were downloaded from NCBI and used to search the protein models (E-value=10^{-5}) of *Chlamydomonas* (version 5.5), *Gonium*, and *Volvox* (version 1 and version 2). As both *Volvox* version 1 and version 2 gene models were considered, some of the *Volvox* models were redundant (an identical model in both version 1 and version 2). In this case, the naming of the *Volvox* version 2 model was retained. There were multiple cases when a model was present in *Volvox* version 1 but not in version 2 (and vice versa). Whenever a model unique to *Volvox* version 1 was retained, it was ensured that the underlying nucleotide sequence was still present in *Volvox* version 2 (but no model was annotated in that location). This step ensured that no model was included multiple times but that all metalloprotease models were included in the analysis. Remaining gene models were searched for Pfam domains for a Peptidase M11 domain (with an E-value cut off of 10^{-5}) using direct submission to Pfam via a custom Perl script and a metal binding metalloprotease motif using the regular expression [HQ]EXXHXXGXXH[6].

Using the presence of a Peptidase M11 domain as necessary and sufficient criteria for annotation as a MMP gene (Supplementary Data 2), there is an expansion of MMP genes in *Volvox* (98) relative to *Chlamydomonas* (44) and *Gonium* (36). A phylogenetic tree of all Peptidase M11 genes suggests species-specific expansions and innovations (Supplementary Figure 24), which is consistent with the apparent tandem duplication of many of these genes (Supplementary Data 2). Many of these species-specific expansions reconstruct the species tree, while the number of the *Gonium* MMP genes is nearest *Chlamydomonas* rather than *Volvox* (Supplementary Figure 24), suggesting that while some innovation of MMP genes is required for undifferentiated multicellularity; however, most of the genetic innovation regarding MMP expansion occurred during the evolution of large, multicellular organisms such as *Volvox*. Given the multiple, independent evolutions of the *Volvox* morphology[69], understanding when this MMP expansion occurred has important implications on the inevitability and repeatability of the evolution of *Volvox* morphology. We have narrowed this event after the divergence of *Gonium* and *Volvox* lineages, but whether this expansion was a single event (ancestral to *Volvox carteri* and *Volvox ferrisii*; Figure 1, Supplementary Figure 1) or occurred multiple times is unknown.

Using the presence of a Peptidase M11 domain and the metal binding motif ([HQ]EXXHXXGXXH[6]) as stricter criteria for annotation as a MMP gene, approximately 60-70% of genes remain (*Chlamydomonas*, 28; *Gonium*, 22; *Volvox*, 67) and display a phylogenetic pattern consistent with the full MMP gene collection (Supplementary Figure 25). Genes that have a Peptidase M11 domain but not the metal binding metalloprotease motif (*Chlamydomonas*, 16; *Gonium*, 14; *Volvox*, 31) are interleaved between species (Supplementary Figure 26) including some canonically defined matrix metalloprotease genes[19]. This tree suggests species-specific innovation, especially in *Chlamydomonas* and *Volvox*, though well conserved orthologs are also identifiable (Supplementary Figure 24).

Given the syntenic arrangement of many of these species-specific expansions (Supplementary Data 2), it appears that tandem gene duplication is a critical pathway for MMP evolution in the volvocine algae. The lack of interleaved expansions is consistent with species-specific expansions of MMPs, implying relatively little evolution of MMP along the *Gonium/Volvox* lineage after divergence from the *Chlamydomonas* lineage.

Analysis of Pherophorin Genes

The vegetative cell wall or extracellular matrix (ECM) of volvocine algae is composed of glycoproteins, lacking simple polysaccharides such as cellulose. Most of the characterized glycoproteins are hydroxyproline-rich glycoproteins (HRGPs), in which a large portion of the protein consists of simple (hydroxy)proline-rich repeat units.

Volvocine cell walls are all divided into three parts[4]: the inner part (W1 in *Chlamydomonas*[70]) varies in thickness among species[4], filling the space between the plasma membrane and the central layer. In some species, such as species of *Eudorina*, *Pleodorina*, or *Volvox* (Figure 1), this space can make up much of the volume of a colony. The central layer (also known as the "tripartite boundary", is composed of three sub-layers: W2, W4, W6) has a fairly uniform structure among species. The outermost (also referred to as the "capsule") layer is adjacent to the environment (W7) and is also variable in thickness among species. In *Chlamydomonas* and *Gonium*, the central layer surrounds each cell. In the *Volvocaceae* (including *Pandorina*, *Eudorina*, *Pleodorina*, and *Volvox*; Supplementary Figure 1), it has instead become a component of the colony wall. In *Gonium* colonies the central layer fuses in a bridge structure where adjacent cells join[71].

The bulk of ECM biochemistry and molecular biology studies come from *Chlamydomonas reinhardtii* and *Volvox carteri*. The central layer in *Chlamydomonas* was shown to consist of three sub-layers: W2, W4, W6. The innermost, W2, is a thick weave of covalently cross-linked fibers. W4 is a sub-layer of large granules, and the outer W6 sub-layer, which has two crystalline sub-sub-layers: an inner W6a and outer W6b[21,70]. The W4 and W6 sub-layers can be solubilized in chaotropic salt solutions, allowing purification and biochemical analysis of the individual proteins. A glycine-rich protein called GP1.5 comprises the W4 sub-layer. The W6 sub-layer is comprised of three proteins (GP1, GP2, and GP3), which are all HRGPs whose genes have since been characterized[21,70,72].

If the salt-soluble proteins are mixed with the *Chlamydomonas* cell ghosts (cell structures remaining after the W4 and W6 sub-layers are removed), and the salt removed by dialysis, then the proteins self-assemble back onto the W2 sub-layer. *Chlamydomonas* soluble proteins will also reassemble onto the W2 sub-layer of *Gonium* colonies and

Volvox spheroids whose W4 and W6 sub-layers have been removed by salt extraction[21], suggesting the assembly of the central layer is well conserved among volvocine algae.

Though the genes underlying the W2 and W4 sub-layers have not been identified, the genes which produce W6 sub-layer proteins are known. GP2 and GP3 comprise the W6a sub-layer and are present in both *Chlamydomonas* and *Volvox*[72]. The GP1 protein assembles as the W6b sub-sub-layer in *Chlamydomonas*, but GP1 appears to be missing from *Volvox*, based on electron microscopy, protein analysis[70], and genomic analysis[19]. The effect of this GP1 absence in *Volvox* is unknown.

In comparing *Chlamydomonas* and *Volvox* (Figure 1), the much larger size of *Volvox* is substantially due to the extracellular matrix (ECM). *Volvox* ECM is largely composed of pherophorin gene products[6]. Most pherophorin genes consist of two pherophorin domains (Pfam DUF3707, approximately 150 amino acids long), and are connected by a variable length hydroxyproline-rich repeat.

Six *Chlamydomonas* pherophorins (originally pheroC1-6, then phC1-6[19]) were previously identified using *Volvox* pherophorin cDNA sequences to probe *Chlamydomonas* genomic libraries[73]. No immunolocalization studies have been done in *Chlamydomonas*, but pherophorins may be present in the W1, W2, and/or W7 sub-layers. Three *Chlamydomonas* pherophorins have also been identified based on mRNA upregulation during N-starvation (*GAS28*, *GAS30*, *GAS31*[74]).

Most pherophorins have been determined using genomic approaches[19]. Consistent with their role in ECM production, the genome of *Volvox* demonstrated a substantial pherophorin expansion (49 genes) relative to *Chlamydomonas* (29 genes)[19]. Using the annotated pherophorins from *Volvox* version 1 (from US Department of Energy, Joint Genomes Institute), *Chlamydomonas* version 3 (from US National Center for Biotechnology Information, Genbank), and *Chlamydomonas* version 4 (from US Department of Energy, Joint Genomes Institute), we searched for, collected, and manually built gene models of pherophorins in *Chlamydomonas* version 5.3, *Gonium*, and *Volvox* version 2. Manual modifications of computer annotations were made to improve the pherophorin domains flanking a proline-rich repeat.

Our pherophorin models for *Chlamydomonas* version 5.3 are very similar to previous results[19] with few novel models and a few incomplete models removed (*phC11*, *phC23*, *phC24*, *phC25*) due to coalescence into complete models. For *Volvox*, when both models from version 1 and version 2 were utilized, the quality of genome assembly in *Volvox* version 2 is much improved. This improvement is particularly relevant for the pherophorin gene family as the repetitive hydroxyproline rich repeat between the N and C terminus domains is difficult to assemble, which results in incomplete and inaccurate gene models (demonstrated by N or C terminus domains immediately adjacent to assembly gaps or the end of a contig). Because of this, higher quality assemblies include additional tandem pherophorin expansions such as the *phV40* expansion (Supplementary Data 3).

For newly discovered tandem duplications of pherophorin genes (including *Chlamydomonas*, *Gonium*, and *Volvox*), a lettered convention (*e.g.*, *phC20a*, *phC20b*, and *phC20c* in *Chlamydomonas*) was used rather than adding new numbers; names of previously annotated tandem gene duplications were not modified. The tandem gene labeled '*a*' corresponds to the original gene (*e.g.*, *phC16a* was *phC16*).

All annotated pherophorin genes have at least one, usually two and sometimes up to four, Pfam DUF3707 domains. After manual model building and removal of duplicate gene models, the number of pherophorin genes increased in both *Chlamydomonas* (35) and *Volvox* (78), maintaining a similar 1:2 ratio of number of pherophorin genes[19]. In the *Gonium* genome, we were able to identify 31 complete pherophorin genes (named *phG1*-*phG26*, *GAS28*, *GAS30*, *GAS31*). Two of these genes (*phG22*, *GAS28*) have gaps between the two DUF3707 domains (likely an assembly complication based on proline repeats) resulting in two domains on different assembly scaffolds. These genes were assembled based on syntenic relationship of flanking genes in the *Chlamydomonas* genome (Supplementary Data 3).

Previously performed RT-PCR using degenerate primers, has yielded short fragments of four *Gonium* pherophorins (pheroG1-4[73]). We did not name *phG1-4* to correspond to these fragments. These four sequences are similar to the pherophorin clade in *Gonium* that contains *phG2a*, *phG2b*, *phG5a*, *phG5b*, *phG6*, *phG23*, *phG24*, and *phG25* (Supplementary Figure 27); differences in models may represent different *Gonium* strains or differences in model building. Removing primer sequences from previous sequences does not further reveal orthology. Similar to *Volvox*, we were not able to identify a *GP1* gene, though *GP2* and *GP3* are well conserved among *Chlamydomonas*, *Gonium*, and *Volvox* (Supplementary Figure 28), suggesting the *GP1* gene is unique to *Chlamydomonas*. Given the absence of *GP1* in both *Gonium* and *Volvox*, it is unknown whether this represents a loss in colonial/multicellular species or an innovation in *Chlamydomonas*.

A phylogenetic tree, using full gene alignments, predicts substantial species-specific innovation, especially in *Volvox* (Supplementary Figure 27), which is consistent with the tandem synteny present in many genes (Supplementary Data 3). Lastly, there are a number of genes in all species (*Chlamydomonas*, *Gonium*, *Volvox*) that contain pherophorin domains that have not been included. These genes appear distantly related and generally have marginally significant E-values (10^{-4}) for Pfam DUF3707 domains. The current tree contains all known pherophorin genes that have been experimentally studied.

A preliminary investigation of signatures of selection within the metalloprotease and pherophorin family was performed on several clades (metalloprotease, four; pherophorin, five) of the entire metalloprotease and pherophorin family trees (as labeled in Supplementary Figure 42 and 27). Supported clades where genes from each species form a monophyletic clade were selected (some clades include a single species, others include *Chlamydomonas*, *Gonium*, and *Volvox*). Pairwise dN, dS, and dN/dS values were predicted using PAML[37] while providing a codon-based nucleotide alignment (Supplementary Figure 29). These selected clades largely demonstrate stabilizing selection amongst metalloprotease and pherophorin gene expansions (Supplementary Figure 29). When stabilizing selection is operating on gene family expansions, gene dosage may be maintaining these gene duplications. Given that a large amount of metalloprotease and pherophorin protein product are likely necessary to produce, maintain, and repair the ECM in *Volvox*, gene dosage (i.e., increasing the total metalloprotease or pherophorin product) may be the underlying mechanism[75,76]. Several pairwise comparisons are predicted to have high dN/dS values (>3), which suggests positive selection (i.e., MMP clade 4, Supplementary Figure 29). If so, these genes may

be experiencing neofunctionalization related to the evolution of multicellularity[77,78] however further detailed analyses, including expression data, are necessary.

Phylogenetic Analyses

Unless otherwise stated, all phylogenetic analyses were performed using a custom pipeline of SATe version 2.2.7[79] coupled with RAxML version 8[31]. Full gene protein sequences were passed to SATe using a FASTTREE tree estimation with a RAxML search after tree formation with a maximum limit of 10 iterations and the "longest" decomposition strategy. Bootstraps were made on the SATe output alignment and tree using RAxML with automatic model selection, a rapid hill climbing algorithm (-f d) and 100 bootstrap partitions. Bipartition information (-f a) was obtained using the SATe output tree and RAxML bootstraps.

***Chlamydomonas* strains culture conditions**

Wild-type *Chlamydomonas reinhardtii* 6145 and 21gr, and *HA-CrRB* (*HA-MAT3::mat3-4*, here referred to as *HA-CrRB::rb*), *mat3-4* (here referred to as *rb*), and *dp1* have been previously described[46,80,81]. Briefly, wild-type strains 6145 (MT-) and 21gr (MT+) are mating pairs that have been back crossed to eliminate the *y1* mutation in 6145[81]. The *RB* knockout strain has been previously characterized as a null allele, and the knockout mutation is the *rb* allele[46,80]. The *rb* mutation can be complemented by a N-terminally tagged version of the gene that behaves identical to wild-type,. Previously a knockout mutation in the *Chlamydomonas DP1* gene, *dp1*, was identified and characterized[46,81]. All strains were maintained on TAP plates. For phenotype analysis, strains were grown in high salt media (HSM) synchronously under 14 hours of 150 μE of light, samples were fixed hourly and examined by light microscopy[46,81].

Cloning of *Gonium pectorale RB* and transformation into *rb*

A 3X haemagluttin (HA) tagged copy of the *Gonium pectorale RB* gene was cloned using InFusion Cloning (Clontech) to be driven by the *Chlamydomonas RB* promoter and terminator that includes a *AphVIII* selectable marker for *Chlamydomonas* transformation (Fig. 4, [46]). *Gonium pectorale* genomic DNA from K4F3 was used as a template and the genomic region of *RB* was amplified without its ATG start codon using the primers 5'-CAGATTACGCTACTAGATCTGCCGAAGCTGAACGTTTTACTGCG-3', and 5'-CTCCGGCCGCGGTGCCTAATTTGCGCCGTACCGCCGGA-3'. These primers overlap with the 3X HA tag and 3' terminator from the previously created HA-CrRB transformation clone that complements the *rb* mutation[46]. The HA-CrRB plasmid was amplified by inverse PCR with 5'-TCTAGTAGCGTAATCTGGAACGTCATATGGATAGG-3' and 5'-GCACCGCGGCCGGAGGT-3' primers. PCR products were gel purified with a QiaQuick gel extraction kit (Qiagen). Purified PCR fragments were fused by InFusion (Clontech) cloning based on overlaps in the amplified sequences and transformed into chemically competent DH5-apha cells, after which the clone was confirmed by sequencing.

Transformation of *Chlamydomonas reinhardtii*

The *rb* strain was transformed with the glass beads[46], with the *HA-GpRB* clone (above) and as a control with *HA-CrRB* and pSI103 (*AphVIII* selectable marker only) and selected on TAP plates supplemented with 20 µg/mL paromycin[46]. Candidate strains were screened by growth morphology[46,81], and then screened for expression by immunoblotting with an anti-HA antibody (Roche 3F10, high affinity[46]). Four independent strains expressing the *HA-GpRB*, and five independent strains expressing *HA-CrRB* were created. Control complementation of the *rb* mutation with *HA-CrRB* occurred at rates similar to previous results[46]. The presence of the *rb* mutation was confirmed by replica plating on TAP plates supplemented with 10 µg/mL emetine[46,80].

Genetic analysis of *HA-GpRB* expressing strains

Two lines expressing *HA-GpRB* were crossed to a *dp1* null mutation[81]. Because both the *HA-GpRB* and *dp1* mutations are linked to *AphVIII,* single tetrads were dissected[82]. *HA-GpRB* was genotyped with primers in the 3XHA tag 5'-AGTGCTAACAGCATGTCTAGTTAC-3', and in the 5' portion of *GpRB* 5'-TGCGAACAACCGCTGCAGACCTTC-3'. The *dp1* mutation was genotyped as previously described[81].

Immunoblotting *HA-GpRB* and *HA-CrRB* strains complementing *rb*

Whole cell lysates from strains were prepared, separated, and immunoblotted[46]. Briefly, the anti-HA antibody used for detection of *HA-GpRB* and *HA-CrRB* was an anti-HA high affinity monoclonal antibody (clone 3F10, Roche), and anti-alpha-tubulin monoclonal antibody (Sigma) as previously described[46]. The expression levels of *RB* in *HA-CrRB* strains have been previously shown to be similar to wild type *Chlamydomonas* expression levels[46]. The expression levels of *RB* in *HA-GpRB* are similar, if not slightly below, the expression levels of *HA-CrRB*, suggesting that overexpression of *RB* is not causing the observed colonial phenotype, but rather modification to the *Gonium RB* gene.

Measurement of cell or colony size distribution

The size of cells and groups of cells was measured with a Moxi Z automated cell sizer/counter using type "S" cassettes (ORFLO Technologies). Sizing is based on the Coulter principle used previously with *Chlamydomonas reinhardtii*[46,81].

Supplementary References

1. Herron, M. D. & Michod, R. E. Evolution of complexity in the volvocine algae: transitions in individuality through Darwin's eye. *Evolution (N. Y).* **62,** 436–451 (2008).
2. Isaka, N., Kawai-Toyooka, H., Matsuzaki, R., Nakada, T. & Nozaki, H. Description of Two New Monoecious Species of Volvox Sect. Volvox (Volvocaceae, Chlorophyceae), Based on Comparative Morphology and Molecular Phylogeny of Cultured Material1. *J. Phycol.* **48,** 759–767 (2012).
3. Nozaki, H., Yamada, T. K., Takahashi, F., Matsuzaki, R. & Nakada, T. New 'missing link' genus of the colonial volvocine green algae gives insights into the evolution of oogamy. *BMC Evol. Biol.* **14,** 37–47 (2014).
4. Coleman, A. W. A Comparative analysis of the Volvocaceae (Chlorophyta). *J. Phycol.* **48,** 491–513 (2012).
5. Ferris, P. *et al.* SOM Evolution of an expanded sex-determining locus in Volvox. *Science* **328,** 351–4 (2010).
6. Hallmann, A. Extracellular matrix and sex-inducing pheromone in Volvox. *Int. Rev. Cytol.* **227,** 131–182 (2003).
7. Prochnik, S. E. *et al.* Genomic analysis of organismal complexity in the multicellular green alga Volvox carteri. *Science* **329,** 223–6 (2010).
8. Miller, S. M., Schmitt, R. & Kirkai, D. L. Jordan , an Active Volvox Transposable Element Similar to Higher Plant Transposons. *Plant Cell* **5,** 1125–1138 (1993).
9. Stanke, M. & Morgenstern, B. AUGUSTUS: a web server for gene prediction in eukaryotes that allows user-defined constraints. *Nucleic Acids Res.* **33,** W465–7 (2005).
10. Trapnell, C., Pachter, L. & Salzberg, S. L. TopHat: discovering splice junctions with RNA-Seq. *Bioinformatics* **25,** 1105–11 (2009).
11. Langmead, B., Trapnell, C., Pop, M. & Salzberg, S. L. Ultrafast and memory-efficient alignment of short DNA sequences to the human genome. *Genome Biol.* **10,** R25 (2009).
12. Moreau, H. *et al.* Gene functionalities and genome structure in Bathycoccus prasinos reflect cellular specializations at the base of the green lineage. *Genome Biol.* **13,** R74 (2012).
13. Merchant, S. S. *et al.* The Chlamydomonas genome reveals the evolution of key animal and plant functions. *Science* **318,** 245–50 (2007).
14. Blanc, G. *et al.* The Chlorella variabilis NC64A genome reveals adaptation to photosymbiosis, coevolution with viruses, and cryptic sex. *Plant Cell* **22,** 2943–55 (2010).

15. Blanc, G. *et al.* The genome of the polar eukaryotic microalga Coccomyxa subellipsoidea reveals traits of cold adaptation. *Genome Biol.* **13,** R39 (2012).

16. Worden, A. Z. *et al.* Green Evolution and Dynamic Adaptations Revealed by Genomes of the Marine Picoeukaryotes Micromonas. *Science (80-.).* **324,** 268–272 (2009).

17. Derelle, E. *et al.* Genome analysis of the smallest free-living eukaryote Ostreococcus tauri unveils many unique features. *Proc. Natl. Acad. Sci. U. S. A.* **103,** 11647–52 (2006).

18. Palenik, B. *et al.* The tiny eukaryote Ostreococcus provides genomic insights into the paradox of plankton speciation. *Proc. Natl. Acad. Sci. U. S. A.* **104,** 7705–7710 (2007).

19. Prochnik, S. E. *et al.* Genomic analysis of organismal complexity in the multicellular green alga Volvox carteri. *Science (80-.).* **329,** 223–226 (2010).

20. Gilles, R., Gilles, C. & Jaenicke, L. Sexual differentiation of the green alga Volvox carteri. *Naturwissenschaften* **70,** 571–572 (1983).

21. Adair, W. S. & Snell, W. J. in *Organ. Assem. Plant Anim. Extracell. Matrix* (Adair, W. S. & Mecham, R. P.) 15–84 (Academic Press, 1990).

22. Fellows, I. Deducer: A Data Analysis GUI for R. *J. Stat. Softw.* **49,** (2012).

23. Guo, A.-Y. *et al.* PlantTFDB: a comprehensive plant transcription factor database. *Nucleic Acids Res.* **36,** D966–9 (2008).

24. Riaño-Pachón, D. M., Ruzicic, S., Dreyer, I. & Mueller-Roeber, B. PlnTFDB: an integrative plant transcription factor database. *BMC Bioinformatics* **8,** 42 (2007).

25. Richardt, S., Lang, D., Reski, R., Frank, W. & Rensing, S. a. PlanTAPDB, a phylogeny-based resource of plant transcription-associated proteins. *Plant Physiol.* **143,** 1452–66 (2007).

26. Finn, R. D. *et al.* The Pfam protein families database. *Nucleic Acids Res.* **36,** D281–8 (2008).

27. Finn, R. D. *et al.* The Pfam protein families database. *Nucleic Acids Res.* **38,** D211–22 (2010).

28. Eddy, S. R. Profile hidden Markov models. *Bioinformatics* **14,** 755–763 (1998).

29. Li, L., Stoeckert, C. J. & Roos, D. S. OrthoMCL: identification of ortholog groups for eukaryotic genomes. *Genome Res.* **13,** 2178–89 (2003).

30. Edgar, R. C. MUSCLE: multiple sequence alignment with high accuracy and high throughput. *Nucleic Acids Res.* **32,** 1792–7 (2004).

31. Stamatakis, A. RAxML version 8: a tool for phylogenetic analysis and post-analysis of large phylogenies. *Bioinformatics* **30,** 1312–3 (2014).

32. Worden, A. Z. & Not, F. in *Microb. Ecol. Ocean* (Kirchman, D.) (Wiley, 2008).

33. Nozaki, H. Origin and evolution of the genera Pleodorina and Volvox (Volvocales). *Biologia (Bratisl).* **58,** 425–431 (2003).

34. Leliaert, F. *et al.* Phylogeny and Molecular Evolution of the Green Algae. *CRC. Crit. Rev. Plant Sci.* **31,** 1–46 (2012).

35. Nozaki, H. & Itoh, M. Phylogenetic relationships within the colonial Volvocales (Chlorophyta) inferred from cladistic analysis based on morphological data. *J. Phycol.* **30,** 353–365 (1994).

36. Csurös, M. Count: evolutionary analysis of phylogenetic profiles with parsimony and likelihood. *Bioinformatics* **26,** 1910–1912 (2010).

37. Yang, Z. PAML 4: Phylogenetic analysis by maximum likelihood. *Mol. Biol. Evol.* **24,** 1586–1591 (2007).

38. Herron, M. D., Hackett, J. D., Aylward, F. O. & Michod, R. E. Triassic origin and early radiation of multicellular volvocine algae. *Proc. Natl. Acad. Sci. USA* **106,** 3254–3258 (2009).

39. Domazet-Loso, T., Brajkovic, J. & Tautz, D. A phylostratigraphy approach to uncover the genomic history of major adaptations in metazoan lineages. *Trends Genet.* **23,** 533–539 (2007).

40. Altschul, S. *et al.* Gapped BLAST and PSI-BLAST: a new generation of protein database search programs. *Nucleic acids Res* **25,** 3389–3402 (1997).

41. Bišová, K., Krylov, D. M. & Umen, J. G. Genome-wide annotation and expression profiling of cell cycle regulatory genes in Chlamydomonas reinhardtii. *Plant Physiol.* **137,** 475–491 (2005).

42. Hammesfahr, B., Odronitz, F., Mühlhausen, S., Waack, S. & Kollmar, M. GenePainter: a fast tool for aligning gene structures of eukaryotic protein families, visualizing the alignments and mapping gene structures onto protein structures. *BMC Bioinformatics* **14,** 77 (2013).

43. Krauss, V. *et al.* Near intron positions are reliable phylogenetic markers: An application to holometabolous insects. *Mol. Biol. Evol.* **25,** 821–830 (2008).

44. Ferris, P. J. *et al.* Evolution of an expanded sex-determining locus in Volvox. *Science (80-.).* **328,** 351–354 (2010).

45. Hiraide, R. *et al.* The evolution of male-female sexual dimorphism predates the gender-based divergence of the mating locus gene MAT3/RB. *Mol. Biol. Evol.* **30,** 1038–1040 (2013).

46. Olson, B. J. S. C. *et al.* Regulation of the Chlamydomonas cell cycle by a stable, chromatin-associated retinoblastoma tumor suppressor complex. *Plant Cell* **22,** 3331–3347 (2010).

47. Duncan, L. *et al.* The VARL gene family and the evolutionary origins of the master cell-type regulatory gene, regA, in Volvox carteri. *J. Mol. Evol.* **65,** 1–11 (2007).

48. Kirk, M. M. *et al.* regA, a Volvox gene that plays a central role in germ-soma differentiation, encodes a novel regulatory protein. *Development* **126,** 639–47 (1999).

49. Nishii, I. & Miller, S. M. Volvox: Simple steps to developmental complexity? *Curr. Opin. Plant Biol.* **13,** 646–653 (2010).

50. Meissner, M., Stark, K., Cresnar, B., Kirk, D. L. & Schmitt, R. Volvox germline-specific genes that are putative targets of RegA repression encode chloroplast proteins. *Curr. Genet.* **36,** 363–370 (1999).

51. Nedelcu, A. M. & Michod, R. E. The evolutionary origin of an altruistic gene. *Mol. Biol. Evol.* **23,** 1460–1464 (2006).

52. Nedelcu, A. M. Environmentally induced responses co-opted for reproductive altruism. *Biol. Lett.* **5,** 805–8 (2009).

53. Hanschen, E. R., Ferris, P. J. & Michod, R. E. Early evolution of the genetic basis for soma in the Volvocaceae. *Evolution (N. Y).* **68,** 2014–2025 (2014).

54. Duncan, L., Nishii, I., Howard, A., Kirk, D. & Miller, S. M. Orthologs and paralogs of regA, a master cell-type regulatory gene in Volvox carteri. *Curr. Genet.* **50,** 61–72 (2006).

55. Letunic, I., Doerks, T. & Bork, P. SMART 7: recent updates to the protein domain annotation resource. *Nucleic Acids Res.* **40,** D302–305 (2012).

56. Katoh, K., Kuma, K., Toh, H. & Miyata, T. MAFFT version 5: improvement in accuracy of multiple sequence alignment. *Nucleic Acids Res.* **33,** 511–8 (2005).

57. Kirk, D. L. *Volvox: Molecular-Genetic Origins of Multicellularity and Cellular Differentiation*. (Cambridge University Press, 1998).

58. Koufopanou, V. The evolution of soma in the Volvocales. *Am. Nat.* **143,** 907–931 (1994).

59. Solari, C. A., Kessler, J. O. & Michod, R. E. A hydrodynamics approach to the evolution of multicellularity: flagellar motility and germ-soma differentiation in volvocalean green algae. *Am. Nat.* **167,** 537–554 (2006).

60. Nozaki, H. Morphology and taxonomy of two species of Astrephomene (Chlorophyta) in Japan. *Journ. Jap. Bot.* **58,** 345–352 (1983).

61. Pocock, M. A. Two multicellular motile green algae, Volvulina Playfair and Astrephomene, a new genus. *Trans. R. Soc. South Africa* **34,** 103–127 (1954).

62. Kirk, D. L., Birchem, R. & King, N. The extracellular matrix of Volvox: a comparative study and proposed system of nomenclature. *J. Cell Sci.* **80,** 207–31 (1986).

63. Kirk, D. L. A twelve-step program for evolving multicellularity and a division of labor. *BioEssays* **27,** 299–310 (2005).

64. Umen, J. G. & Olson, B. J. S. C. in *Adv. Bot. Res.* **64,** 185–243 (Elsevier, 2012).

65. Bell, G. in *Orig. Evol. Sex* (Halvorson, H. O. & Monroy, A.) 221–256 (Alan R. Liss, 1985).

66. Koufopanou, V. & Bell, G. Soma and germ: an experimental approach using Volvox. *Proc. R. Soc. London B Biol. Sci.* **254,** 107–113 (1993).

67. Heitzer, M. & Hallmann, A. An extracellular matrix-localized metalloproteinase with an exceptional QEXXH metal binding site prefers copper for catalytic activity. *J. Biol. Chem.* **277,** 28280–6 (2002).

68. Kubo, T., Saito, T., Fukuzawa, H. & Matsuda, Y. Two tandemly-located matrix metalloprotease genes with different expression patterns in the Chlamydomonas sexual cell cycle. *Curr. Genet.* **40,** 288–289 (2001).

69. Herron, M. D., Desnitskiy, A. G. & Michod, R. E. Evolution of developmental programs in Volvox (Chlorophyta). *J. Phycol.* **46,** 316–324 (2010).

70. Woessner, J. P. & Goodenough, U. W. Volvocine cell walls and their constituent glycoproteins : an evolutionary perspective. *Protoplasma* **181,** 245–258 (1994).

71. Nozaki, H. Ultrastructure of the extracellular matrix of Gonium (Volvocales, Chlorophyta). *Phycologia* **29,** 1–8 (1990).

72. Voigt, J., Kiess, M., Getzlaff, R., Wöstemeyer, J. & Frank, R. Generation of the heterodimeric precursor GP3 of the Chlamydomonas cell wall. *Mol. Microbiol.* **77,** 1512–26 (2010).

73. Hallmann, A. The pherophorins: common, versatile building blocks in the evolution of extracellular matrix architecture in Volvocales. *Plant J.* **45,** 292–307 (2006).

74. Hoffmann, X. & Beck, C. F. Mating-Induced Shedding of Cell Walls, Removal of Walls from Vegetative Cells, and Osmotic Stress Induce Presumed Cell Wall Genes in Chlamydomonas. *Plant Physiol.* **139,** 999–1014 (2005).

75. Kondrashov, F. A. & Kondrashov, A. S. Role of selection in fixation of gene duplications. *J. Theor. Biol.* **239,** 141–51 (2006).

76. Kondrashov, F. A., Rogozin, I. B., Wolf, Y. I. & Koonin, E. V. Selection in the evolution of gene duplications. *Genome Biol.* **3,** RESEARCH0008 (2002).

77. Beisswanger, S. & Stephan, W. Evidence that strong positive selection drives neofunctionalization in the tandemly duplicated polyhomeotic genes in Drosophila. *Proc. Natl. Acad. Sci. U. S. A.* **105,** 5447–5452 (2008).

78. Osada, N. & Innan, H. Duplication and gene conversion in the Drosophila melanogaster genome. *PLoS Genet.* **4,** e1000305 (2008).

79. Liu, K., Raghavan, S., Nelesen, S., Linder, C. R. & Warnow, T. Rapid and accurate large-scale coestimation of sequence alignments and phylogenetic trees. *Science* **324,** 1561–1564 (2009).

80. Umen, J. G. & Goodenough, U. W. Control of cell division by a retinoblastoma protein homolog in Chlamydomonas. *Genes Dev.* **15,** 1652–61 (2001).

81. Fang, S.-C., de los Reyes, C. & Umen, J. G. Cell size checkpoint control by the retinoblastoma tumor suppressor pathway. *PLoS Genet.* **2,** e167 (2006).

82. Harris, E. H. *The Chlamydomonas Sourcebook (Volume 1)*. (Acad, 2009).
